# Accelerated microfluidic native chemical ligation at difficult amino acids toward cyclic peptides

**DOI:** 10.1038/s41467-018-05264-8

**Published:** 2018-07-20

**Authors:** Nathalie Ollivier, Thomas Toupy, Ruben C. Hartkoorn, Rémi Desmet, Jean-Christophe M. Monbaliu, Oleg Melnyk

**Affiliations:** 1 UMR CNRS 8204, Chemical Biology of Flatworms, Institut Pasteur de Lille, Université de Lille, 1 rue du Pr Calmette, 59021 Lille Cedex, France; 2 Department of Chemistry, RU Molecular Systems, Center for Integrated Technology and Organic Synthesis, University of Liège, B-4000 Liège (Sart Tilman), Belgium; 3 INSERM U1019 UMR CNRS 8204, Chemical Biology of Antibiotics, Institut Pasteur de Lille, Université de Lille, 1 rue du Pr Calmette, 59021 Lille Cedex, France

## Abstract

Cyclic peptide-based therapeutics have a promising growth forecast that justifies the development of microfluidic systems dedicated to their production, in phase with the actual transitioning toward continuous flow and microfluidic technologies for pharmaceutical production. The application of the most popular method for peptide cyclization in water, i.e., native chemical ligation, under microfluidic conditions is still unexplored. Herein, we report a general strategy for fast and efficient peptide cyclization using native chemical ligation under homogeneous microfluidic conditions. The strategy relies on a multistep sequence that concatenates the formation of highly reactive *S*-(2-((2-sulfanylethyl)amino)ethyl) peptidyl thioesters from stable peptide amide precursors with an intramolecular ligation step. With very fast ligation rates (<5 min), even for the most difficult junctions (including threonine, valine, isoleucine, or proline), this technology opens the door toward the scale-independent, expedient preparation of bioactive macrocyclic peptides.

## Abstract (web summary)

Flow-based peptide synthesis is a well-established method, yet difficult to combine with native chemical ligation (NCL), the go-to method for peptide cyclization. Here, the authors developed a microfluidic procedure for peptide cyclization within minutes, using NCL and an SEA alkylthioester peptide.

## Introduction

**Fig. 1:**
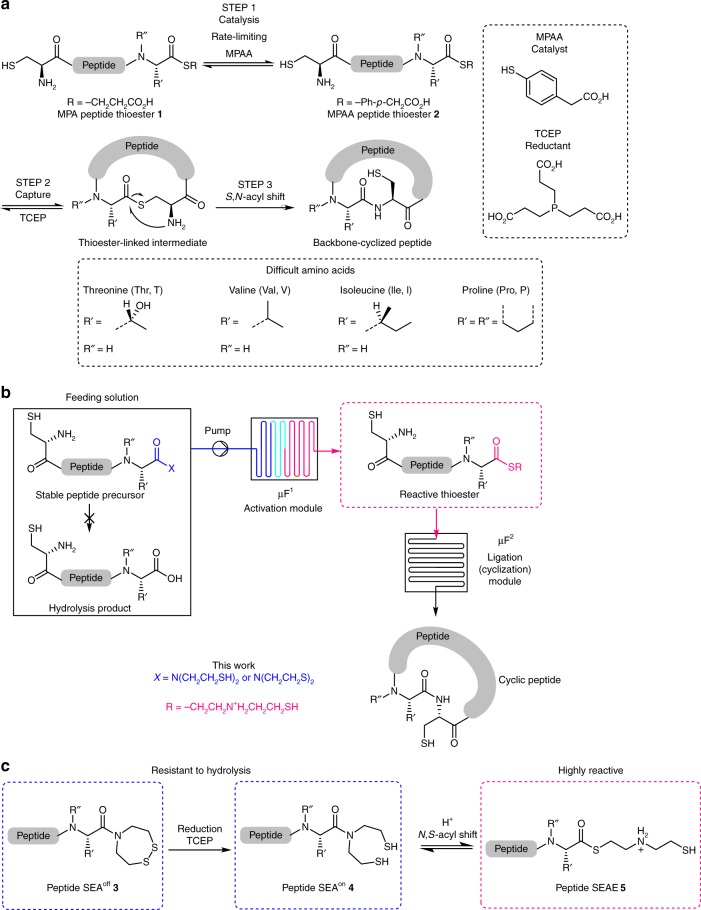
Cyclic peptide synthesis using NCL and its implementation under microfluidic conditions. **a** Principle of NCL reaction applied to cyclic peptide synthesis. The NCL reaction involves the chemoselective reaction of a C-terminal peptide alkylthioester (e.g., MPA peptide thioester **1**) or arylthioester (e.g., MPAA peptide thioester **2**) with an *N*-terminal cysteinyl peptide. The intramolecular version of this reaction enables backbone cyclization. The ligation proceeds in the presence of an exogenous thiol catalyst (typically MPAA) and a reductant (typically TCEP). **b** Implementation of NCL under microfluidic conditions toward backbone peptide cyclization. The system is fed with a stable precursor, which is activated into a highly reactive thioester species before entering the ligation module. **c** Reactive SEAE peptide thioesters are generated from the stable SEA cyclic disulfide (SEA^off^) **3** upon reduction with TCEP or from the reduced SEA^on^ bisthiol **4**. SEAE peptide thioesters **5** enable extremely fast NCL even with difficult C-terminal amino acids

Peptide drugs are an important and fast growing class of pharmaceuticals^1–3^, the development of which is stimulated by (a) the discovery of innovative production methods^1^, (b) the design of novel delivery systems^4^, as well as (c) strategies for improving their pharmaco-kinetic properties^2,5^. In particular, cyclic peptides have been attracting considerable attention for several decades since they enable higher potency, metabolic stability, and oral bioavailability than their linear counterparts^6,7^, and several of them are already in clinical use^8^. A large variety of synthetic methods for producing cyclic peptide scaffolds are now available^9,10^. In particular, the two-step process based on the synthesis of linear peptide precursors by solid phase peptide synthesis (SPPS^11^) followed by chemoselective cyclization in water using either native chemical ligation (NCL^12–14^), ketoacid hydroxylamine ligation (KAHA^15^), or serine/threonine ligation (STL^16^) is particularly powerful. Among these methods, the backbone peptide cyclization using NCL is one of the most popular^17^. The NCL reaction involves a sequence of reversible thiol/thioester exchanges, starting from a peptide alkylthioester, such as peptide thioester **1** derived from 3-mercaptopropionic acid (MPA, Fig. 1a). The sequence starts with the exchange between **1** and a catalyst, e.g., 4-mercaptophenylacetic acid (MPAA), and then with an N-terminal Cys-peptide to produce a transient thioester-linked intermediate. The latter undergoes an irreversible *S*-to-*N* acyl shift, leading to the formation of a native peptide bond to Cys. The intramolecular version of NCL applied to a peptide featuring an N-terminal Cys residue and a C-terminal thioester functionality results in the formation of a backbone cyclized peptide^17^. One limitation of NCL is the low reactivity of peptide thioesters containing C-terminal threonine (Thr, T), valine (Val, V), isoleucine (Ile, I), or proline (Pro, P) residues (Fig. 1a)^18^, which is reflected by the reluctance of peptide chemists to produce linear or cyclic peptides through the formation of such difficult junctions^19^. Therefore, the search for simple and fast ligation methods is a timely and significant goal that should streamline the production of complex peptides including cyclic architectures^20,21^.

In parallel to the development of chemoselective ligation techniques, the field of peptide synthesis has benefited from the emergence of microfluidic and continuous flow technologies for overcoming known synthetic limitations^22^, facilitating automation and enabling an accurate control over the process parameters^23,24^. Peptide synthesis using microfluidics comes with a range of inherent assets for meeting high pharmaceutical standards, such as cleaner and constant reaction profiles, fast lab-to-market transitions, higher space/time yields even for complex reaction sequences^25,26^, and on-demand pharmaceutical production^27^. Homogeneous microfluidic peptide synthesis by stepwise coupling of protected amino acids was pioneered by Watts in 2001^28^ and later extended by several groups with equivalent or superior performances than conventional batch strategies^29–31^. Oligopeptide macrocyclization by enthalpic activation of a linear precursor^31^ or the SPPS method^32–35^ were also transposed under microfluidic conditions. However, and despite a huge preparative potential, NCL has not yet been adapted under microfluidic conditions, whether for peptide cyclization or for other applications. A close examination of NCL requirements and general features emphasizes the complexity of transposing such ligation chemistry from batch to microfluidic operation: to be of interest, the system must be fed with solutions of stable precursors, while reaction kinetics within the system must be fast enough to be compatible with its intrinsic features and small internal dimensions. These two requirements are barely compatible with standard NCL since a classical peptide alkylthioester, such as **1** (Fig. 1a), will require extended ligation times, while a reactive arylthioester, such as **2** (Fig. 1a)^36^, will undergo significant hydrolysis in the feed before entering the microfluidic system^18^. A potential solution is to feed the system with a stable peptide thioester precursor that can be activated within a first microfluidic module, prior to entering the ligation module for triggering peptide cyclization (Fig. 1b). Importantly, the solution used for the activation step must not interfere with the NCL reaction, while the generated thioester must be a powerful acyl donor to enable ligation in a few minutes even for difficult junctions. The formation of difficult junctions in a few minutes was, however, never observed with the NCL reaction, irrespective of the acyl donor including *bis*(2-sulfanylethyl)amido (SEA) thioester surrogates of type **3** or **4** (Fig. 1c)^37^. Such fast kinetics could only be achieved with the diselenide selenoester ligation (DSL^21^) of preformed peptidyl selenophenyl esters with *bis*(selenocysteinyl)peptides.

Here, we report an effective solution for the cyclization of peptides under microfluidic conditions that relies on the enhanced reactivity of intermediate *S*-(2-((2-sulfanylethyl)amino)ethyl) peptidyl (SEAE) thioesters of type **5** (Fig. 1c). SEAE thioesters **5**, produced in situ from stable SEA thioester surrogates of type **3** or **4**, appear as a robust solution for the implementation of NCL under microfluidic conditions (Fig. 1b). Although not yet fully understood, the enhanced reactivity profile of SEAE peptide thioesters **5** toward NCL enables extremely fast cyclization (<5 min) at notoriously difficult junctions (Val, Thr, or Ile), as well as intra or intermolecular ligations with the least tractable Pro junction. The method enables the production of cyclic peptides of varying size (10-28 AA), including biologically active RTD-1, by the formation of diverse junctions (Val, Ile, Thr, Pro, Phe, Tyr, Leu). We present concrete solutions for taming and exploiting the enhanced reactivity of SEAE peptides, as well as for implementing NCL under microfluidic conditions with fast optimization of reaction parameters (temperature, residence time, pH, concentration, local stoichiometry). The microfluidic NCL method described herein is complementary to DSL by permitting the formation of peptide bonds to Cys. It enables the seamless production of tens of milligrams of cyclic peptide without special precautions, under fully automated operation easy to deploy, while guaranteeing a homogeneous purity profile.

## Results

### Evidence for the high reactivity of SEAE peptide thioesters

**Fig. 2:**
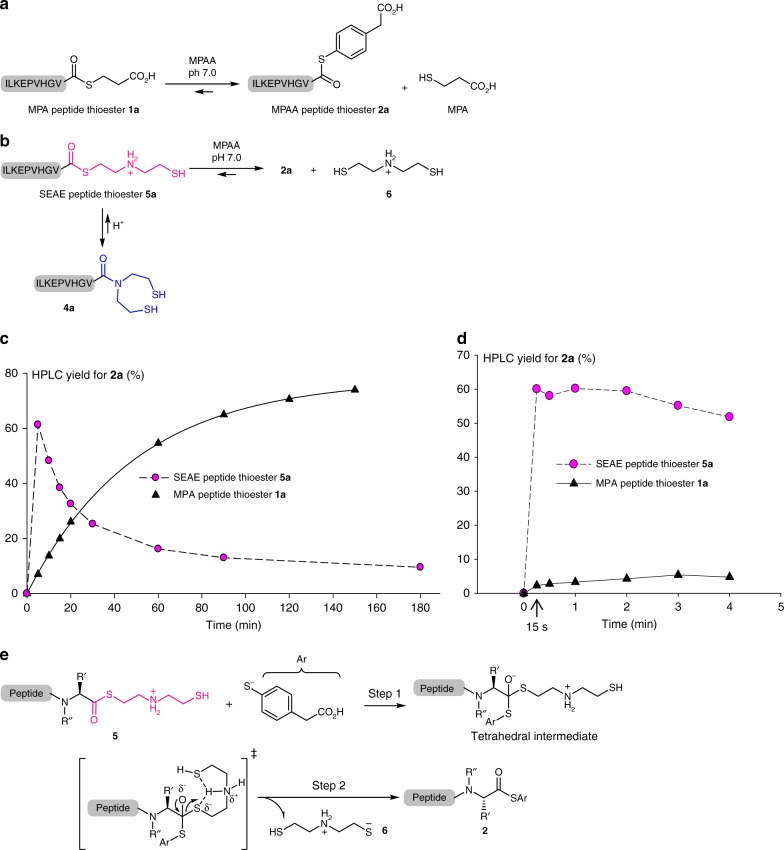
Evidence for the enhanced reactivity of SEAE peptide thioesters. **a** Principle of the thiol-thioester exchange between MPA peptide thioester **1a** and MPAA. **b** Principle of the thiol-thioester exchange between SEAE peptide thioester **5a** and MPAA. **c** Evidence for the high reactivity of model SEAE peptide **5a** (circles filled with magenta) featuring a C-terminal difficult amino acid (valine) in comparison with the classical MPA peptide thioester analog **1a** (filled triangles). The peptides were reacted in 0.2 M sodium phosphate buffer (pH 7.0) containing 50 mM TCEP and 400 mM MPAA at 37 °C. The exchange reaction was monitored by HPLC (UV detection at 215 nm). **d** Same reactions as in c under the optimal microfluidic conditions described in Fig. 4 (**1a**: black triangles, **5a**: magenta circles). **e** The high reactivity of SEAE peptide thioesters is potentially a consequence of an intramolecular acid-catalysis that facilitates departure of thiol **6** from the tetrahedral intermediate (step 2). This step is rate-limiting for simple alkylthiols such as MPA. R’ and R” correspond to the amino acid side-chain (see Fig. 1)

A typical batch experiment highlighting the difference in reactivity between classical MPA peptide thioesters and SEAE thioesters is the thiol-thioester exchange reaction with MPAA, which constitutes the rate-limiting step of NCL with peptide alkylthioesters (Fig. 2a, b). The yield for MPAA peptide thioester **2a** from preformed SEAE peptide **5a** was 60% within 5 min, while the reaction with MPA thioester **1a** required ca 80 min to reach a similar conversion (Fig. 2c, Supplementary Methods). In fact, further kinetic studies under microfluidic conditions showed that the conversion of SEAE peptide **5a** into MPAA thioester **2a** proceeded in less than 15 s, while the MPA peptide thioester analog **1a** furnished only ~2% of MPAA thioester **2a** after 15 s (Fig. 2d, Supplementary Methods). Note that in the absence of a Cys peptide, the SEA peptide amide **4a** acts as a thermodynamic sink according to Fig. 2b. Thus after an initial burst phase, MPAA thioester **2a** decreases over time. Note that the conversion of MPAA thioester **2** or SEAE peptide **5** back into SEA peptide **4** is greatly minimized under microfluidic conditions in the presence of the Cys partner. A similar contrasting reactivity was observed for peptide thioesters **1a** or **5a** upon NCL with a model Cys peptide in the presence of MPAA (Supplementary Fig. 1, Supplementary Methods). A likely rationalization for the enhanced reactivity of thioesters of type **5** involves the intramolecular neighboring assistance (acid-catalysis) of the ammonium group for the departure of thiolate **6** (Fig. 2e). This hypothesis relies on the fact that the breakdown from the tetrahedral intermediate, i.e., step 2 in Fig. 2e, is typically rate-limiting with simple alkylthiols such as MPA^38^.

### Establishing feasibility

**Fig. 3:**
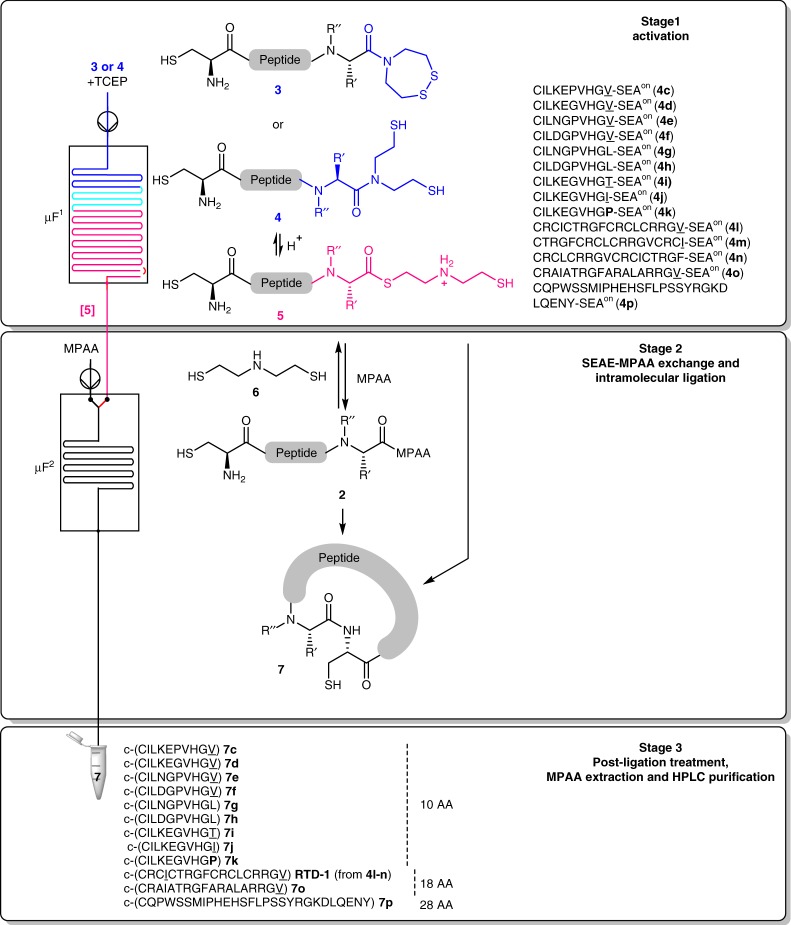
Fast intramolecular NCL toward macrocyclic peptide constructs. Details of the three-stage (activation–ligation–post ligation treatment) microfluidic process allowing intramolecular NCL at non-problematic junctions (Leu (L), R′ = −CH_2_CH(CH_3_)_2_, R′′ = H; Tyr (Y), R′ = −CH_2_Ph-*p*-OH, R′′ = H), difficult junctions (underlined, Val (V): R′ = −CH(CH_3_)_2_, R′′ = H; Thr (T): R′ = −CH(CH_3_)(OH), R′′ = H; Ile (I): R′ = −CH(CH_3_)(CH_2_CH_3_), R′′ = H) and intractable proline junction (bold, Pro (P): R′ = R′′ = −(CH_2_)_3_−). Two microfluidic modules (µF) are concatenated. In microreactor element µF^1^ (Activation, stage 1), the feed solution containing an inactive and stable peptide precursor, SEA^off^ **3** or SEA^on^ **4**, is thermally rearranged at pH 1 to give reactive SEAE thioester **5**. In microreactor element µF^2^ (SEAE-MPAA exchange and intramolecular ligation, stage 2), reactive SEAE thioester **5** cyclizes directly into peptide **7** or is converted into MPAA thioester **2** which then cyclizes into peptide **7**. Depending on the conditions, amine dithiol **6** can become a competing nucleophile and convert MPAA thioester **2** back to SEA^on^ peptide **4**. The last stage (Post ligation treatment, MPAA extraction and HPLC purification, stage 3) enables the isolation of the cyclic peptide

Having established the high reactivity of SEAE peptide thioesters, we next designed a microfluidic system featuring a three-stage process that includes (a) the generation of SEAE intermediates **5** from SEA peptide amides **3** or **4** in the first microfluidic module (stage 1 in Fig. 3), (b) the conversion of SEAE peptide thioester **5** into MPAA peptide thioester **2** followed by the intramolecular NCL in the second module (stage 2 in Fig. 3), and finally (c) a post-ligation treatment (stage 3 in Fig. 3). Figure 3 also details the various peptides examined in this study.

**Fig. 4:**
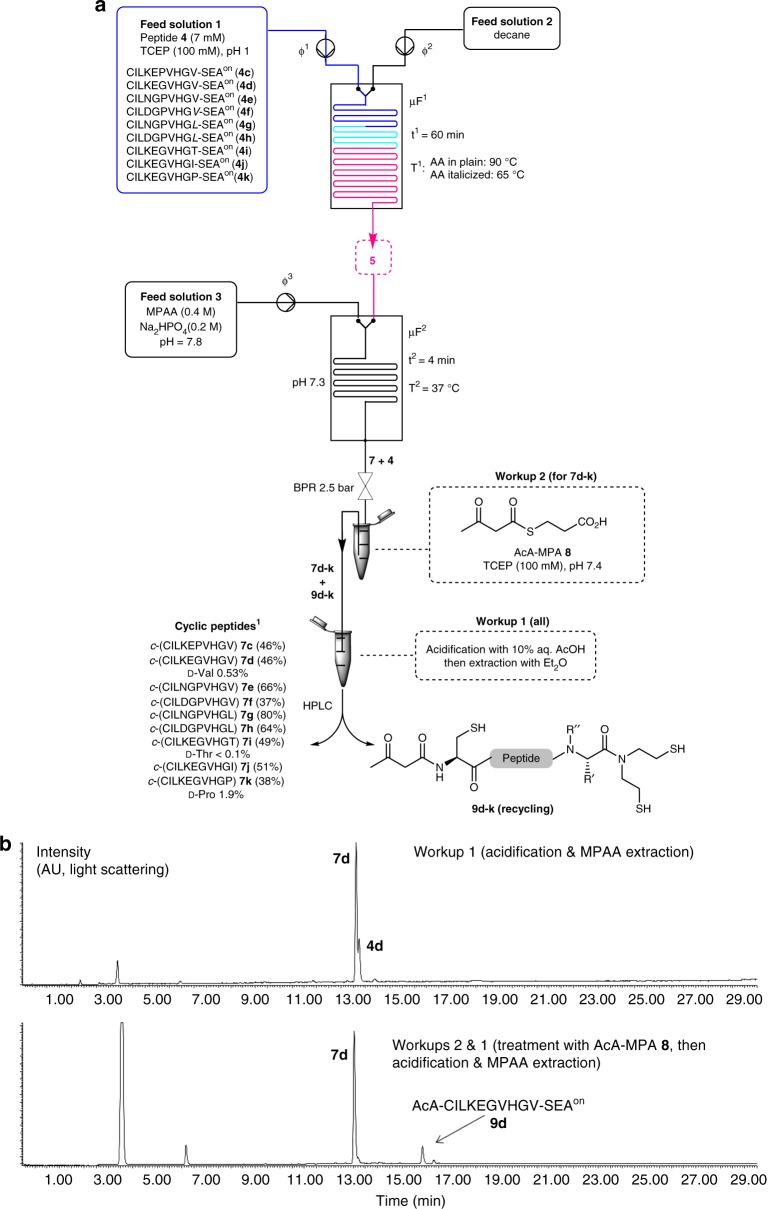
Optimized microfluidic setup. **a** Details of the optimized microfluidic system. The pH of the MPAA feed solution was set at 7.80, leading to an optimum internal pH of 7.3 within microfluidic element µF^2^, and the residence time t^2^ to 4 min. ϕ^1^ = ϕ^2^ = 3.3 µL min^−1^, ϕ^3^ = 35 µL min^−1^. The back pressure regulator (BPR) was set at 2.5 bars. The peptide concentration in the Feed solution 1 was 7 mM. For **7c**, the reactor effluent was quenched in 10% aqueous acetic acid. MPAA was then extracted with diethylether (5×) before HPLC analysis and purification (workup 1). For **7d-k**, the cyclic peptide co-eluted by HPLC with the starting SEA^on^ peptide **4**. Therefore, the reactor effluent was treated first with AcA-MPA **8**/TCEP dissolved in pH 7.4 phosphate buffer (workup 2) and then according to workup 1. ^1^ Isolated yields after HPLC purification. R′ and R′′ are for side-chain amino acids (see Fig. 1). **b** Representative HPLC chromatogram for cyclized peptide **7d** after workup 1 (top) or workup 2 and 1 (bottom)

The feasibility of the microfluidic strategy for cyclic peptide synthesis according to Fig. 3 was assessed starting with a reduced SEA^on^ peptide **4**. This allowed us to simplify the preliminary attempts by avoiding the reduction of disulfide **3** into dithiol **4**. The equilibrium between SEA^on^ amides **4** and SEAE peptides **5** considerably favors the amide at neutral pH to a point where SEAE peptides **5** are barely detectable by HPLC^39^. In contrast, the equilibrium favors the SEAE peptide **5** at pH 1 by ≥90% so that the activation was examined at this pH. Based upon preliminary data obtained in batch with model tetrapeptide TASV-SEA^on^ **4b** (Supplementary Fig. 2, Supplementary Methods), it turned out that the most balanced conditions for implementation in a microfluidic system required 90 °C and 60 min of reaction time (~90% conversion) for a difficult C-terminal amino acid such as Val. Pivotal adaptations for an implementation in flow were required since the reaction is sensitive to residence time distribution (Fig. 4a)^40,41^. The use of an immiscible carrier (decane, feed solution 2), as well as the insertion of a back pressure regulator (BPR) markedly improved the efficiency of the rearrangement process under microfluidic conditions with yields in the 81–94% range for peptides **7c,d** for example (Supplementary Fig. 12 and 39, Supplementary Table 6, Supplementary Methods).

**Fig. 5:**
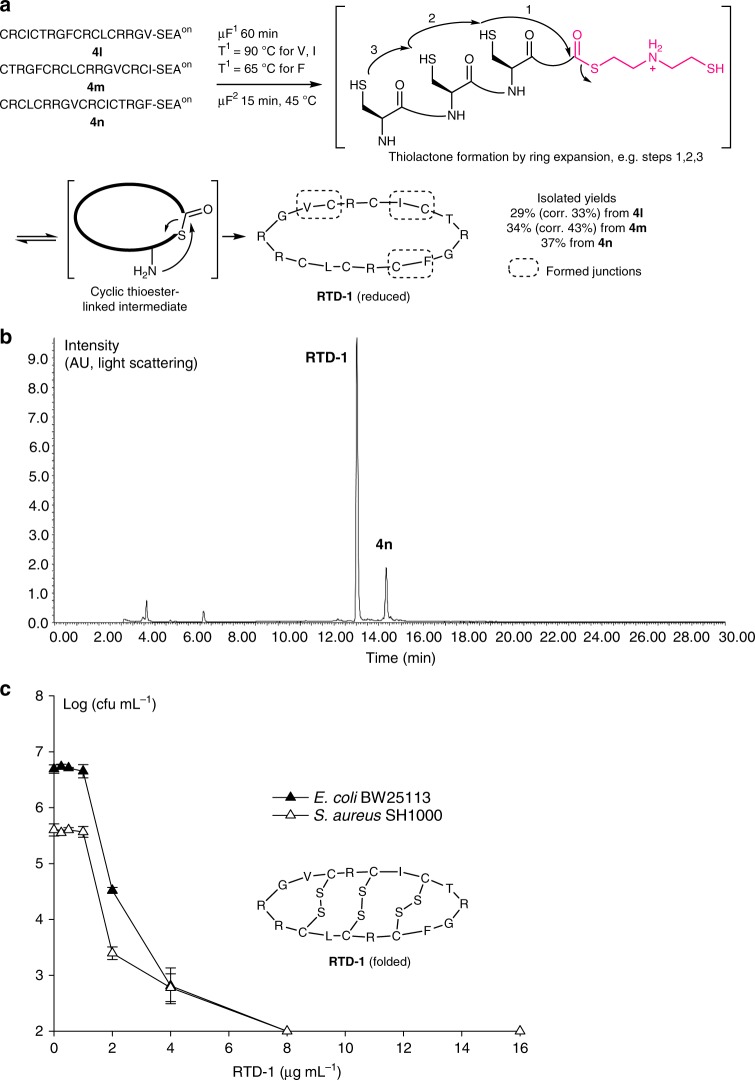
Expanding the strategy to antimicrobial peptide RTD-1. **a** Three different junctions were assessed for the synthesis of RTD-1. The formation of intermediate thiolactone species imposed some adaptation to the optimal conditions defined in Fig. 4a: the residence time and temperature in µF^2^ were increased from 4 to 15 min and from 37 to 45 °C. **b** Representative HPLC chromatogram for RTD-1 synthesis from peptide **4n**. **c** Antibacterial activity of folded RTD-1 produced from **4m** against *E. coli* BW 25113 (filled triangles) and *S. aureus* SH 1000 strains (open triangles). The experiment was done in triplicate. The data correspond to the mean and standard deviation of the log10 transformed colony-forming units (cfu) per mL. The limit of detection is 100 cfu mL^−1^

We also demonstrated that the microfluidic system can be fed with SEA^off^ peptides of type **3** as well. Batch experiments using tetrapeptide TASV-SEA^off^ **3b** established that SEA^off^ peptides of type **3** are refractory to reduction by *tris*(2-carboxyethyl)phosphine (TCEP) at pH 1 at room temperature, while reduction proceeds rapidly at 90 °C (Supplementary Fig. 3, Supplementary Methods). Therefore, when SEA^off^ peptides **3** are used in the feed solution 1, both reduction (**3**→**4**) and activation (**4**→**5**) steps proceed in the first microreactor (µF^1^, Supplementary Methods). Both types of precursors are easily accessible through 9-fluorenylmethyloxycarbonyl (Fmoc) SPPS (Supplementary Methods)^42^. Finally, we established that the temperature in µF^1^ could be lowered to 65 °C for the rearrangement of peptides terminated by non-problematic amino acids such as leucine (Leu, L; Fig. 4a; Supplementary Fig. 10 and 11), tyrosine (Tyr, Y; Supplementary Fig. 82) or phenylalanine (Phe, F; Fig. 5).

We next focused our efforts towards the SEAE-MPAA exchange reaction and the cyclization process under microfluidic conditions (stage 2 in Fig. 3, Fig. 4a). The first experiments were carried out using HIV polymerase-derived model peptide **4c**. Its cyclization toward *c*-(CILKEPVHGV) **7c** thus comes with a difficult junction, i.e., Val-Cys. Following the activation step, the acidic SEAE peptide thioester **5c** solution must be quickly mixed with the exogenous arylthiol MPAA. Since the pH in the second microfluidic module determines the kinetics of NCL^43^, as well as those of the undesirable deactivation of **5c** into **4c**, it was mandatory to identify the optimal pH for this step. pH optimization was realized with a dedicated system having two separate feeds of MPAA and sodium hydroxide after the activation module µF^1^, the relative flow rate of which enabled tuning the pH in the microfluidic element µF^2^ from 7.2 to 8.2 (Supplementary Methods). This study showed that conversion reached a maximum at pH ~7.3 and decreased almost linearly upon pH increase (Supplementary Fig. 23). At pH > 7.3 the fraction of amide **4c** in the mixture gradually increased, thus decreasing the yield of cyclic peptide **7c**. This study demonstrated the paramount importance of the precise control of the reaction conditions for such ligations. Subsequently, the two feeds of NaOH and MPAA were replaced by a single feed of MPAA giving the optimal pH of 7.3 in µF^2^ after mixing with the effluent from µF^1^ (Fig. 4a).

### Kinetic study and suppression of dimeric species

A kinetic study of the process was next carried out by adjusting the internal volume of module µF^2^ using the optimized pH conditions to screen residence times from 30 min down to 1 min. This experiment revealed that the reaction time had very little impact on the production of *c*-(CILKEPVHGV) **7c**, except for the shortest residence time of 1 min for which the reaction did not reach completion (Supplementary Fig. 24, Supplementary Methods). Most notably, reaction completion was observed within 2 min of residence time. In further experiments, a safety margin was included and the residence time was set at 4 min. Dimeric linear or cyclic species were also identified in the effluent at this occasion (Supplementary Fig. 24 and 25, Supplementary Methods). A two-fold dilution of the feed solution of peptide **4c** sufficed to suppress their formation. This updated setup allowed the preparative synthesis of cyclic peptide **7c** with an isolated yield of 46% following addition of an acetic acid quenching solution, MPAA extraction and HPLC purification (Fig. 4a, workup 1, Supplementary Methods).

### Advantages of SEAE chemistry under microfluidic conditions

With an optimal microfluidic system in hand, we sought to examine the advantage of using SEAE peptide thioesters in comparison with classical MPA or MPAA peptide thioesters **1** or **2** for peptide cyclization under microfluidic conditions. We also evaluated the benefit of performing the cyclization under microfluidic conditions over classical batch procedures.

The impact of the type of acyl donor on the efficiency of the cyclization process was studied by telescoping MPA peptide thioester CILKEPVHGV-MPA **1c** (Supplementary Fig. 18, Supplementary Methods) or MPAA peptide thioester CILKEPVHGV-MPAA **2c** (Supplementary Fig. 18 and 27, Supplementary Methods) dissolved at pH 1 with the MPAA feed solution 3. LC–MS analysis of the effluent after 4 min of residence time showed a poor conversion of MPA peptide thioester CILKEPVHGV-MPA **1c** into cyclic product **7c** (11%, Supplementary Fig. 26). Moreover, peptide **7c** was contaminated by some MPAA peptide thioester **2c** and dimeric species. Comparatively, the reaction with MPAA peptide thioester **2c** was more advanced, yet still incomplete, and the formation of some dimeric species was also observed in this case (Supplementary Fig. 28). Although MPAA was found to be essential for the cyclization process (Supplementary Fig. 4, Supplementary Methods), the latter experiment showed that the use of preformed MPAA peptide thioester **2c** could not reproduce similar high performances for reaction rate and purity profile as obtained when the microfluidic system was fed with SEA^on^ peptide **4c**. This suggests that part of the cyclic peptide **7c** is produced by the direct cyclization of SEAE peptide **5c** (Fig. 2). Therefore, the fast cyclization rate is due to the combined high reactivity of SEAE and MPAA peptide thioesters.

Next, peptide **4c** was also used to compare the assets of the microfluidic protocol versus a conventional batch procedure. Operation at steady state for over 3 h with the optimized microfluidic setup furnished consistent results with a homogenous purity profile for cyclic peptide **7c**, thus demonstrating the scale-independent nature of the process, as well as its stability (Supplementary Fig. 29, Supplementary Methods). In contrast, batch experiments on three different scales (10-, 25-, and 50-mg-production scales) provided inconsistent results with higher impurity contents (mainly unreacted peptide thioester and dimeric species, Supplementary Fig. 6 and 7, Supplementary Methods) than the corresponding microfluidic experiments.

Taken altogether, these experiments show that while the high reactivity of SEAE peptide thioesters has a major contribution to the rate of cyclization, this proceeds even faster under microfluidic conditions and with a higher purity profile compared to classical batch procedures.

### Exploring the peptide sequence dependency

We next examined the scope of the microfluidic process by varying some internal residues, the C-terminal amino acid bearing the SEAE functionality, and peptide length. Concerning the role of internal residues, several studies have shown that a proline can favor the backbone cyclization of protected peptides in organic solvents by classical activation procedures^44^. Indeed, the cis conformation of the peptide bond to proline is significantly more frequent (typically ~9% for Glu-Pro as in peptide **4c**) than for the other amino acids (<0.1%)^45^, thereby introducing a kink in the peptide chain and bringing the reactive ends closer in space. To evaluate the role of Pro6 on the cyclization of peptide **4c**, the microfluidic system was fed with analog **4d**, in which Pro6 was changed to Gly. The activation of **4d** into **5d** proceeded with a conversion of 94% in module µF^1^, comparable to the results obtained with the original model **4c**. The intramolecular cyclization module µF^2^ was telescoped to µF^1^, but co-elution of cyclic peptide **7d** and the starting SEA^on^ peptide **4d** precluded direct determination of an HPLC conversion. To solve this, the reactor effluent was treated with an excess of 3-((3-oxobutanoyl)thiol)propanoic acid (AcA-MPA **8**^46^) that reacted quantitatively with any remaining peptide **4d** by NCL at its N-terminal Cys. The resulting AcA peptide **9d** was easily separated from the cyclic peptide **7d** by HPLC, thus allowing both quantification and purification of cyclic peptide **7d** (Fig. 4b). Compared to the cyclization of **4c**, a higher conversion of *c*-(CILKEGVHGV) **7d** was measured (Supplementary Table 10, Supplementary Methods). Therefore and contrary to expectations^44^, in this case the presence of an internal proline in **4c** had a deleterious effect on the cyclization reaction.

We also examined the compatibility of the microfluidic system with internal asparaginyl-glycyl (NG) or aspartyl-glycyl (DG) dipeptide units, which are known to be particularly sensitive to several side-reactions such as aspartimide formation, deamidation, or peptide cleavage. Peptide **4e** equipped with an internal NG dipeptide unit and a C-terminal Val residue successfully yielded cyclic peptide **7e** with a higher yield than that obtained for **7c**. Some deamidation of asparagine residue was nevertheless observed during the activation step at 90 °C (~15–20%, Supplementary Fig. 8, Supplementary Methods). Comparatively, Leu analog **4g** was activated at 65 °C and cyclized without side-reactions (Supplementary Fig. 10). In contrast, DG dipeptide in peptide **4f** unit suffered as expected from a partial cleavage during the activation at 90 °C (Supplementary Fig. 9A). However, and here again, the side-reactions were nearly suppressed by performing the activation at 65 °C in the first microfluidic module (Supplementary Fig. 9B). Therefore, the activation of DG peptides **4f** and **4h** terminated by Val and Leu residues, respectively, was thus performed at 65 °C, albeit the activation was less advanced in the former case.

We next examined the influence of the C-terminal residue, which dictates the reactivity of peptide thioesters in NCL^18^. C-terminal Thr, Val, and Ile are sterically demanding and considered as difficult amino acids. Proline is even more problematic, and often considered as intractable^47,48^. In addition to slow kinetics, NCL reactions with peptidyl prolyl thioesters are also prone to side-reactions via deletion of two amino acids, i.e., the proline and the preceding residue, in the ligated product^49,50^. This side-reaction is particularly pronounced when the C-terminal Pro is preceded by Gly.

Peptides presenting a C-terminal Thr (**4i**) or Ile (**4j**) were successfully cyclized into **7i** and **7j**, respectively, through the fully concatenated setup and behaved similarly to peptides **4c,d** (Fig. 4a). For the Pro analog **4k**, the activation into **5k** was less efficient (66%). However, the intramolecular ligation step proceeded with high efficiency within 4 min of residence time, highlighting again the spectacular reactivity of SEAE thioesters in NCL. To rule out the possibility that fast ligation kinetics are simply a consequence of the intramolecular nature of the reaction, a control experiment involving ILKEPVHGP-SEA^on^ **4q** in Feed solution 1 and peptide CILKEPVHGV-NH_2_ **10** in MPAA Feed solution 3 was carried out (Supplementary Table 13, Supplementary Methods). The concatenated process was successfully operated using similar ligation conditions, and the peptide product ILKEPVHGP-CILKEPVHGV-NH_2_ **11** was isolated with 21% yield (28% corr.) after HPLC purification within a few minutes of ligation time, confirming the spectacular reactivity of SEAE peptides in NCL.

Note that due to its sequence, peptide **4k** is potentially prone to a deletion side-reaction. The deletion side-product, i.e., *c*-(CILKEGVH) **12**, was indeed observed, but it accounted for less than 8% of the total, showcasing the performance of the microfluidic system for backbone cyclization of problematic peptides. The slightly lower yield (38%) observed for **7k** arose from issues in separating the target peptide from the deletion side-product. No significant racemization was observed in the final products (Fig. 4a). Note that peptides **9d-k** were isolated as well and could potentially be recycled due to the easy removal of the AcA group with hydroxylamine at pH 4.

### Expanding the strategy to antimicrobial peptide RTD-1

We next aimed at the preparation of a larger and biologically relevant cyclic peptide, namely the 18 amino acid antimicrobial peptide RTD-1 (Fig. 5a)^51^, which was produced from three different precursors (**4l-n**), two of them involving the formation of a difficult junction (**4l**: Val-Cys; **4m**: Ile-Cys). Solubility issues in the microfluidic element µF^2^ required the addition of a denaturing agent (Gn·HCl, 6 M). We started with peptide SEA^on^ **4l**, which was subjected to the complete microfluidic assembly using optimized conditions identified above for difficult junctions (Fig. 4a, Supplementary Table 9, Supplementary Methods). LC–MS analysis of the crude product revealed the formation of RTD-1 in its reduced form (49%), as expected, but also a large proportion (34%) of side-products having the same mass as RTD-1. We hypothesized that the side-products were macrocyclic thiolactone species, the formation of which most likely involves a ring expansion mechanism as discussed by Tam and coworkers (Fig. 5a)^52^. To test this hypothesis, RTD-1 analog **4o**, in which all the Cys residues except the N*-*terminal one were changed to Ala, was prepared (Fig. 2). The microfluidic experiment with peptide **4o** furnished cyclic peptide *c*-(CRAIATRGFARALARRGV) **7o** in good isolated yield (HPLC yield 71%, isolated 37%) without side-product formation. This suggests that the rearrangement of the thiolactones formed by proximity-driven attack of internal cysteine thiols on the C-terminal thioester, which ultimately yields the backbone cyclized peptide by ring expansion, is rate-limiting upon cyclization to RTD-1 under microfluidic conditions. To promote the rearrangement of the thiolactone species, we increased both the residence time (from 4 to 15 min) and the temperature in module µF^2^ (from 37 to 45 °C). These microfluidic conditions significantly reduced the occurrence (<10%) of thiolactone species and increased the conversion of **4l** into RTD-1 (71% HPLC yield, isolated yield 29%) accordingly. These conditions were next successfully applied to peptide **4m** (difficult Ile-Cys junction). In the case of RTD-1 precursor **4n**, which has a non-problematic C-terminal Phe residue, the activation in µF^1^ module was performed at 65 °C (Fig. 5b). Cyclization in module µF^2^ proved to be highly efficient although some epimerisation of the Phe residue was noticed (4.9%). No thiolactone intermediates remained in the crude reactor effluent in this case. The higher accessibility of the carbonyl group of Phe to thiol nucleophiles compared to Val or Ile most likely allows a faster rearrangement of intermediate thiolactone species. This example shows that the microfluidic system can be easily tailored to the specific requirements of a variety of junctions.

Native RTD-1 peptide is stabilized by three disulfide bonds, which are important for the cyclic peptide to exhibit its full biological activity. The reduced RTD-1 peptide is known to spontaneously form the native pattern of disulfide bonds upon oxidative folding^51^. Therefore, RTD-1 peptide produced from peptide **4l** was oxidized (Supplementary Methods)^51^, and tested for its antibacterial activity against *E. coli* and *S. aureus* strains (Fig. 5c, Supplementary Methods). The folded RTD-1 peptide displayed the expected antibacterial activity in this assay, showing the capacity of the microfluidic system to produce a cyclic peptide of biological interest.

As a final demonstration for this study, we performed the cyclization of a longer peptide model sequence (28 mer, peptide **4p**) derived from the hepatocyte growth factor (Fig. 2). Since this peptide features a non-problematic C-terminal Tyr residue, cyclization was performed using the microfluidic setup defined for RTD-1 precursor **4n** (see Fig. 5a and Supplementary Fig. 82). Cyclization proceeded efficiently by providing the target cyclic peptide **7p** with a 41% yield after HPLC purification.

## Discussion

The design of highly reactive peptidyl donors and extremely fast ligation methods is a timely and significant goal. Such chemical tools can streamline the production of complex peptides and especially those implicating the formation of difficult junctions. Importantly, fast ligation methods potentially open the way toward peptide production under microfluidic conditions. However, to benefit from all the well-established assets of continuous flow operation, the issues related to using and controlling highly reactive peptidyl donors must be overcome. Ideally, such reactive species should be generated in the microfluidic system to avoid their isolation, storage, and handling. In this regard, the microfluidic NCL procedure reported herein constitutes a significant advance for peptide chemical ligation under homogeneous conditions by providing a straightforward and operationally simple method to exploit the enhanced reactivity of SEAE peptidyl thioesters at their fullest. The procedure relies on a highly modular microfluidic system that enables progressive activation starting from stable and readily available peptide amides. The highly integrated nature of the microfluidic system greatly simplifies peptide cyclization in a scale-independent and automated manner. Several production campaigns sustained the production of high-quality materials with only small variations. Attempts to reproduce similar performance with a batch setup provided inconsistent results with higher impurity contents.

Short cyclative ligation rates (<4 min) were obtained even for difficult (Val, Ile, and Thr) and the most intractable (Pro) junctions. The high reactivity of SEAE peptidyl thioesters cannot be deduced from previous studies that involved simple thioester functionalities^38^. In addition, reports dealing with the impact of the thiol backbone on the reactivity of the corresponding thioester remain scarce^53,54^. Recently, a few studies unveiled the positive impact of non-proteinogenic C-terminal thiol amino acids on thioester reactivity^18,55,56^. Our approach is particularly appealing since it does not require the modification of the peptide structure and post-ligation chemical transformations. It relies on robust chemistry amenable to conventional SPPS for the preparation of the starting SEA peptide fragments. The high reactivity of SEAE peptidyl thioesters should stimulate further work with the objective of understanding how chemical groups internal to the thioester peptide can dramatically boost its reactivity.

In summary, the chemistry and microfluidic system described in this study are flexible and versatile, and could be easily adapted to the inherent specificities of various cyclic peptides. This work provides the preliminary step towards the development of a generalized microfluidic procedure for the scale-independent production of cyclic peptide architectures, as well as post-ligation cosmetic operations (desulfurization, oxidative folding). The procedure makes peptide cyclization much more accessible, particularly for preparative applications.

## Methods

### Reagents

2-(1*H*-Benzotriazol-1-yl)-1,1,3,3-tetramethyluronium fluorophosphate (HBTU) and *N*-Fmoc protected amino acids were obtained from Iris Biotech GmbH. 4-Mercaptophenylacetic acid (97%, MPAA), 3-mercaptopropionic acid (MPA), *tris*(2-carboxyethyl)phosphine hydrochloride (≥98%, TCEP), thiophenol, triisopropylsilane (TIS), guanidine hydrochloride (≥99%), sodium phosphate dibasic dihydrate (≥99%), hydrochloric acid (reagent grade, 37%), and sodium hydroxide (pellets, 97%) were purchased from Sigma-Aldrich. All other reagents were purchased from Acros Organics or Merck and were of the purest grade available. Peptide synthesis grade *N,N*-dimethylformamide, dichloromethane, diethylether, acetonitrile, heptane, LC–MS-grade acetonitrile (0.1% TFA), LC–MS-grade water (0.1% TFA), *N,N*-diisopropylethylamine (DIEA), and acetic anhydride were purchased from Biosolve and Fisher-Chemical. Trifluoroacetic acid (TFA) was obtained from Biosolve. Decane (synthesis grade) was purchased from Merck. Solvents and reagents were used as received. Water was purified with a Milli-Q UltraPure Water Purification System.

### Peptide synthesis

SEA^on^ peptides **4c-p** were synthesized from SEA polystyrene resin using classical Fmoc SPPS protocols^37^. In brief, amino acids (10 equiv.) were activated using HBTU (9.5 equiv.)/DIEA (10 equiv) in DMF. The peptidyl resin was acetylated after each coupling step using acetic anhydride (10% by vol) and DIEA (5% by vol.) in DMF. The removal of the Fmoc group was performed by treating the peptidyl resin with piperidine (20% by vol.) in DMF. The peptides were deprotected and cleaved from the resin using TFA/water/TIS/thiophenol cocktail (92.5/2.5/2.5/2.5 by vol.). The crude SEA^on^ peptides **4c-k** were used without further purification. SEA^on^ peptides **4l-p** were purified by reversed-phase HPLC prior to cyclization.

### Microfluidic modules

Microfluidic modules were constructed with commercially available elements from IDEX/Upchurch Scientific, including high purity PFA capillaries, connectors, ferrules, static micromixers, and in-line check-valves (Supplementary Tables 1 and 2). High force syringe pumps (Nexus 6000) for feed delivery were purchased from Chemyx, and the dome-type BPR was purchased from Zaiput Flow Technologies (Supplementary Methods).

### Optimized microfluidic setup

The microfluidic ligation setup consisted of two microfluidic modules constructed from high purity PFA capillary (µF^1^ = 400 µL internal volume and µF^2^ = 170 µL internal volume) fluidically connected in series (Supplementary Fig. 17, Supplementary Methods). The temperature of µF^1^ was set at 90 °C or 65 °C, and µF^2^ was operated at 37 °C or 45 °C (Supplementary Table 8 and 9). The ligation setup was operated with a counter-pressure of 2.5 bar. Static mixers were utilized upstream module µF^1^ for establishing the segmented regime prior to the activation step and upstream µF^2^ for the injection of the MPAA feed solution for the ligation step (Supplementary Fig. 12 and 17, Supplementary Methods).

### Typical microfluidic ligation procedure

The first module (µF^1^) was concomitantly fed with a solution of the desired starting SEA^on^ peptide **4** (pH 1) and *n*-decane (flow rate = 3.3 µL min^−1^ for each). The effluent of µF^1^ was then mixed with a solution of MPPA (flow rate = 35 µL min^−1^, pH 7.8), and the resulting reaction mixture (pH 7.3) was conveyed to the second module (µF^2^). Details of the procedures can be found in Supplementary Methods. The reactor effluent was collected at steady state, and processed. Specific downstream purification procedures including post-ligation procedures, MPAA extraction, and HPLC purification were established for each peptide sequence.

### Typical post-ligation procedures

Workup 1 was used for peptides **7c**, **7l-p** (Supplementary Methods). The reactor effluent was collected in a 10% aqueous acetic acid solution. MPAA was extracted 5 times with diethylether. The aqueous solution was analyzed by HPLC or LC–MS and subsequently purified by HPLC.

Workups 2 and 1 were used for peptides **7d-k**. For workup 2 (Supplementary Methods), the reactor effluent was collected in a solution of AcA-MPA^46^ (**8**, 1.2 equiv.) and TCEP.HCl (100 mM) in sodium phosphate buffer (0.2 M, pH = 7.4), and maintained overnight at room temperature under a nitrogen atmosphere. The corresponding reaction mixture was next treated according to workup 1.

### Peptide purification

The peptides were purified by reversed-phase HPLC on a C18 column using a linear gradient of increasing concentration of eluent B in eluent A (eluent A: 0.1% by vol. of trifluoroacetic acid (TFA) in water; eluent B: 0.1% vol. of TFA in acetonitrile/water: 4/1 by vol., flow rate of 6 mL min^−1^, UV detection at 215 nm). The selected fractions were then combined, frozen, and lyophilized.

### Peptide characterization

The peptides were characterized by analytical LC–MS on a reversed-phase XBridge BEH300 C18 column (3.5 μm, 300 Å, 4.6 × 150 mm) at 30 °C using a linear gradient: 0–100% of eluent B in eluent A over 30 min at a flow rate of 1 mL min^−1^. The column eluate was monitored by UV at 215 nm, by evaporative light scattering or by electrospray ionization mass spectrometry (ESI-MS). MALDI-TOF mass spectra were recorded with a Bruker Autoflex Speed using alpha cyano 4-hydroxycinnaminic acid, sinapinic acid or 2,5-dihydroxybenzoic acid (DHB) as matrix.

### Kinetic studies

The aliquots (2 µL) were quenched with aqueous acetic acid (10% by vol., 100 µL). MPAA was removed by extracting the aqueous phase with diethylether (5 times). The samples were then analyzed by LC–MS using a C18XBridge column as described above in the Methods section: peptide characterization.

### RTD-1 antibacterial activity

*E. coli* BW25113 (kindly provided by Coli Genetic Stock Centre, CGSC), and *S. aureus* SH1000 (kindly provided by Prof. S.J. Foster, University of Sheffield) were grown overnight in Cation-Adjusted Mueller Hinton II Broth (37 °C, 150 rpm)^51^. Cultures were then diluted 1 in 20 and grown for 2 h (37 °C, 150 rpm) to obtain a log phase bacterial culture. Log phase cultures were then pelleted (3000× *g*, 5 min) and washed twice in 10 mM PIPES (pH 7.4) with 5 mM glucose. The bacterial suspension was diluted in piperazine-*N*,*N*′-*bis*(2-ethanesulfonic acid) buffer (10 mM, pH 7.4) with 5 mM glucose to an optical density of 0.00125 at 600 nm. A volume of 150 µL of the bacterial suspensions were added to the wells of a 96-well plate. A volume of 3 µL of RTD-1 serially diluted in water (at 50 × final concentration, 800–25 µg mL^−1^) was then added to the bacteria to give a final concentration range of 16–0.5 µg mL^−1^. Bacteria viability was then determined by counting colony-forming units following plating on LB agar.

### Data availability

All relevant data are included in the manuscript and Supplementary Information. More data are available from the authors upon request.

## Electronic supplementary material

Supplementary Information
