# Supplementary material for: Accelerated microfluidic native chemical ligation at difficult amino acids toward cyclic peptides
Source: Nat Commun. 2018 Jul 20;9:2847. doi: 10.1038/s41467-018-05264-8 (PMC6054628; doi:10.1038/s41467-018-05264-8)
Supplement: Supplementary file 1 — Supplementary Information [file 41467_2018_5264_MOESM1_ESM.pdf]

Accelerated microfluidic native chemical ligation at difficult amino acids toward cyclic peptides

Ollivier et al.

Supplementary Figures

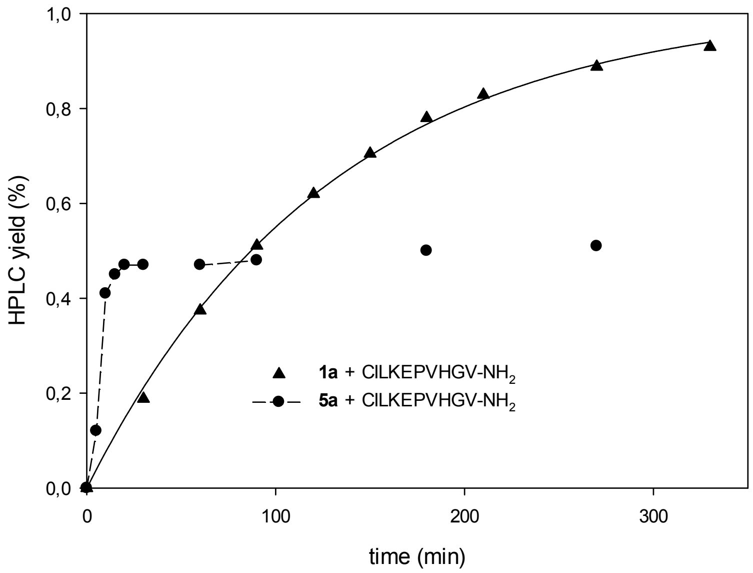

Supplementary Figure 1. HPLC yield for the ligation of SEAE peptide 5a or MPA peptide thioester 1a with Cys peptide CILKEPVHGV-NH_2 10. Synthesis of peptide ILKEPVHGV-CILKEPVHGV-NH_2 **14**.

A)

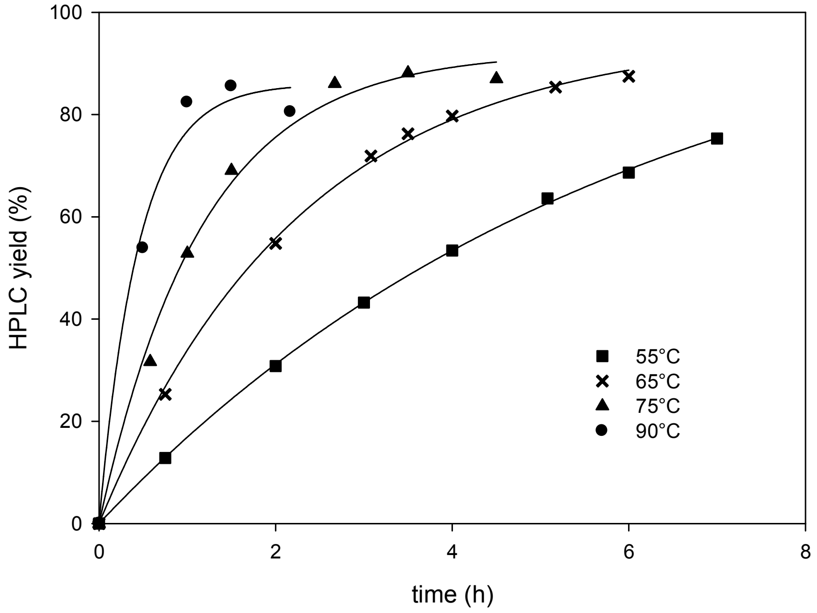

B)

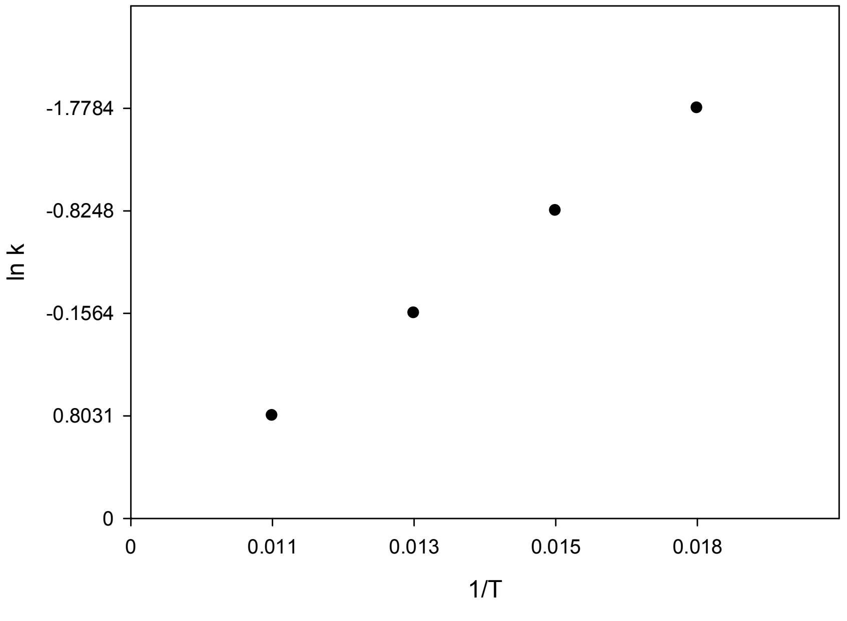

Supplementary Figure 2. Effect of the temperature on the rate of SEAE peptide thioester 5 formation. Rate of SEAE thioester peptide **5b** formation at different temperatures (55, 65, 75 and 90 °C). A) Rate; B) Arrhenius plot.

A)

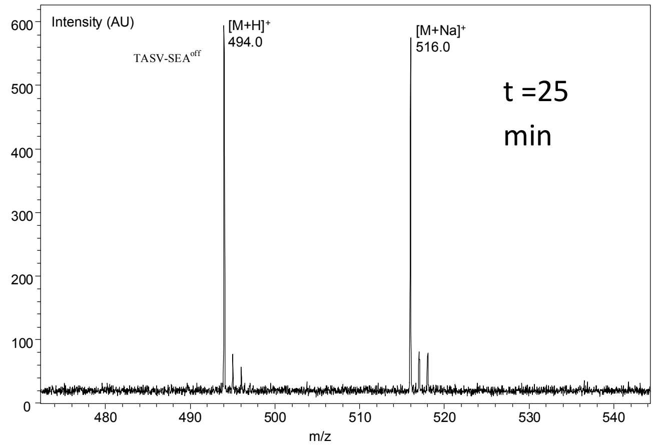

B)

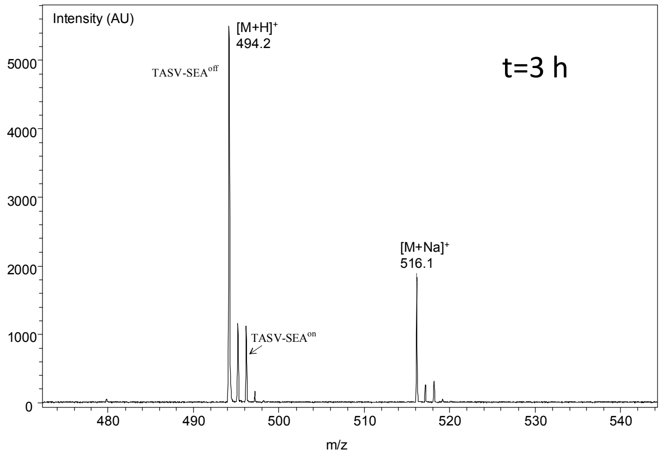

C)

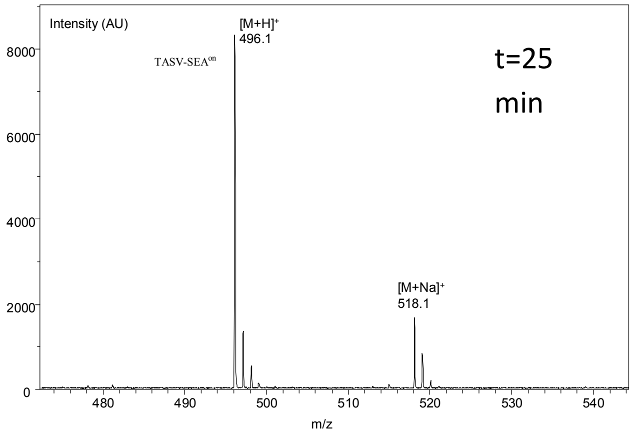

Supplementary Figure 3. Stability of SEA^{off} peptides of type 3 at pH 1 in the presence of TCEP at room temperature. MALDI-TOF analysis of peptide **3b**/TCEP solution at pH 1. A) At room temperature after 25 min; B) At room temperature after 3 h; C) At 90 °C after 25 min. Matrix 2,5-dihydroxybenzoic acid, positive detection mode, SEA^{off} peptide **3b** $[M+H]^+$ calcd. (monoisotopic) 494.2, found 494.2, SEA^{on} peptide **4b** $[M+H]^+$ calcd. (monoisotopic) 496.2, found 496.1.

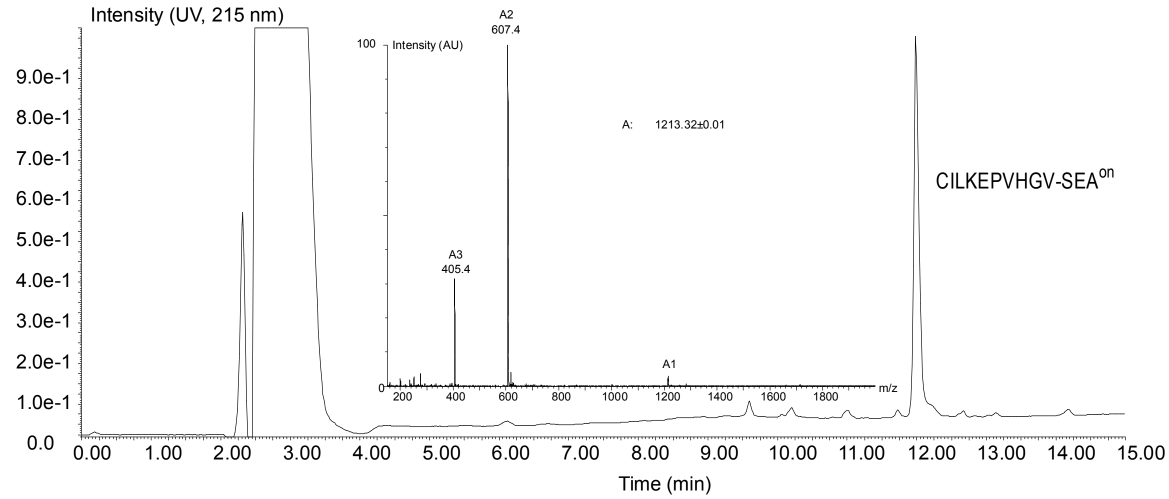

Supplementary Figure 4. Importance of MPAA for cyclization. LC-MS analysis at t=4 min. LC trace, eluent A 0.10% TFA in water, eluent B 0.10% TFA in CH_3CN/water: 4/1 by vol. C18 Xbridge BEH 300 Å 5 µm (4.6 × 250 mm) column, gradient 0-50% B in 15 min (1 mL min^{-1}, UV 215 nm). MS trace. $[M+2H]^{2+}$ m/z calcd. (monoisotopic) 607.3, obs 607.4, $[M+3H]^{3+}$ m/z calcd. (monoisotopic) 405.2, obs 405.4.

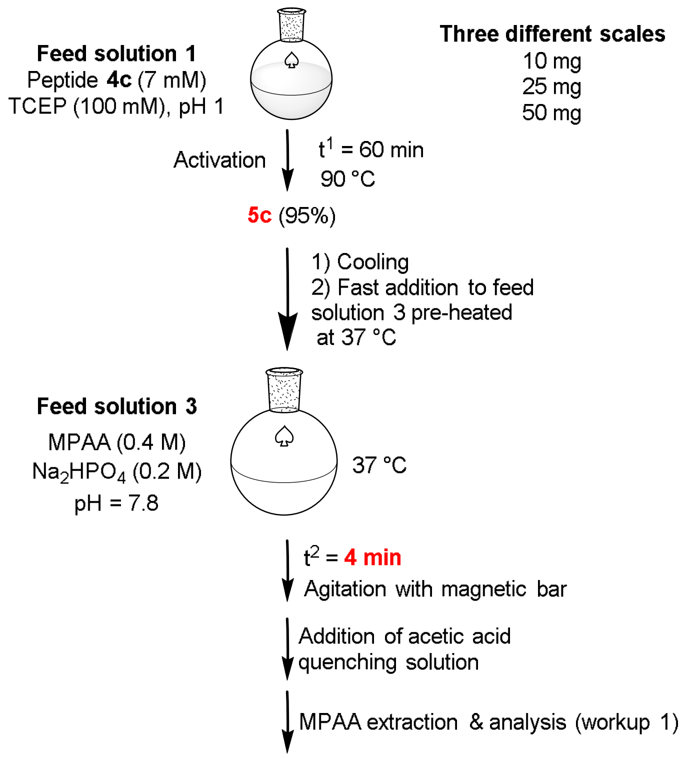

Supplementary Figure 5. Batch experiments for peptide cyclization with comparable operational conditions than for microfluidic operation using peptide 4c as model substrate.

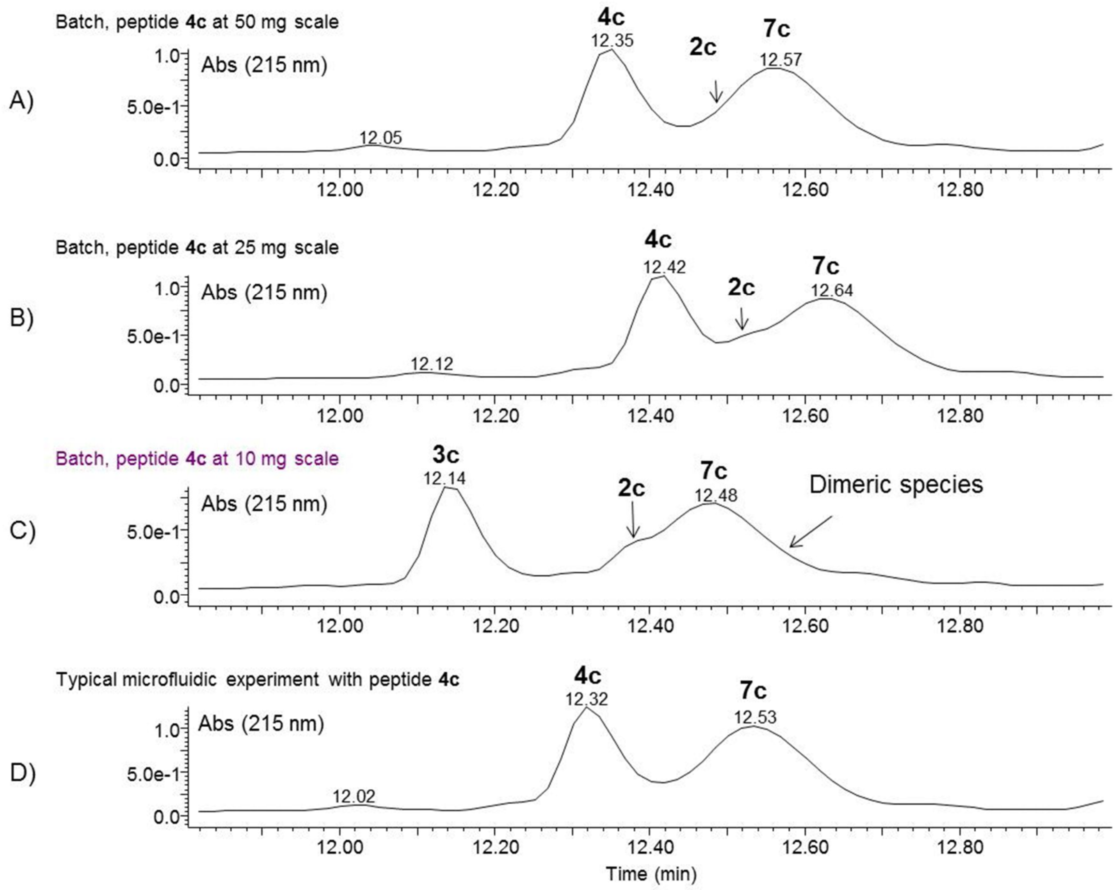

Supplementary Figure 6. LC-MS analysis of the batch experiments (A: 50 mg scale, B: 25 mg scale, C: 10 mg scale) for peptide 4c cyclization mimicking the process utilized under microfluidic conditions (D).

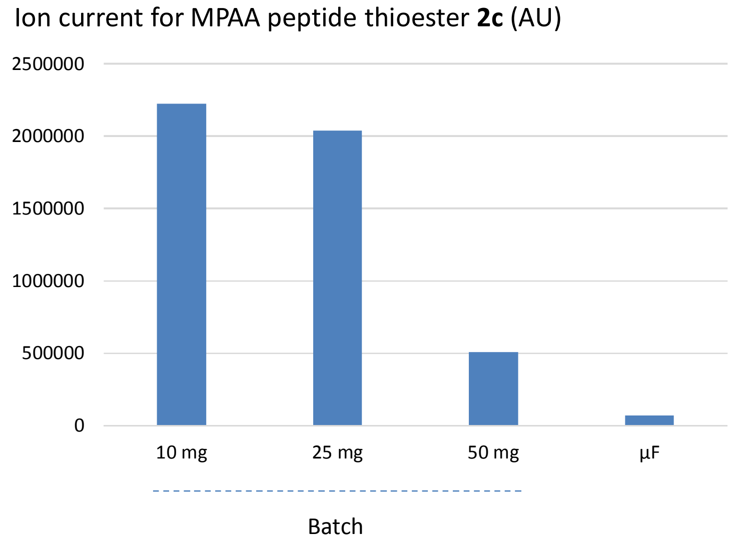

Supplementary Figure 7. LC-MS analysis of the batch experiments for peptide 4c cyclization mimicking the process utilized under microfluidic conditions (µF). Integration of the ion current for MPAA peptide thioester 2c (double charge species) for batch and microfluidic experiments. Each batch experiment was performed only once.

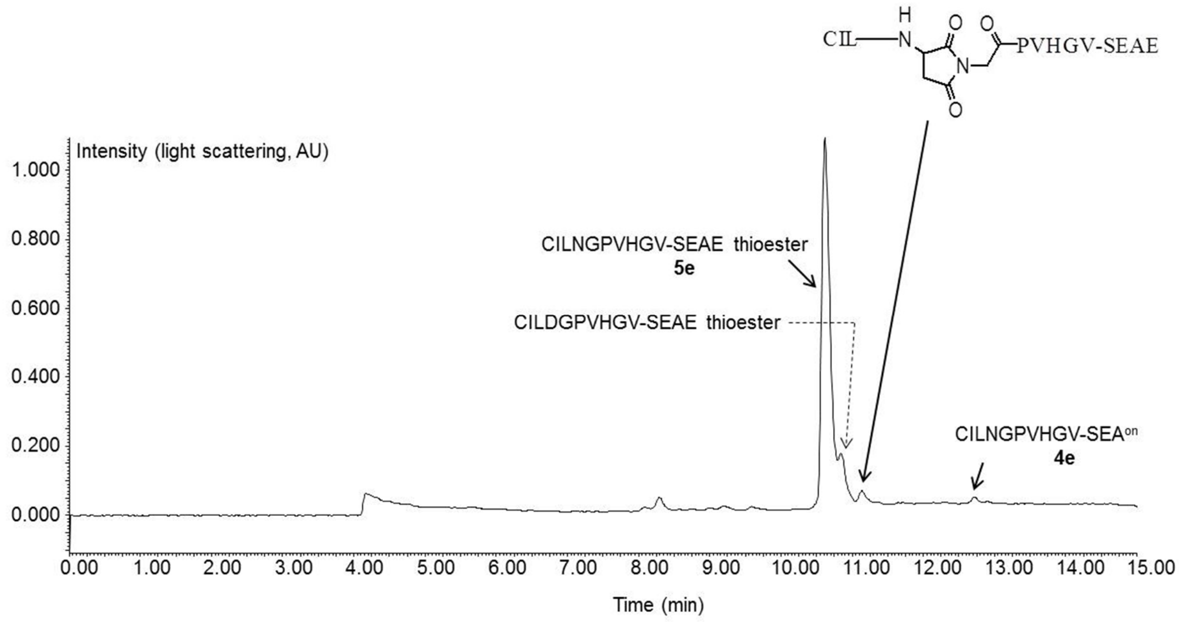

Supplementary Figure 8. Compatibility of Asn-Gly dipeptide unit with the activation step. Activation of **4e** at 90 °C. LC-MS analysis of peptide **5e**. LC trace, eluent A 0.10% TFA in water, eluent B 0.10% TFA in CH_3CN/water: 4/1 by vol. C18 Xbridge BEH 300 Å 5 µm (4.6 × 250 mm) column, gradient 0-50% B in 15 min (1 mL min^{-1}, detection light scattering).

A)

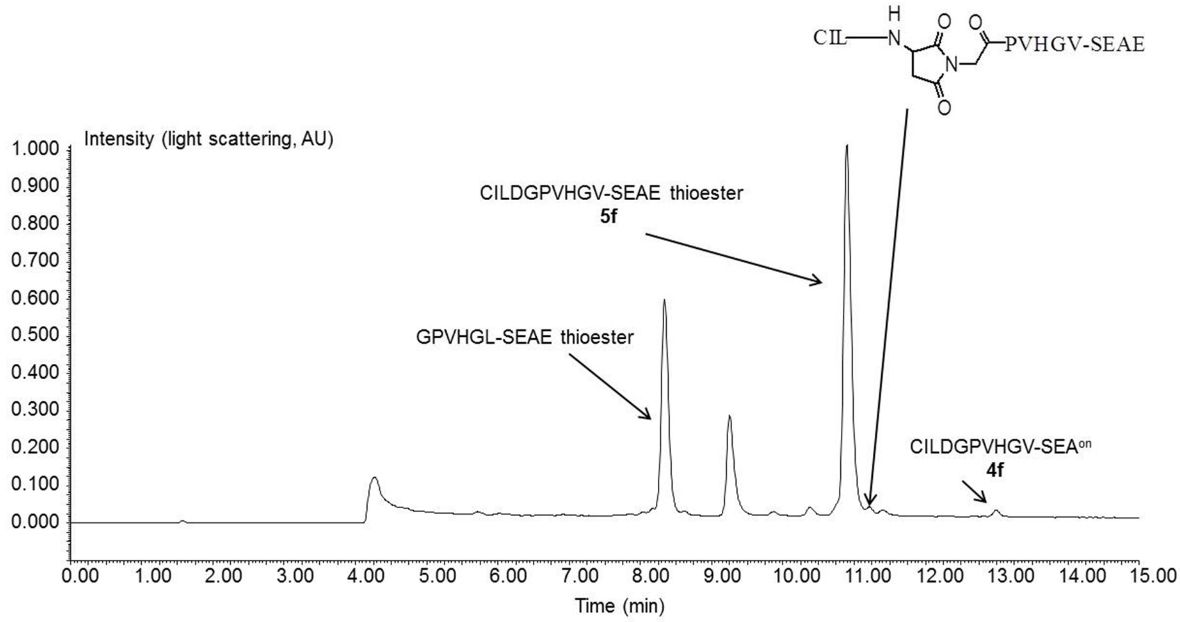

B)

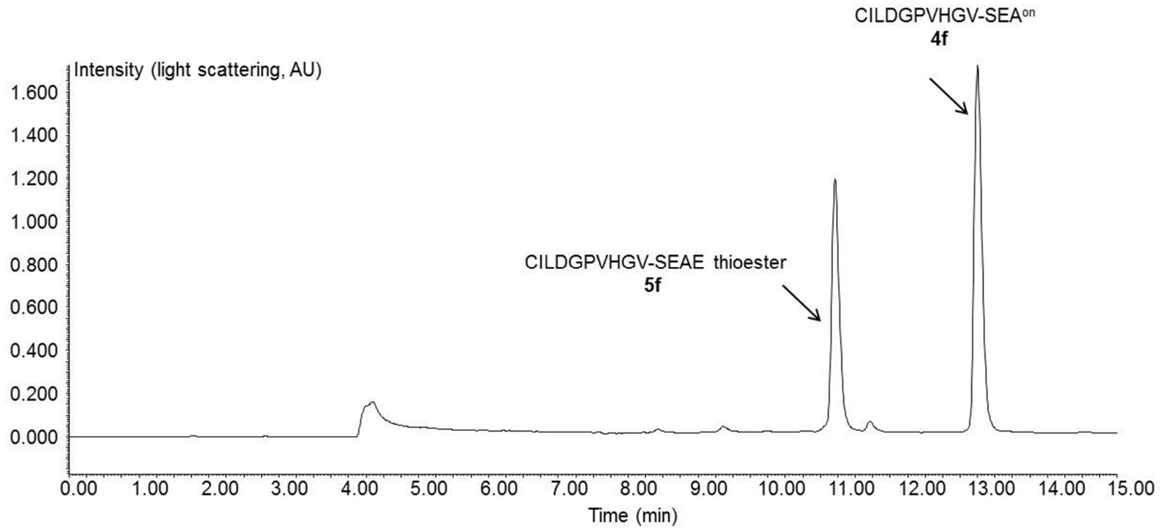

Supplementary Figure 9. Compatibility of Asp-Gly dipeptide units with the activation step. A) Activation of **4f** at 90 °C, B) Activation of **4f** at 65 °C. LC-MS analysis of peptide **5f**. A) Rearrangement at 90 °C. B) Rearrangement at 65 °C. LC trace, eluent A 0.10% TFA in water, eluent B 0.10% TFA in CH_3CN/water: 4/1 by vol. C18 Xbridge BEH 300 Å 5 µm (4.6 × 250 mm) column, gradient 0-50% B in 15 min (1 mL min^{-1}, detection light scattering).

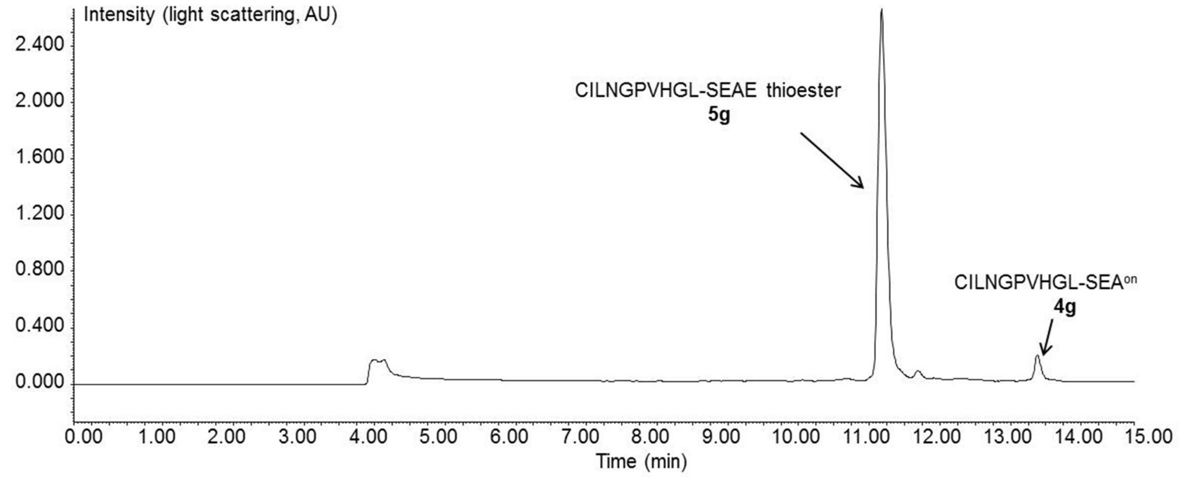

Supplementary Figure 10. Compatibility of Asn-Gly dipeptide unit with the activation step. Activation of **4g** at 65 °C. LC-MS analysis of peptide **5g**. LC trace, eluent A 0.10% TFA in water, eluent B 0.10% TFA in CH_3CN/water: 4/1 by vol. C18 Xbridge BEH 300 Å 5 µm (4.6 × 250 mm) column, gradient 0-50% B in 15 min (1 mL min^{-1}, detection light scattering).

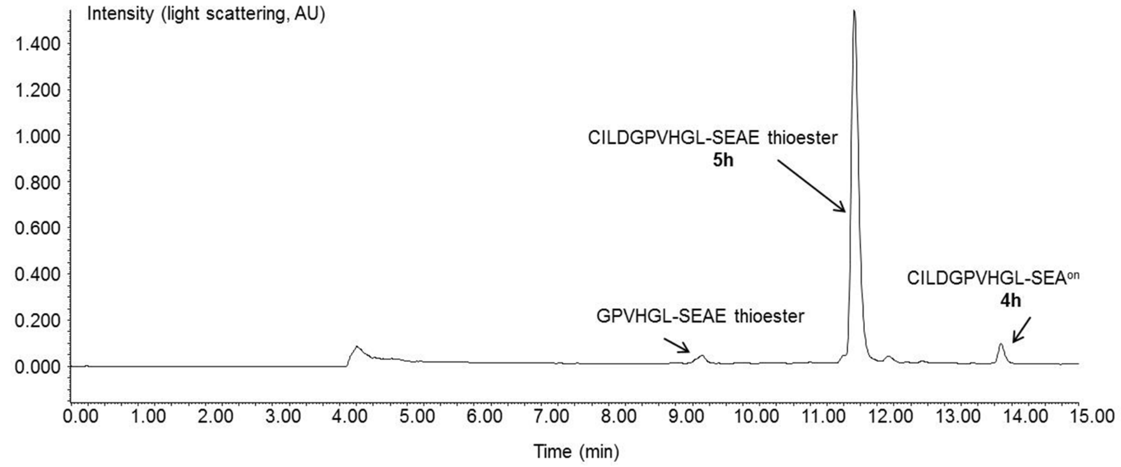

Supplementary Figure 11. Compatibility of Asp-Gly dipeptide units with the activation step. Activation of **4h** at 65 °C. LC-MS analysis of peptide **5h**. LC trace, eluent A 0.10% TFA in water, eluent B 0.10% TFA in CH_3CN/water: 4/1 by vol. C18 Xbridge BEH 300 Å 5 µm (4.6 × 250 mm) column, gradient 0-50% B in 15 min (1 mL min^{-1}, detection light scattering).

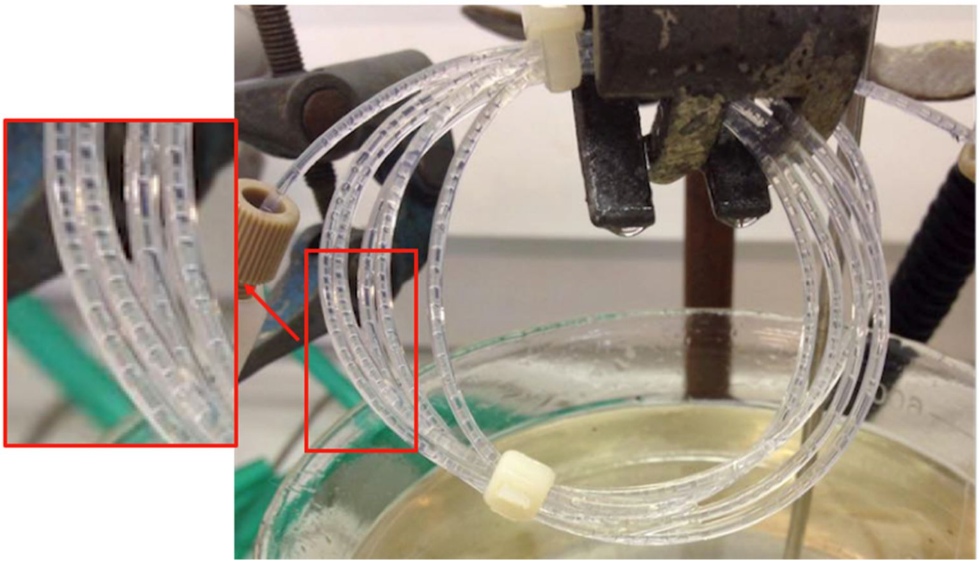

Supplementary Figure 12. Segmented regime obtained from the concomitant injection of Feed solution 1 and an immiscible carrier (decane) in µF[1].

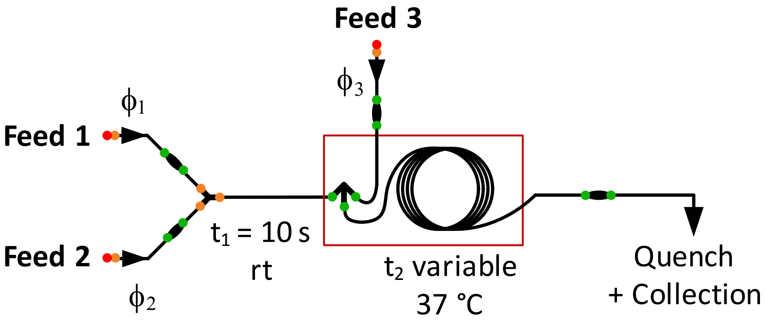

- Red ETFE female luer to female FB 1/4-28 quick connect adapter
- Super Flangeless Nuts, PEEK 1/4-28 thread for 1/16” OD tubing (+ ferrules)
- One-Piece Fingertight PEEK, 10-32 Coned, for 1/16" OD
- In-line check valve PEEK/perfluoroelastomer 1/4-28 thread for 1/16" OD tubing
- Y-mixer, natural PEEK 1/4-28 thread for 1/16" o.d. tubing, 0.02" through hole
- Static mixer, natural PEEK 1/4-28 thread for 1/16" o.d. tubing, 0.02" through hole
- Natural polypropylene standard low pressure union 1/4-28

Supplementary Figure 13. Microfluidic setup for the kinetic experiment with SEAE peptide thioester 5a or MPA peptide thioester 1a in reaction with MPAA. See also **Supplementary Table 1, Supplementary Table 2**.

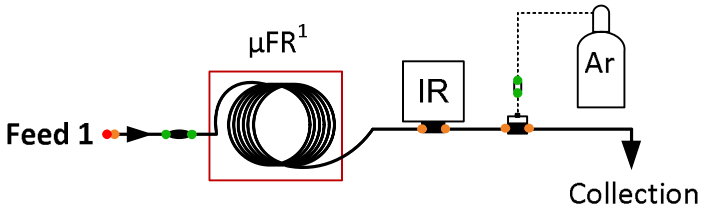

- Red ETFE female luer to female FB 1/4-28 quick connect adapter
- Super Flangeless Nuts, PEEK 1/4-28 thread for 1/16” OD tubing (+ ferrules)
- One-Piece Fingertight PEEK, 10-32 Coned, for 1/16" OD
- In-line check valve PEEK/perfluoroelastomer 1/4-28 thread for 1/16" OD tubing
- Y-mixer, natural PEEK 1/4-28 thread for 1/16" o.d. tubing, 0.02" through hole
- Back-pressure regulator
- Natural polypropylene standard low pressure union 1/4-28

Supplementary Figure 14. Microfluidic setup for the identification of the gaseous product formed during the activation step.

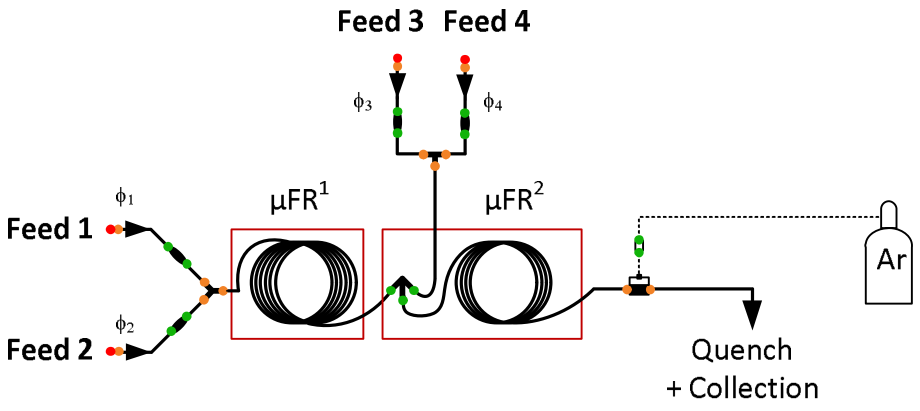

- Red ETFE female luer to female FB 1/4-28 quick connect adapter
- Super Flangeless Nuts, PEEK 1/4-28 thread for 1/16” OD tubing (+ ferrules)
- One-Piece Fingertight PEEK, 10-32 Coned, for 1/16" OD
- In-line check valve PEEK/perfluoroelastomer 1/4-28 thread for 1/16" OD tubing
- Y-mixer, natural PEEK 1/4-28 thread for 1/16" o.d. tubing, 0.02" through hole
- T-mixer, natural PEEK 1/4-28 thread for 1/16" o.d. tubing, 0.02" through hole
- Static mixer, natural PEEK 1/4-28 thread for 1/16" o.d. tubing, 0.02" through hole
- Back-pressure regulator
- Natural polypropylene standard low pressure union 1/4-28

Supplementary Figure 15. Microfluidic setup for pH optimization.

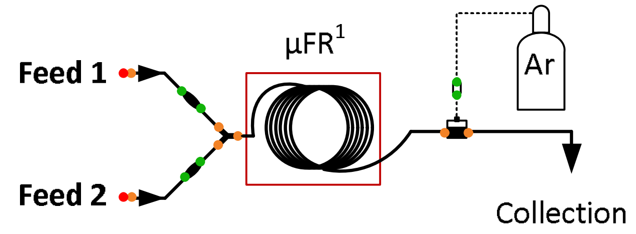

- Red ETFE female luer to female FB 1/4-28 quick connect adapter
- Super Flangeless Nuts, PEEK 1/4-28 thread for 1/16” OD tubing (+ ferrules)
- One-Piece Fingertight PEEK, 10-32 Coned, for 1/16" OD
- In-line check valve PEEK/perfluoroelastomer 1/4-28 thread for 1/16" OD tubing
- Y-mixer, natural PEEK 1/4-28 thread for 1/16" o.d. tubing, 0.02" through hole
- Back-pressure regulator
- Natural polypropylene standard low pressure union 1/4-28

Supplementary Figure 16. General microfluidic setup for the synthesis of SEAE peptide thioesters 5 from SEA$^{off/on}$ peptides 3 or 4.

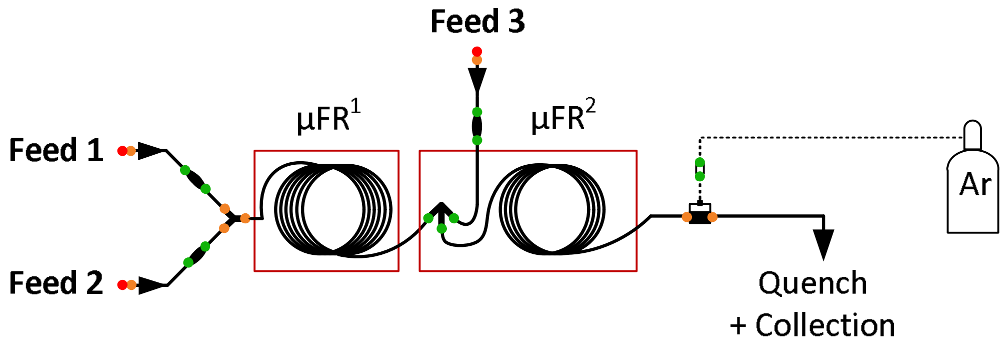

- Red ETFE female luer to female FB 1/4-28 quick connect adapter
- Super Flangeless Nuts, PEEK 1/4-28 thread for 1/16” OD tubing (+ ferrules)
- One-Piece Fingertight PEEK, 10-32 Coned, for 1/16" OD
- In-line check valve PEEK/perfluoroelastomer 1/4-28 thread for 1/16" OD tubing
- Y-mixer, natural PEEK 1/4-28 thread for 1/16" o.d. tubing, 0.02" through hole
- Static mixer, natural PEEK 1/4-28 thread for 1/16" o.d. tubing, 0.02" through hole
- Back-pressure regulator
- Natural polypropylene standard low pressure union 1/4-28

Supplementary Figure 17. General microfluidic setup for the fully telescoped sequence: SEAE peptide thioester 5 formation and intramolecular ligation toward 7 or RTD-1 peptide. See **Supplementary Table 8** and **Supplementary Table 9**.

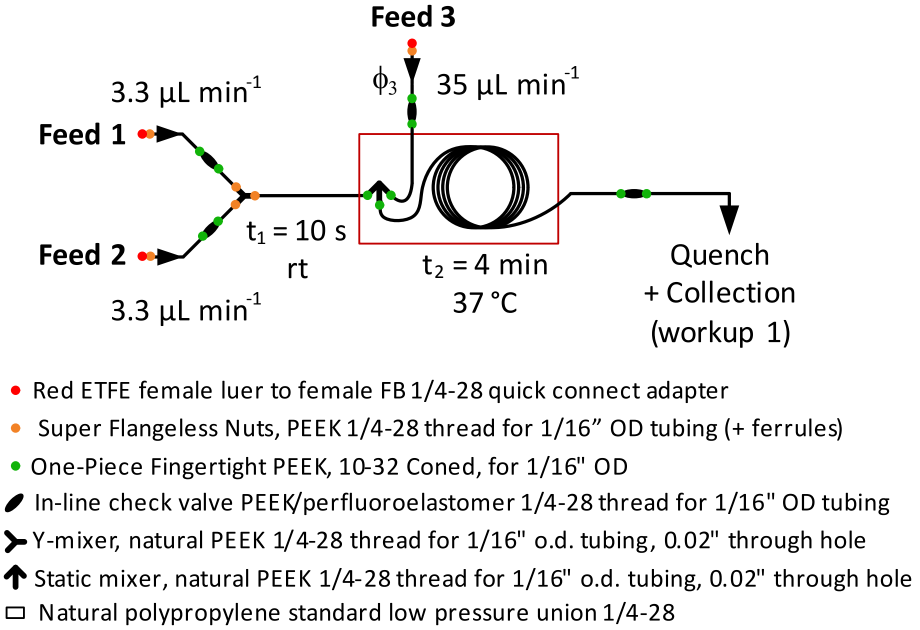

Supplementary Figure 18. Microfluidic setup for the experiment with MPA peptide thioester CILKEPVHGV-MPA 1c or MPAA peptide thioester CILKEPVHGV-MPA 2c.

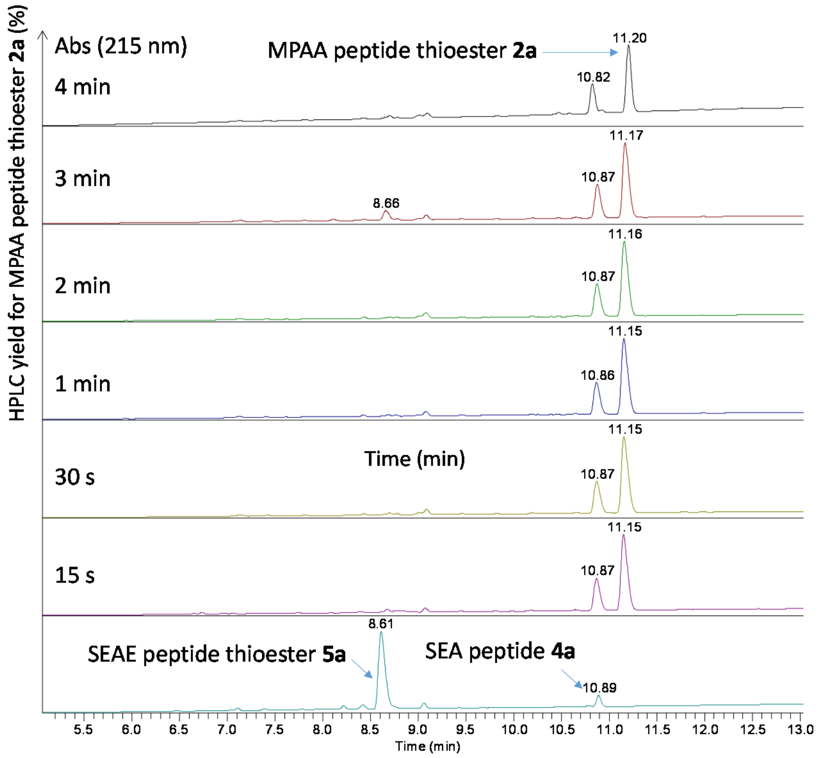

Supplementary Figure 19. Reaction of SEAE peptide 5a with MPAA. Kinetic study under microfluidic conditions. UPLC-MS analysis of the reaction for different residence times. Eluent A 0.10% TFA in water, eluent B 0.10% TFA in CH_3CN/water: Acquity UPLC-MS system, peptide BEH C18, 300 Å 1.7 µm (2.1 × 100 mm) column, gradient 0-40% B in 15 min (0.4 mL min^{-1}, UV 215 nm).

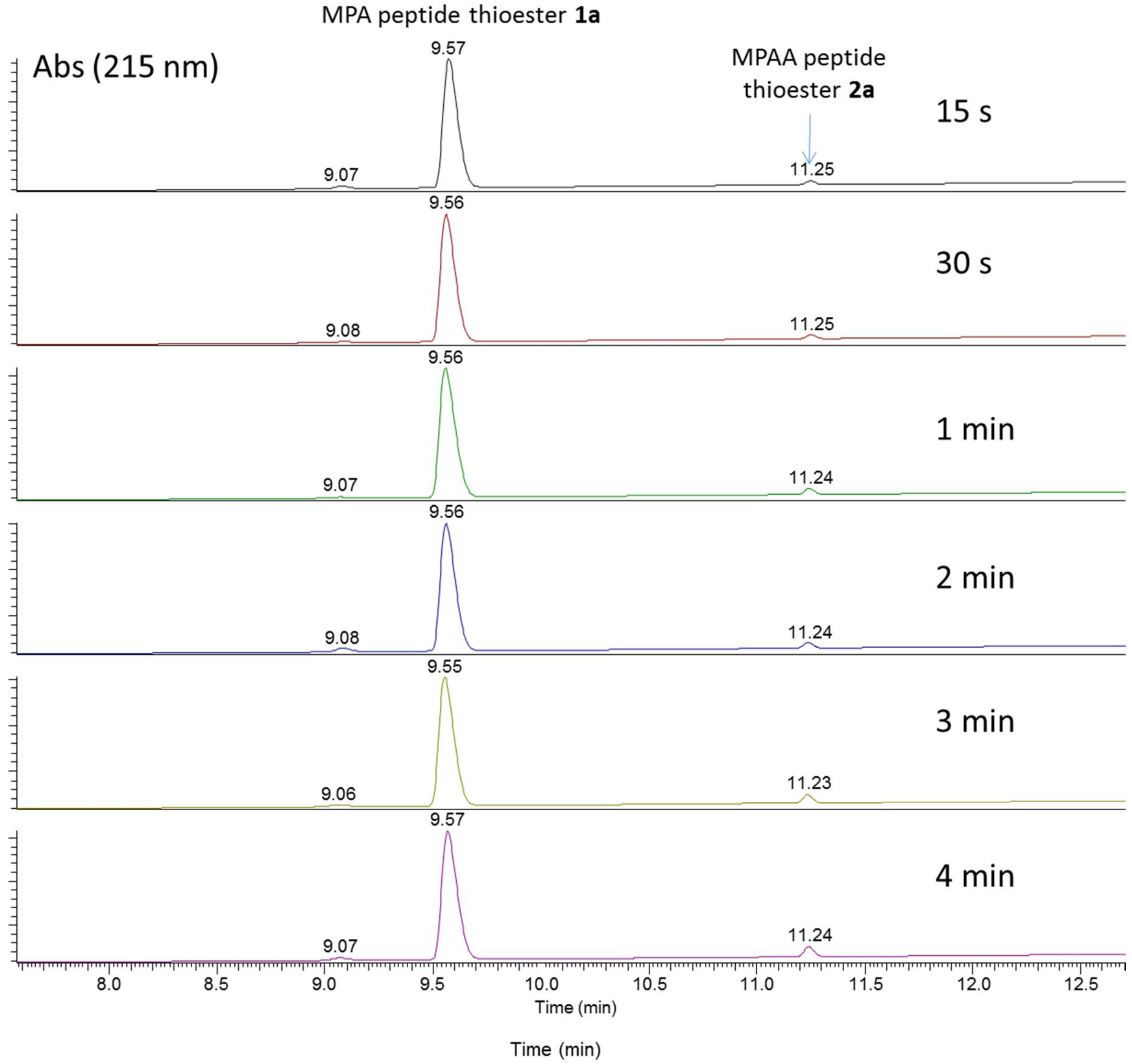

Supplementary Figure 20. Reaction of MPA peptide thioester 1a with MPAA. Kinetic study under microfluidic conditions. UPLC-MS analysis of the reaction for different residence times. Eluent A 0.10% TFA in water, eluent B 0.10% TFA in CH_3CN/water: Acquity UPLC-MS system, peptide BEH C18, 300 Å 1.7 µm (2.1 × 100 mm) column, gradient 0-40% B in 15 min (0.4 mL min^{-1}, UV 215 nm).

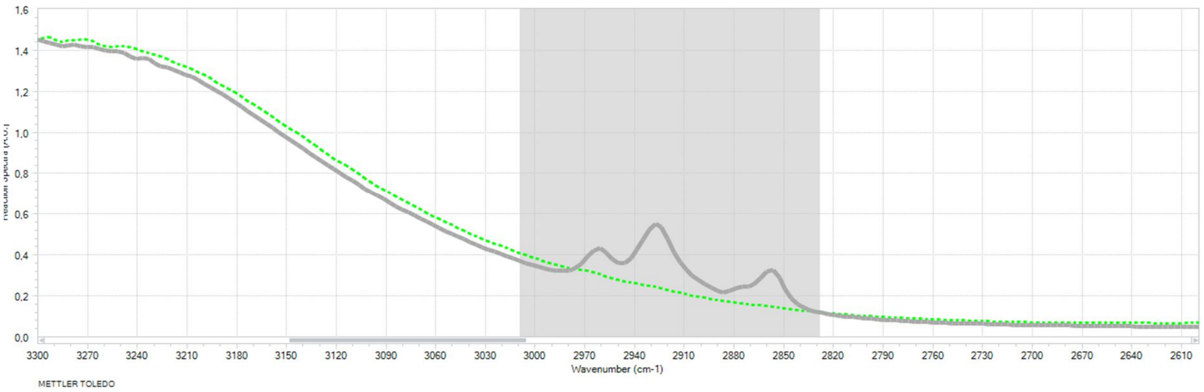

Supplementary Figure 21. In-line IR monitoring allowed identifying the nature of the gas (HCl) formed during the activation step. Green dotted line: experiment at 25 °C. Gray solid line: experiment at 90 °C without counter pressure.

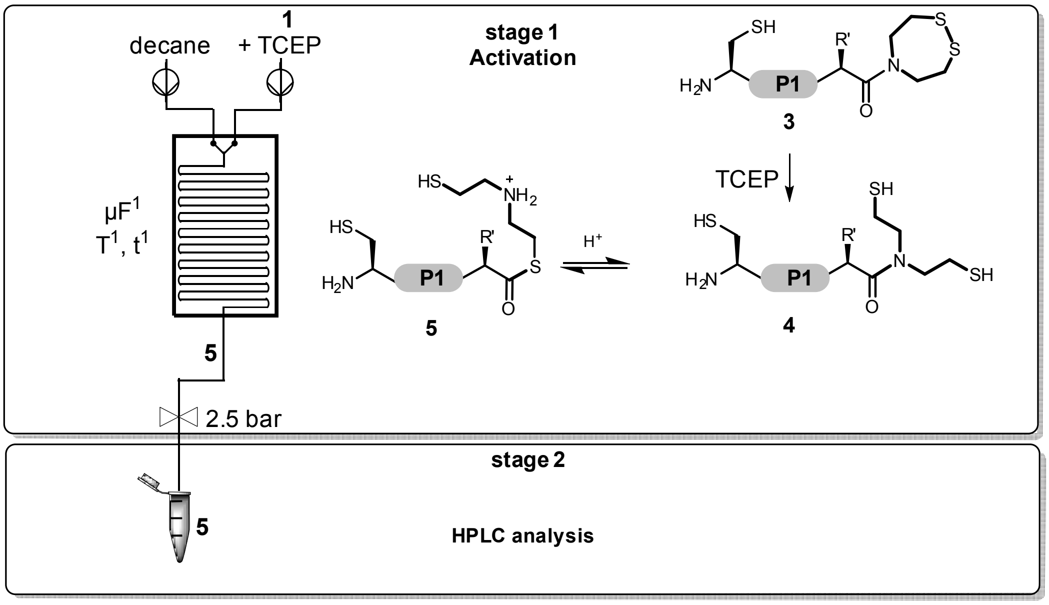

Supplementary Figure 22. Principle of the optimization of TCEP concentration in the microfluidic system.

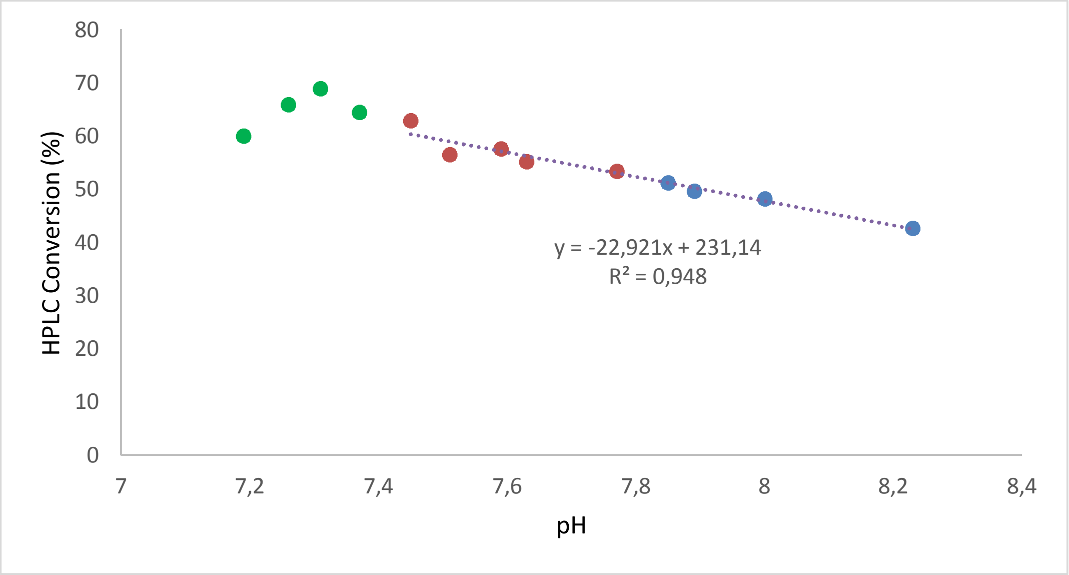

Supplementary Figure 23. Effect of pH in the second microreactor on the conversion of SEA^{on} peptide 4c into backbone cyclized peptide 7c.

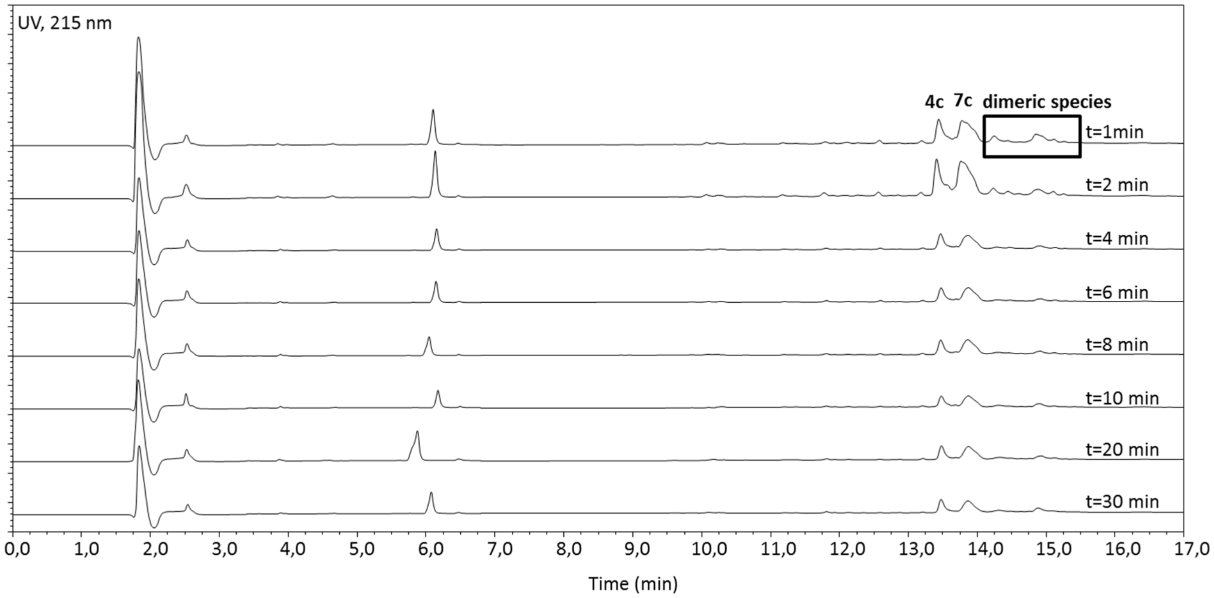

Supplementary Figure 24. Cyclization of peptide 4c. The residence time in µF^2 was varied from 30 min to 1 min.

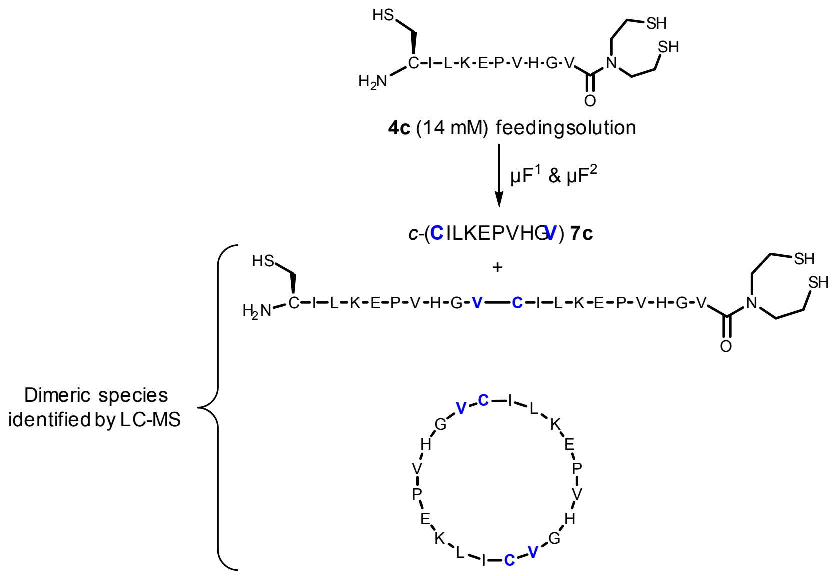

Supplementary Figure 25. Structure of the dimeric side-products formed in the microfluidic system using a Feed solution 1 at 14 mM (peptide 4c).

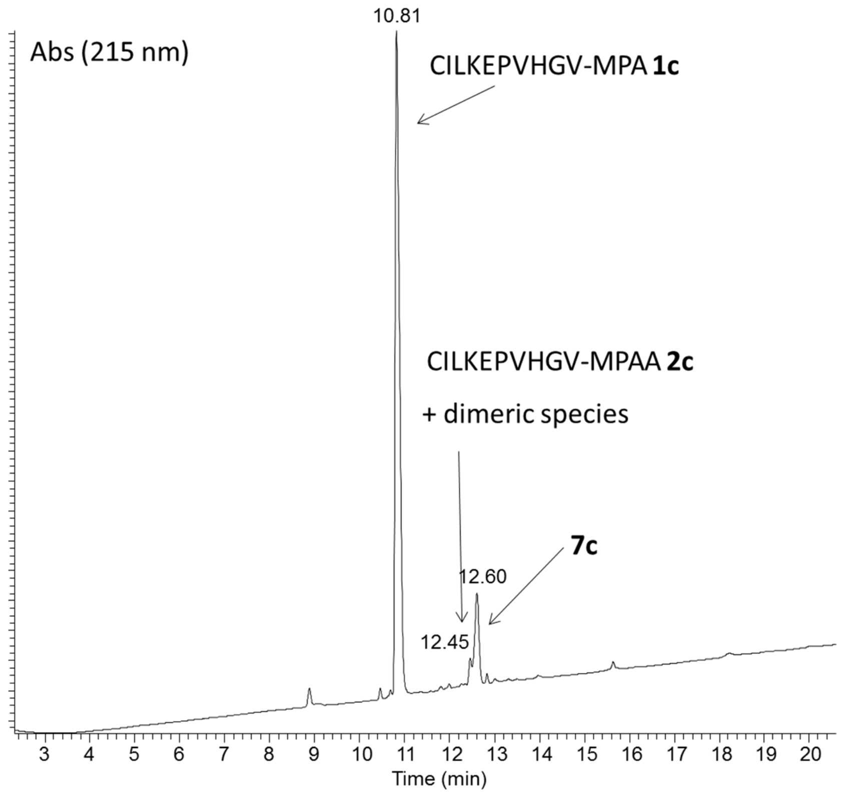

Supplementary Figure 26. Microfluidic the experiment with MPA peptide thioester CILKEPVHGV-MPA 1c. UPLC-MS analysis of the effluent after workup 1. Eluent A 0.10% TFA in water, eluent B 0.10% TFA in CH_3CN/water: Acquity UPLC-MS system, peptide BEH C18, 300 Å 1.7 µm (2.1 × 100 mm) column, gradient 0-40% B in 15 min (0.4 mL min^{-1}, UV 215 nm).

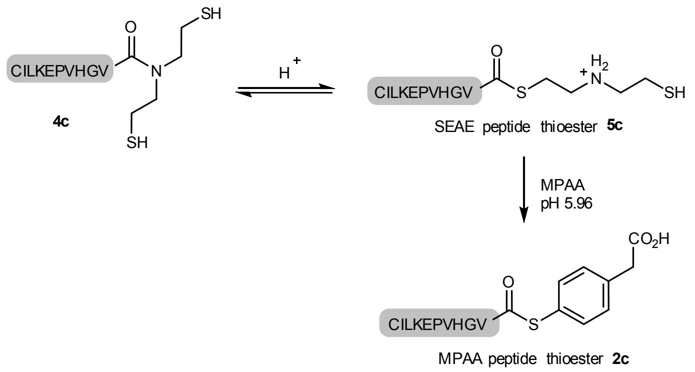

Supplementary Figure 27. Synthesis of MPAA peptide thioester 2c.

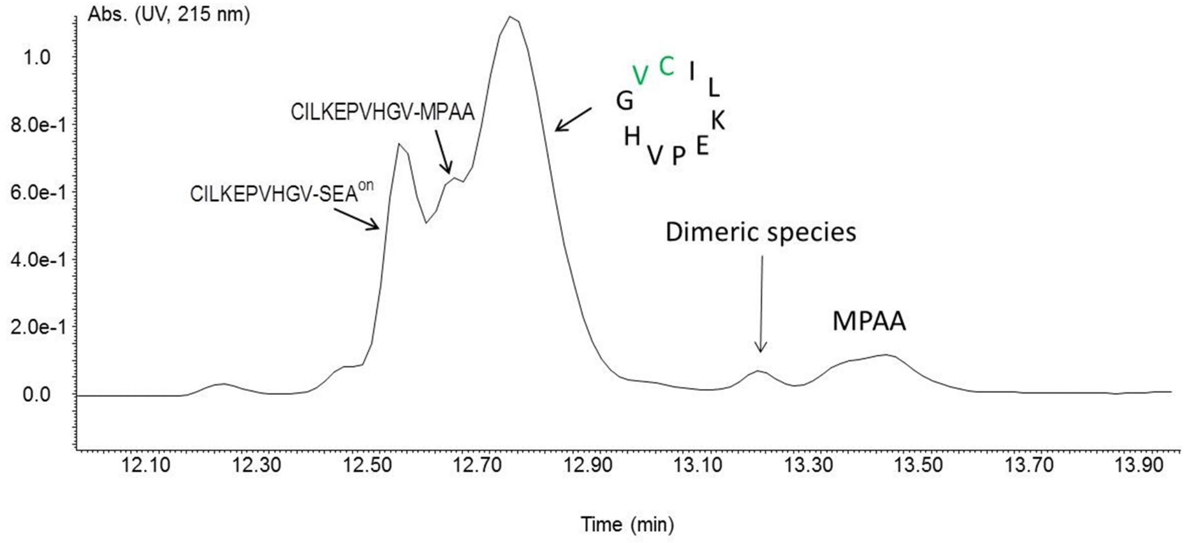

Supplementary Figure 28. Result of the microfluidic experiment with peptide 2c.

A)

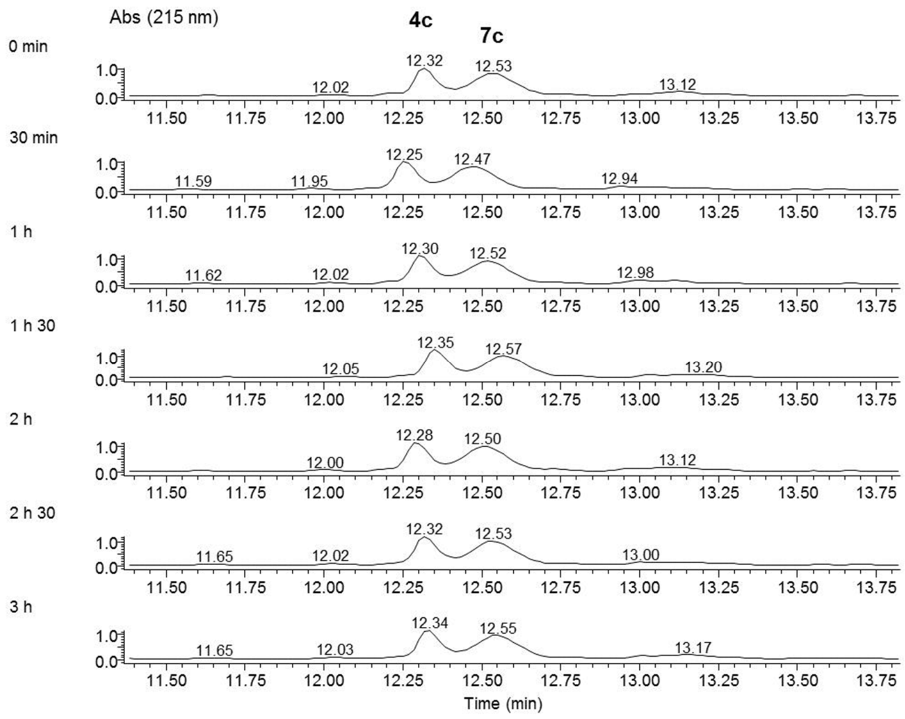

B)

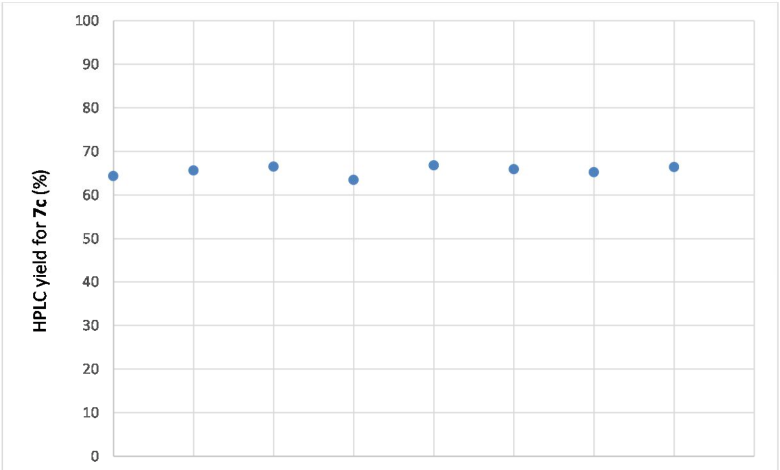

Supplementary Figure 29. Stability of the microfluidic system (synthesis of 7c from SEA^{on} peptide 4c). A) UPLC-MS chromatograms of the effluents collected at steady state. B) HPLC yield for peptide 7c as a function of time after the equilibration of the system.

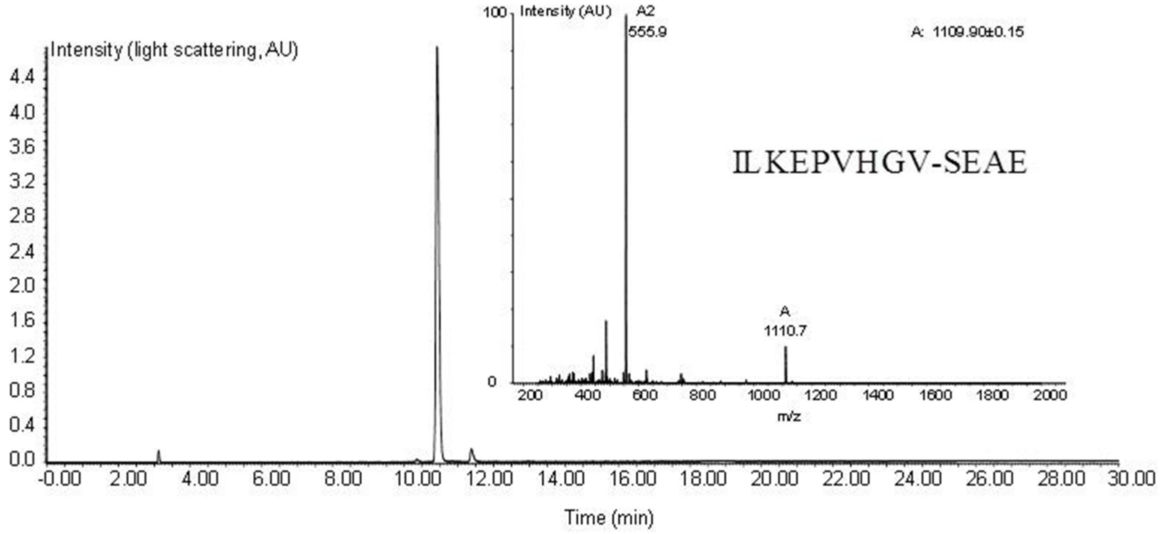

Supplementary Figure 30. LC-MS analysis of purified peptide 5a. LC trace, eluent A 0.10% TFA in water, eluent B 0.10% TFA in CH_3CN/water: 4/1 by vol. C18 Xbridge BEH 300 Å 5 µm (4.6 × 250 mm) column, gradient 0-100% B in 30 min (1 mL min^{-1}, detection light scattering). MS trace. $[M+H]^+$ m/z calcd. (monoisotopic) 1110.6, obs 1110.7.

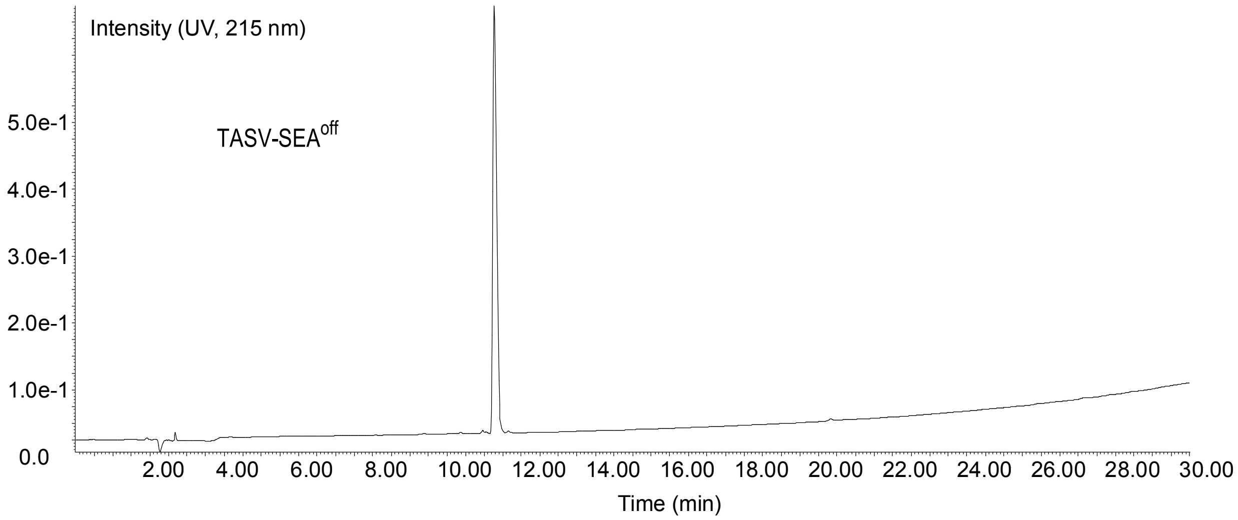

Supplementary Figure 31. RP-HPLC analysis of peptide 3b. Eluent A 0.10% TFA in water, eluent B 0.10% TFA in CH_3CN/water: 4/1 by vol. C18 Xbridge BEH 300 Å 5 µm (4.6 × 250 mm) column, gradient 0-100% B in 30 min (1 mL min^{-1}, detection 215 nm).

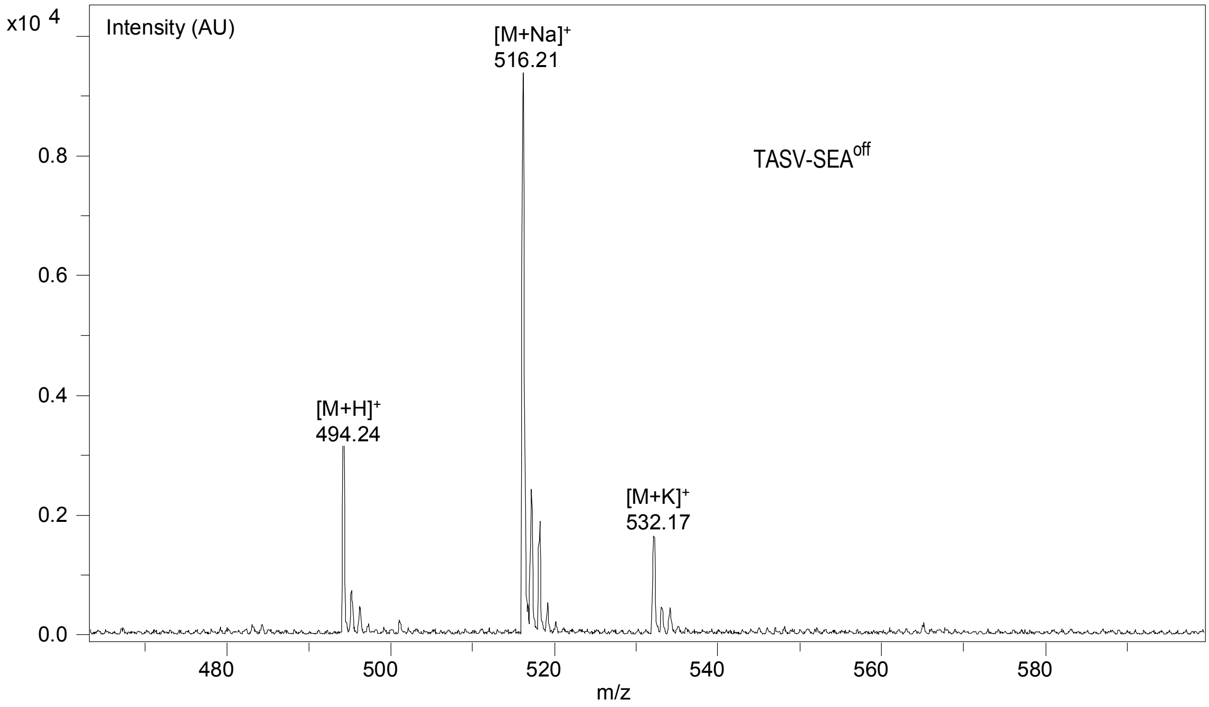

Supplementary Figure 32. MALDI-TOF analysis of peptide 3b. Matrix 2,5-dihydroxybenzoic acid (DHB), positive detection mode, [M+H]$^+$ calcd. (monoisotopic) 494.21, found 494.24.

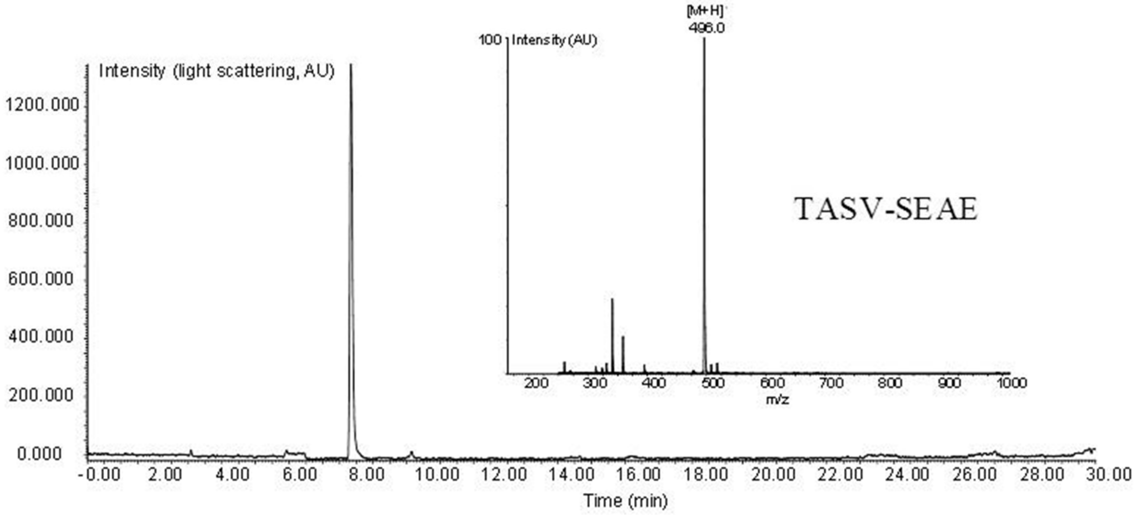

Supplementary Figure 33. LC-MS analysis of purified peptide 5b. LC trace, eluent A 0.10% TFA in water, eluent B 0.10% TFA in CH_3CN/water: 4/1 by vol. C18 Xbridge BEH 300 Å 5 µm (4.6 × 250 mm) column, gradient 0-100% B in 30 min (1 mL min^{-1}, detection light scattering). MS trace. [M+H]$^+$ m/z calcd. (monoisotopic) 496.2, obs 496.0.

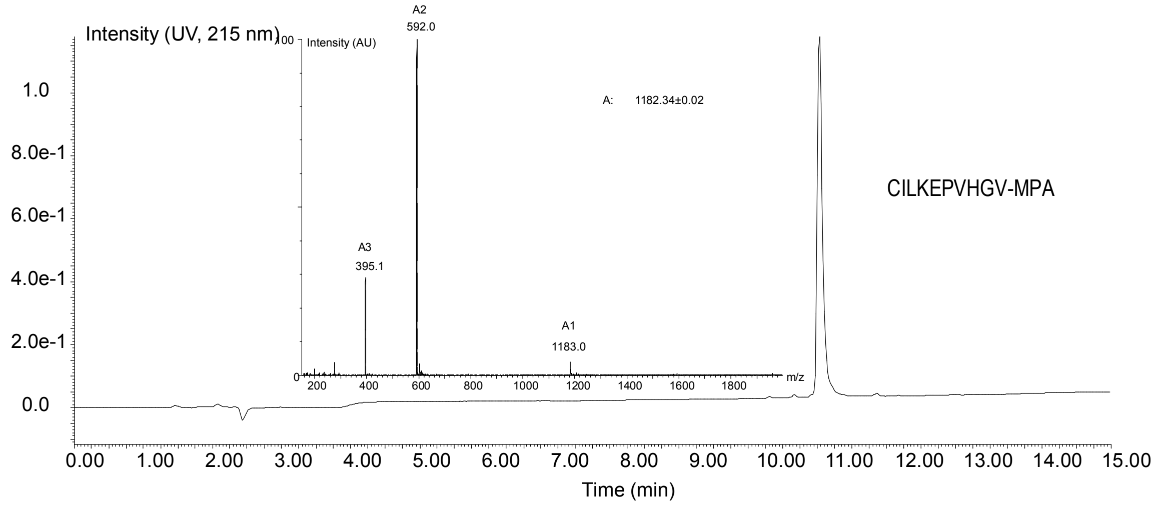

Supplementary Figure 34. LC-MS analysis of peptide 1c. LC trace, eluent A 0.10% TFA in water, eluent B 0.10% TFA in CH_3CN/water: 4/1 by vol. C18 Xbridge BEH 300 Å 5 µm (4.6 × 250 mm) column, gradient 0-50% B in 15 min (1 mL min^{-1}, detection UV 215 nm). MS trace. $[M+H]^+$ m/z calcd. (monoisotopic) 1182.6, obs 1183.0, $[M+2H]^{2+}$ m/z calcd. (monoisotopic) 591.8, obs 592.0, $[M+3H]^{3+}$ m/z calcd. (monoisotopic) 394.9, obs 395.1.

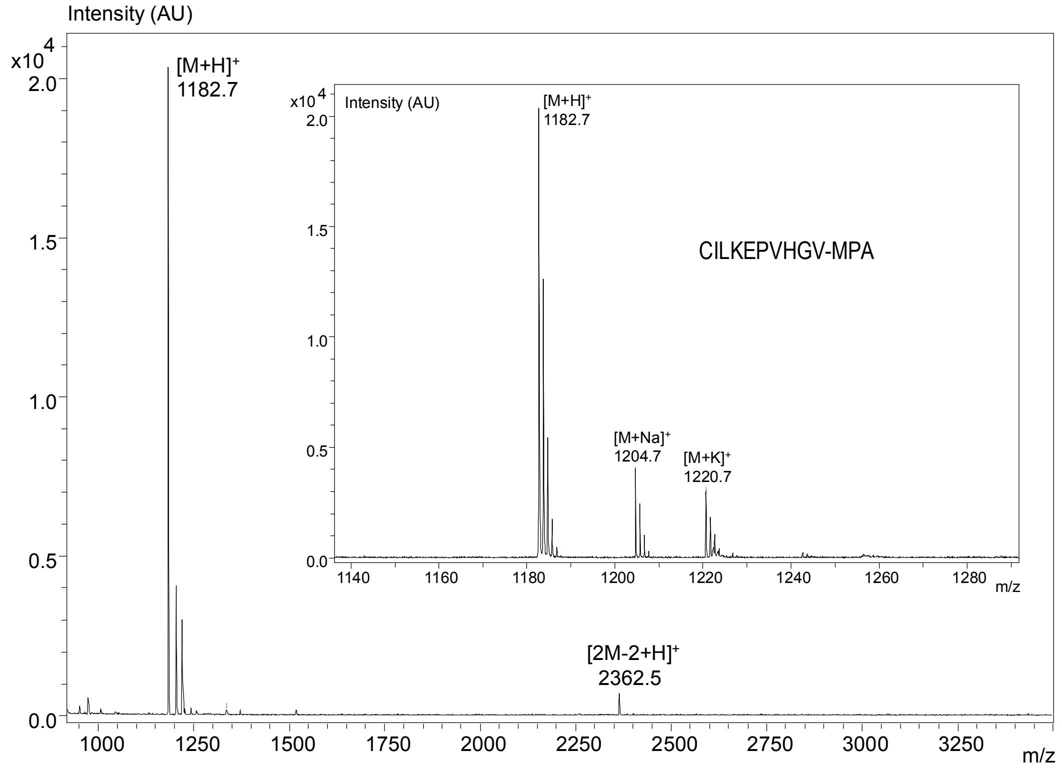

Supplementary Figure 35. MALDI-TOF analysis of peptide **1c**. Matrix alpha cyano 4-hydroxycinnaminic acid, positive detection mode, $[M+H]^+$ calcd. (monoisotopic) 1182.6, found 1182.7.

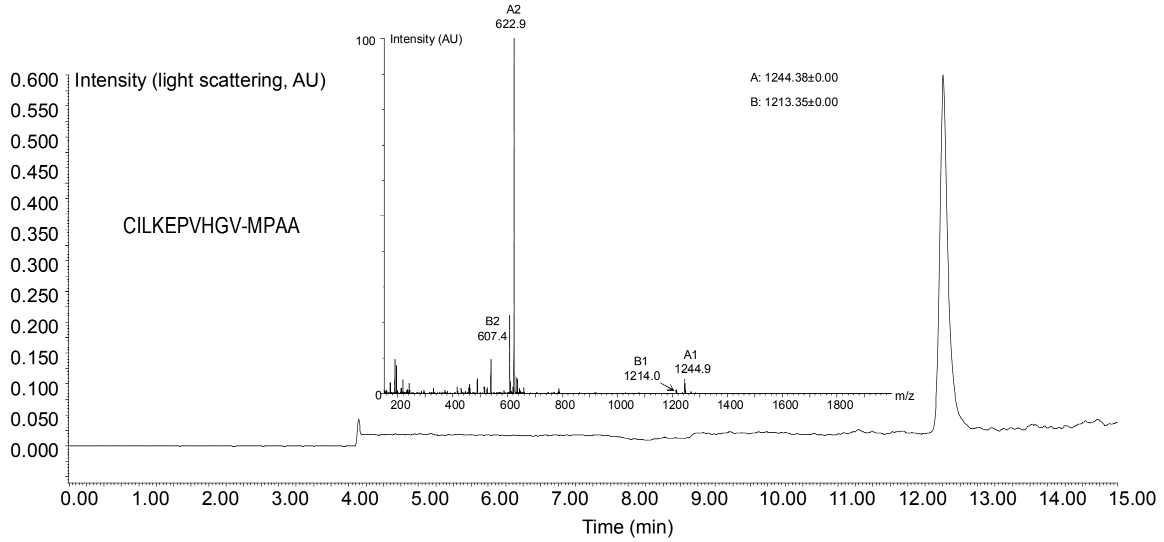

Supplementary Figure 36. LC-MS analysis of peptide 2c. LC trace, eluent A 0.10% TFA in water, eluent B 0.10% TFA in CH_3CN/water: 4/1 by vol. C18 Xbridge BEH 300 Å 5 µm (4.6 × 250 mm) column, gradient 0-50% B in 15 min (1 mL min^{-1}, detection light scattering). MS trace. $[M+H]^+$ m/z calcd. (monoisotopic) 1244.6, obs 1244.9, $[M+2H]^{2+}$ m/z calcd. (monoisotopic) 622.8, obs 622.9, contaminated by CILKEPVHGV-SEA^{on}, $[M'+H]^+$ m/z calcd. (monoisotopic) 1213.6, obs 1214.0, $[M'+2H]^{2+}$ m/z calcd. (monoisotopic) 607.3, obs 607.4.

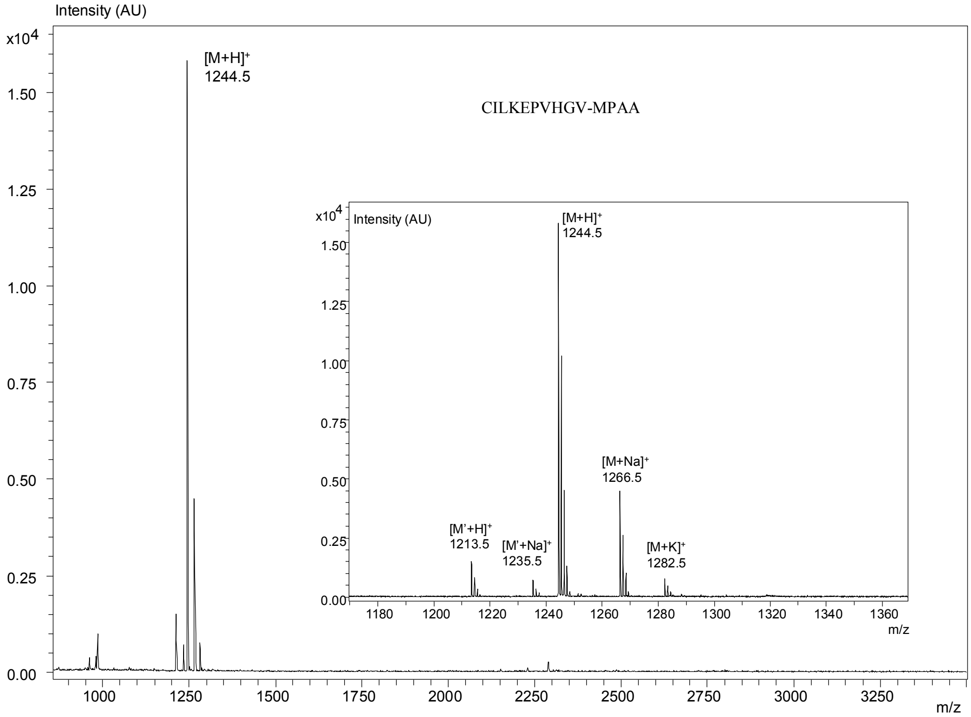

Supplementary Figure 37. MALDI-TOF analysis of peptide 2c. Matrix alpha cyano 4-hydroxycinnaminic acid, positive detection mode, $[M+H]^+$ calcd. (monoisotopic) 1244.6, found 1244.5.

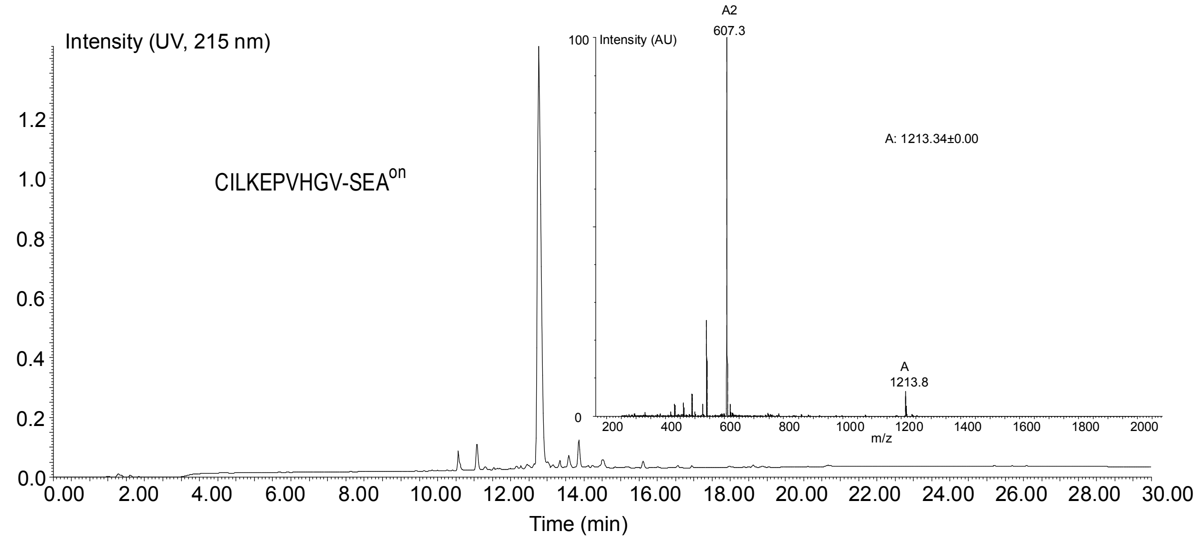

Supplementary Figure 38. LC-MS analysis of peptide 4c. LC trace, eluent A 0.10% TFA in water, eluent B 0.10% TFA in CH_3CN/water: 4/1 by vol. C18 Xbridge BEH 300 Å 5 µm (4.6 × 250 mm) column, gradient 0-100% B in 30 min (1 mL min^{-1}, detection 215 nm). MS trace. $[M+H]^+$ m/z calcd. (monoisotopic) 1213.6, obs 1213.8.

A)

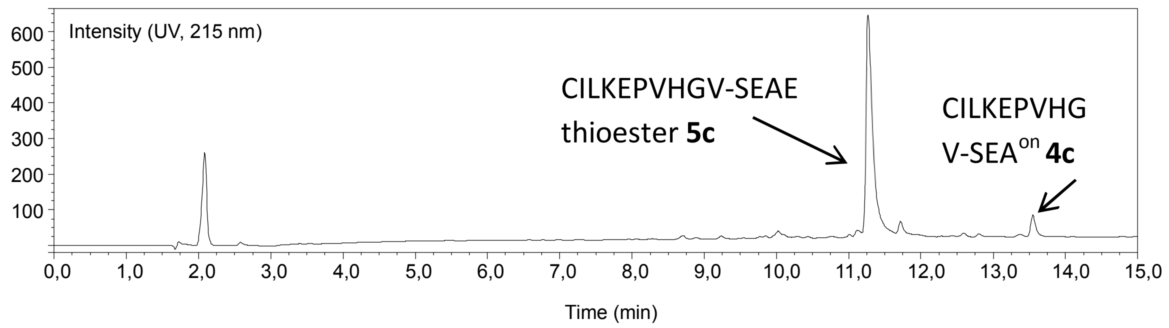

B)

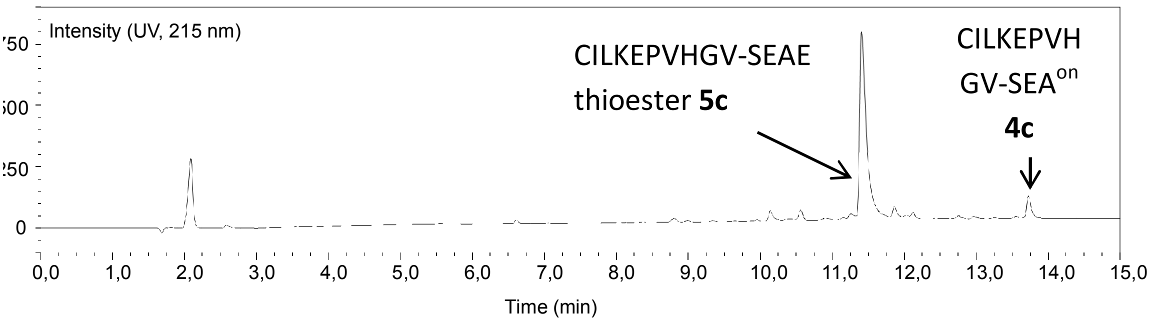

C)

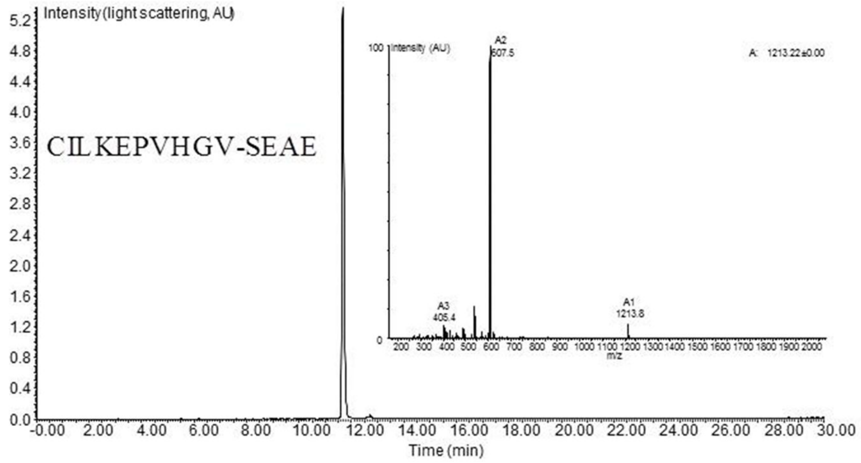

Supplementary Figure 39. Synthesis of 5c in batch and under microfluidic conditions. RP-HPLC analysis of the activation of peptide **4c** into SEAE peptide **5c** peptide at pH=1, 100 mM TCEP, 1h, 90°C. A) Under microfluidic conditions using decane and pressure regulator. Eluent A 0.10% TFA in water, eluent B 0.10% TFA in CH_3CN/water: 4/1 by vol. C18 Xbridge BEH 300 Å 5 µm (4.6 × 250 mm) column, gradient 0-50% B in 15 min (1 mL min^{-1}, detection UV, 215 nm).; B) In batch, see A) for HPLC conditions; C) After HPLC purification. LC trace, eluent A 0.10% TFA in water, eluent B 0.10% TFA in CH_3CN/water: 4/1 by vol. C18 Xbridge BEH 300 Å 5 µm (4.6 × 250 mm) column, gradient 0-100% B in 30 min (1 mL min^{-1}, detection light scattering). MS trace. $[M+H]^+$ m/z calcd. (monoisotopic) 1213.6, obs 1213.8.

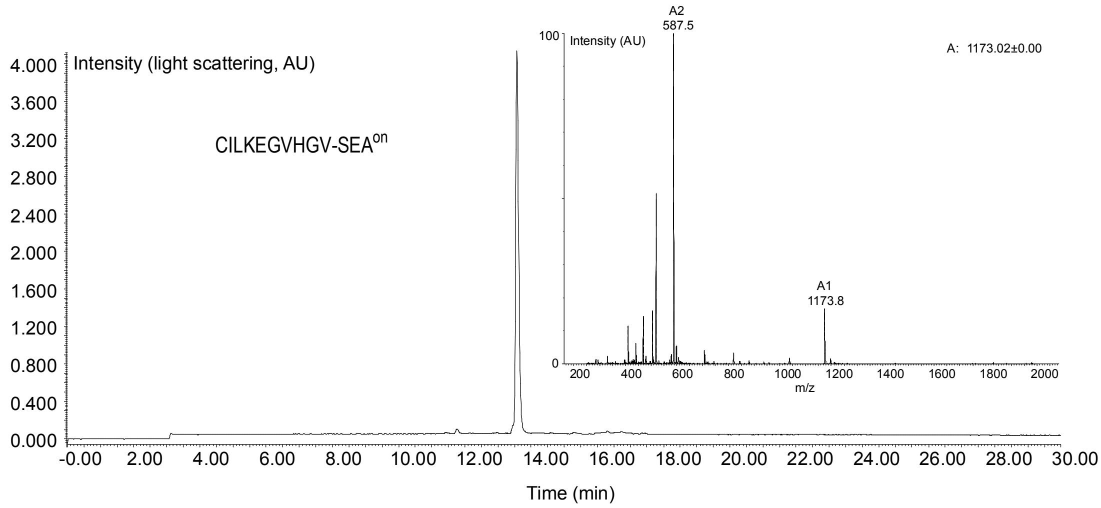

Supplementary Figure 40. LC-MS analysis of peptide 4d. LC trace, eluent A 0.10% TFA in water, eluent B 0.10% TFA in CH_3CN/water: 4/1 by vol. C18 Xbridge BEH 300 Å 5 µm (4.6 × 250 mm) column, gradient 0-100% B in 30 min (1 mL min^{-1}, detection light scattering). MS trace. $[M+H]^+$ m/z calcd. (monoisotopic) 1173.6, obs 1173.8.

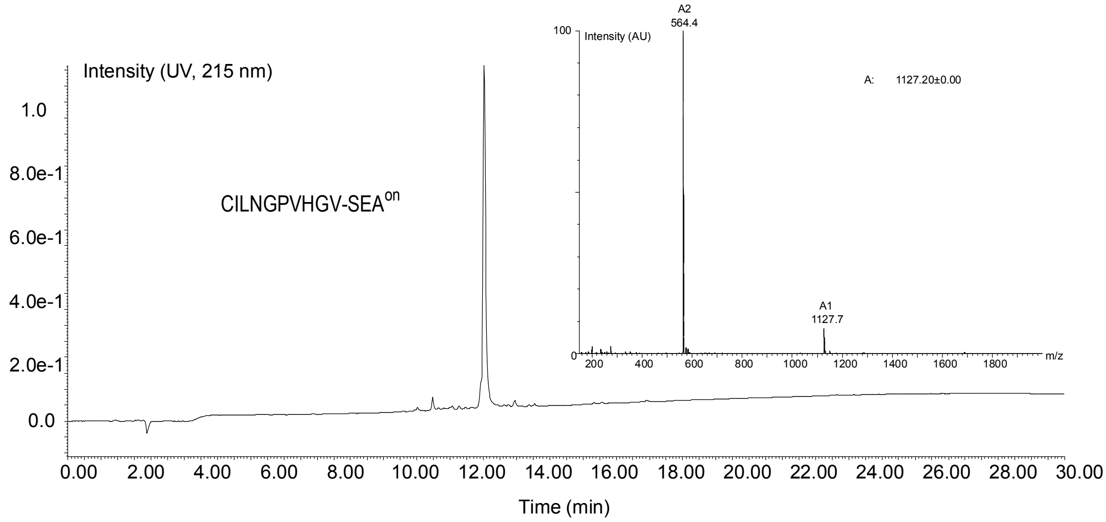

Supplementary Figure 41. LC-MS analysis of peptide 4e. LC trace, eluent A 0.10% TFA in water, eluent B 0.10% TFA in CH_3CN/water: 4/1 by vol. C18 Xbridge BEH 300 Å 5 µm (4.6 × 250 mm) column, gradient 0-100% B in 30 min (1 mL min^{-1}, detection UV, 215 nm). MS trace. $[M+H]^+$ m/z calcd. (monoisotopic) 1127.5, obs 1127.7, $[M+2H]^{2+}$ m/z calcd. (monoisotopic) 564.3, obs 564.4.

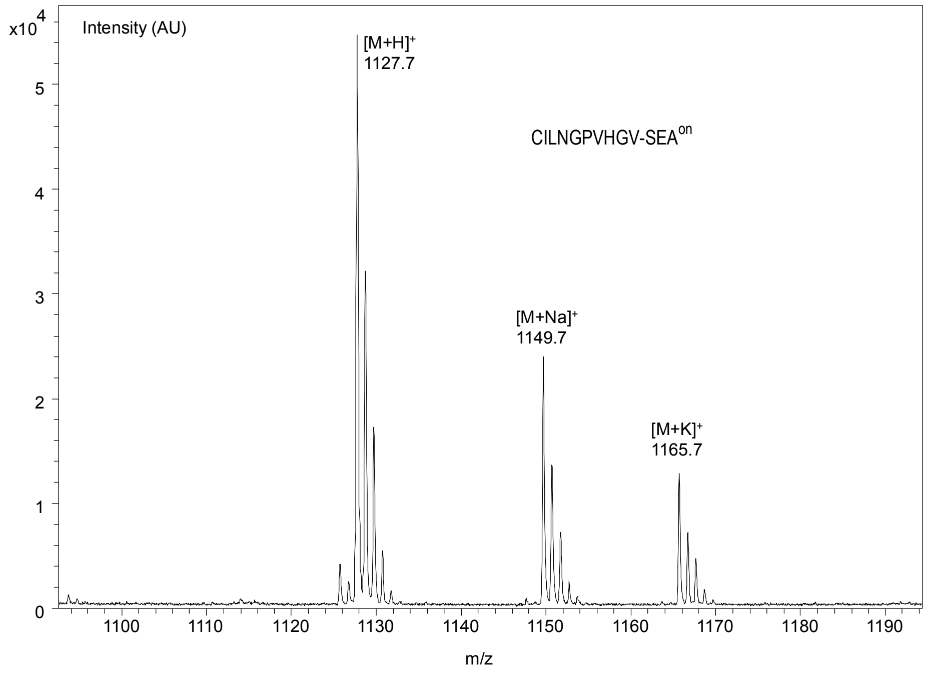

Supplementary Figure 42. MALDI-TOF analysis of peptide 4e. Matrix alpha cyano 4-hydroxycinnaminic acid, positive detection mode, $[M+H]^+$ calcd. (monoisotopic) 1127.5, found 1127.7.

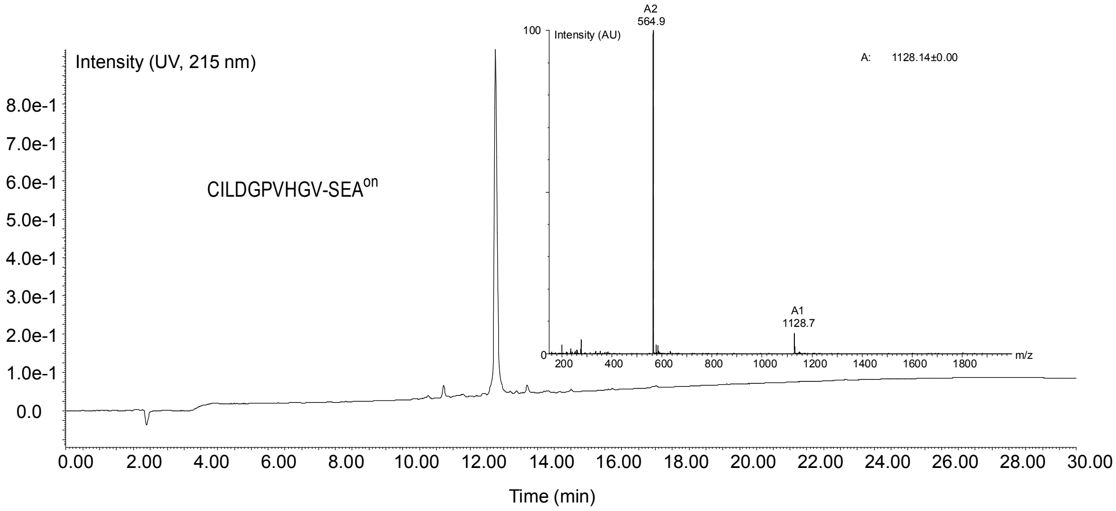

Supplementary Figure 43. LC-MS analysis of peptide 4f. LC trace, eluent A 0.10% TFA in water, eluent B 0.10% TFA in CH_3CN/water: 4/1 by vol. C18 Xbridge BEH 300 Å 5 µm (4.6 × 250 mm) column, gradient 0-100% B in 30 min (1 mL min^{-1}, detection UV, 215 nm). MS trace. $[M+H]^+$ m/z calcd. (monoisotopic) 1128.5, obs 1128.7, $[M+2H]^{2+}$ m/z calcd. (monoisotopic) 564.8, obs 564.9.

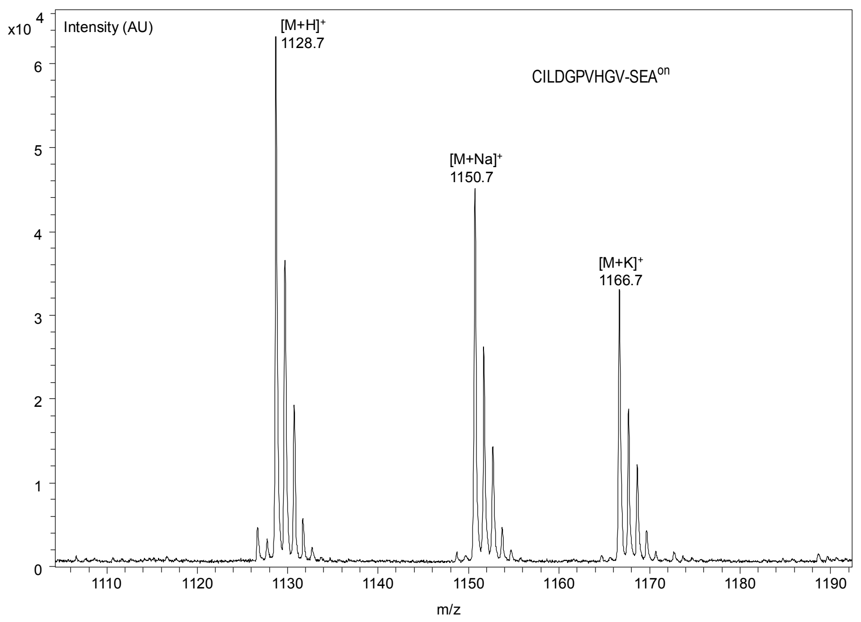

Supplementary Figure 44. MALDI-TOF analysis of peptide 4f. Matrix alpha cyano 4-hydroxycinnaminic acid, positive detection mode, $[M+H]^+$ calcd. (monoisotopic) 1128.5, found 1128.7.

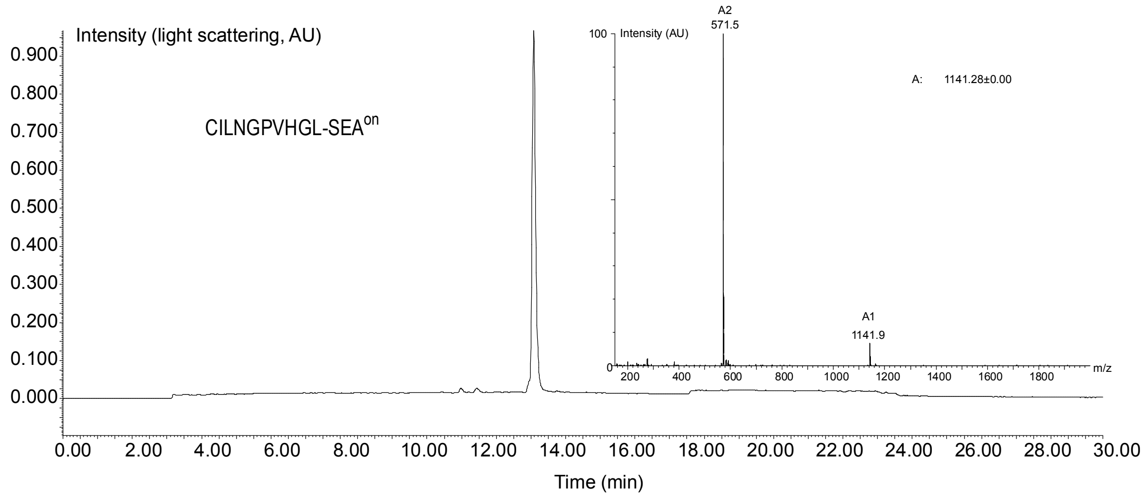

Supplementary Figure 45. LC-MS analysis of peptide 4g. LC trace, eluent A 0.10% TFA in water, eluent B 0.10% TFA in CH_3CN/water: 4/1 by vol. C18 Xbridge BEH 300 Å 5 µm (4.6 × 250 mm) column, gradient 0-100% B in 30 min (1 mL min^{-1}, detection light scattering). MS trace. $[M+H]^+$ m/z calcd. (monoisotopic) 1141.6, obs 1141.9, $[M+2H]^{2+}$ m/z calcd. (monoisotopic) 571.3, obs 571.5.

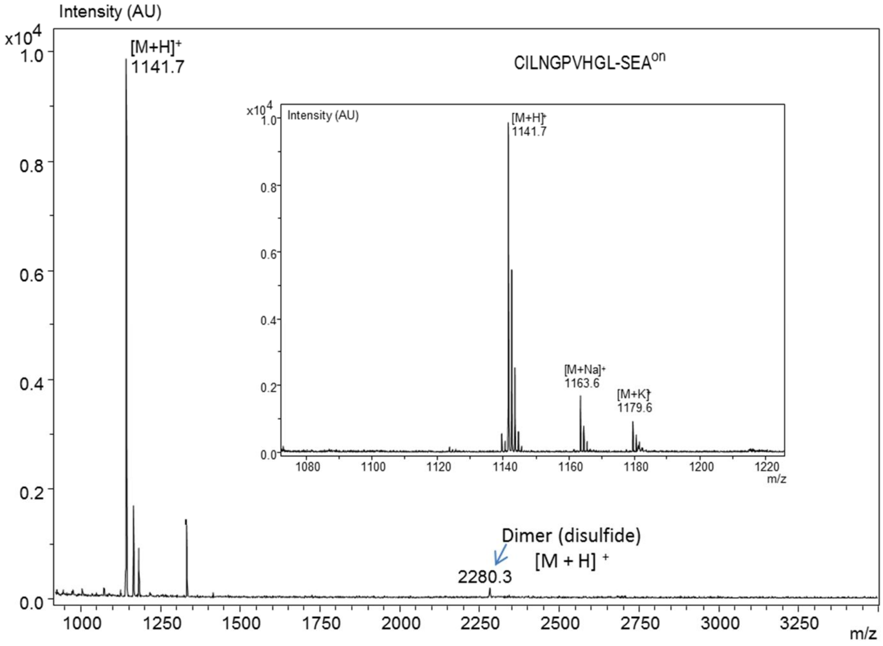

Supplementary Figure 46. MALDI-TOF analysis of peptide 4g. Matrix alpha cyano 4-hydroxycinnaminic acid, positive detection mode, $[M+H]^+$ calcd. (monoisotopic) 1141.6, found 1141.7.

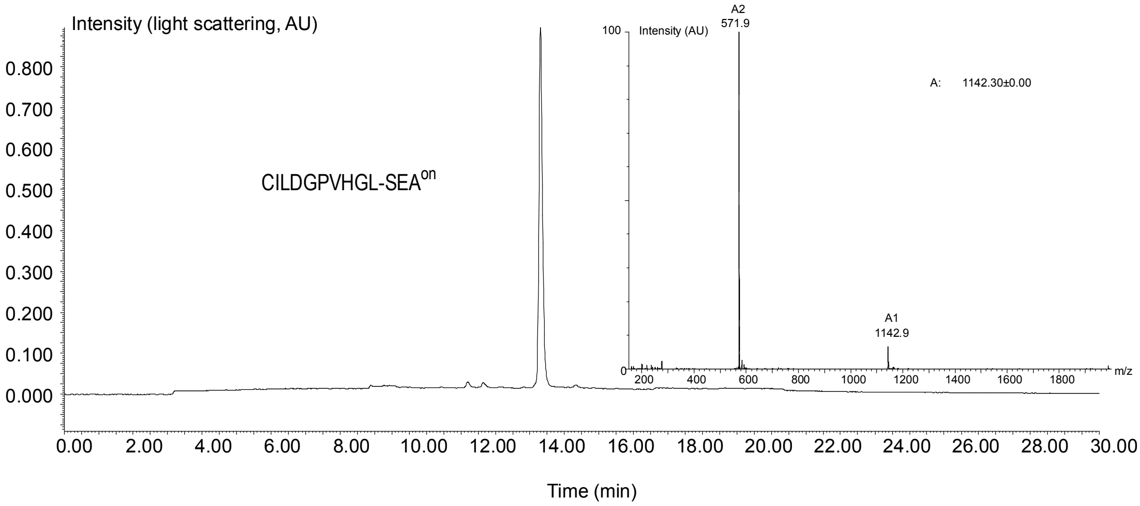

Supplementary Figure 47. LC-MS analysis of peptide 4h. LC trace, eluent A 0.10% TFA in water, eluent B 0.10% TFA in CH_3CN/water: 4/1 by vol. C18 Xbridge BEH 300 Å 5 µm (4.6 × 250 mm) column, gradient 0-100% B in 30 min (1 mL min^{-1}, detection light scattering). MS trace. $[M+H]^+$ m/z calcd. (monoisotopic) 1142.6, obs 1142.9, $[M+2H]^{2+}$ m/z calcd. (monoisotopic) 571.8, obs 571.9.

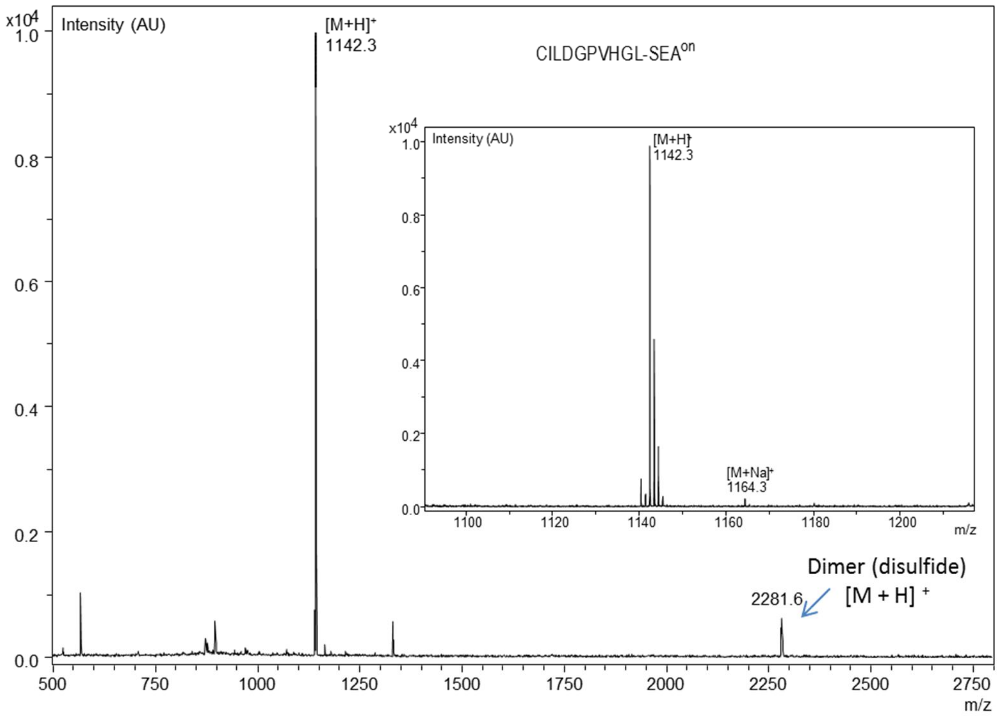

Supplementary Figure 48. MALDI-TOF analysis of peptide 4h. Matrix alpha cyano 4-hydroxycinnaminic acid, positive detection mode, $[M+H]^+$ calcd. (monoisotopic) 1142.6, found 1142.3.

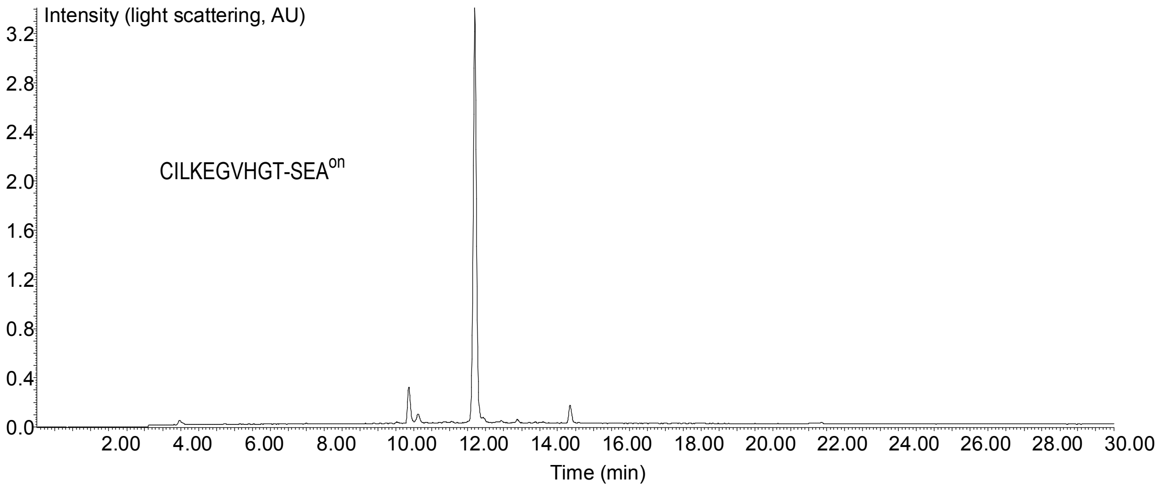

Supplementary Figure 49. RP-HPLC analysis of peptide 4i. Eluent A 0.10% TFA in water, eluent B 0.10% TFA in CH_3CN/water: 4/1 by vol. C18 Xbridge BEH 300 Å 5 µm (4.6 × 250 mm) column, gradient 0-100% B in 30 min (1 mL min^{-1}, detection light scattering).

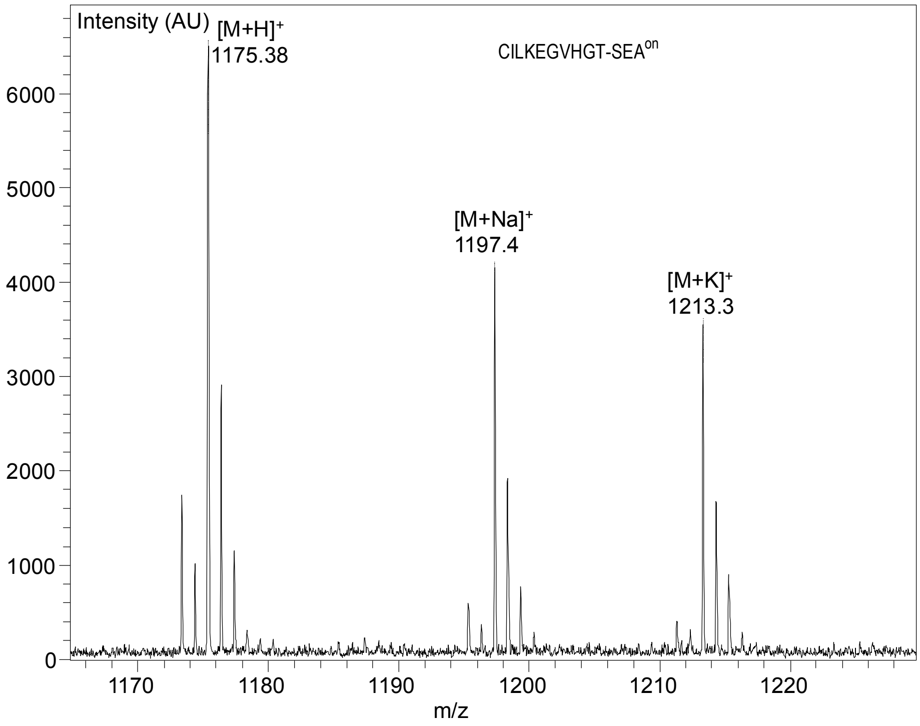

Supplementary Figure 50. MALDI-TOF analysis of peptide 4i. Matrix alpha cyano 4-hydroxycinnaminic acid, positive detection mode, $[M+H]^+$ calcd. (monoisotopic) 1175.6, found 1175.4.

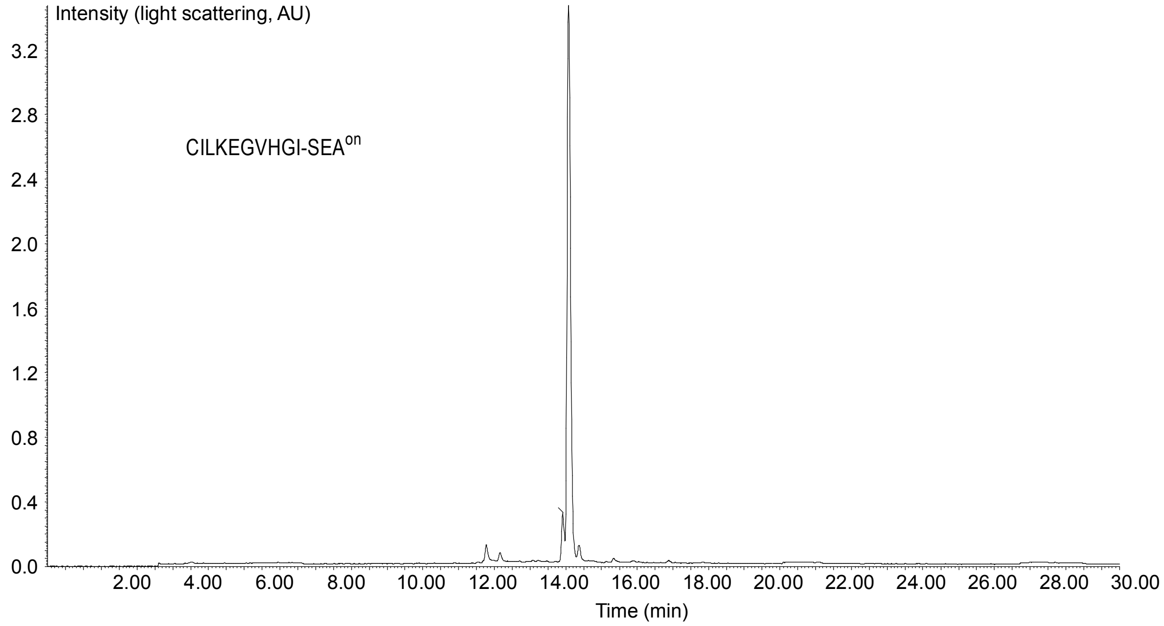

Supplementary Figure 51. RP-HPLC analysis of peptide 4j. Eluent A 0.10% TFA in water, eluent B 0.10% TFA in CH_3CN/water: 4/1 by vol. C18 Xbridge BEH 300 Å 5 µm (4.6 × 250 mm) column, gradient 0-100% B in 30 min (1 mL min^{-1}, detection light scattering).

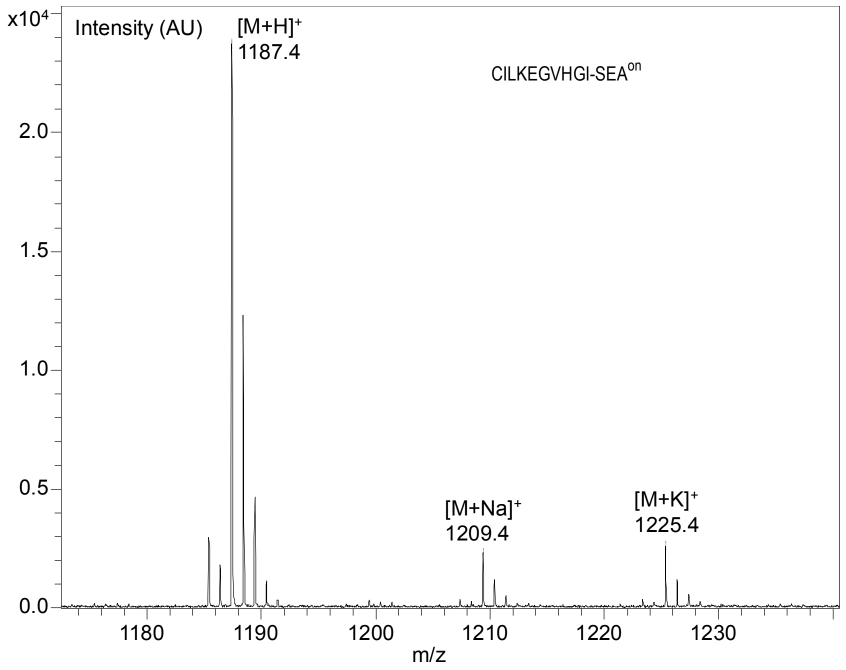

Supplementary Figure 52. MALDI-TOF analysis of peptide 4j. Matrix alpha cyano 4-hydroxycinnaminic acid, positive detection mode, $[M+H]^+$ calcd. (monoisotopic) 1187.6, found 1187.4.

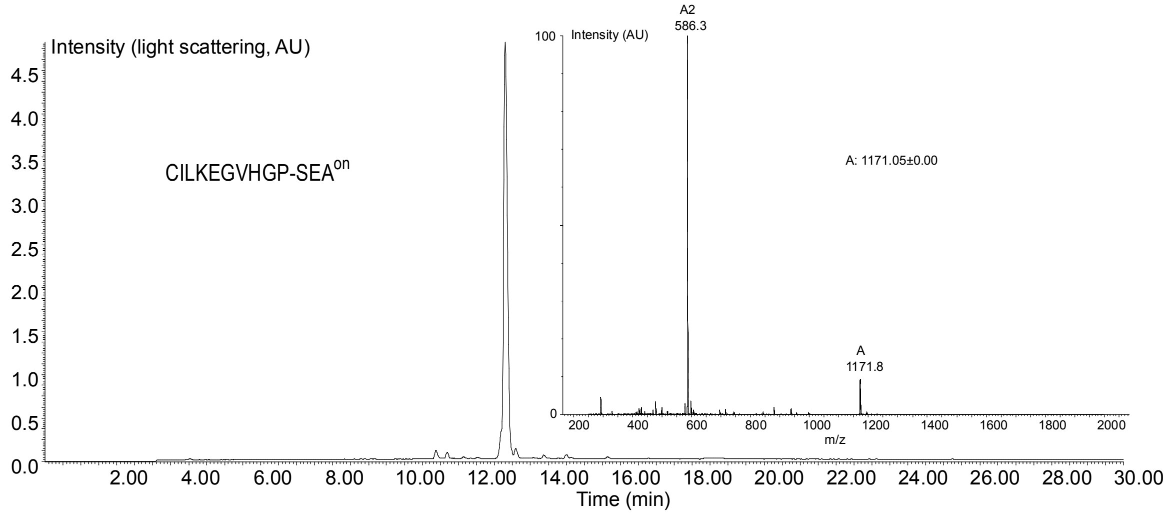

Supplementary Figure 53. LC-MS analysis of peptide 4k. LC trace, eluent A 0.10% TFA in water, eluent B 0.10% TFA in CH_3CN/water: 4/1 by vol. C18 Xbridge BEH 300 Å 5 µm (4.6 × 250 mm) column, gradient 0-100% B in 30 min (1 mL min^{-1}, detection light scattering). MS trace. $[M+H]^+$ m/z calcd. (monoisotopic) 1171.6, obs 1171.8.

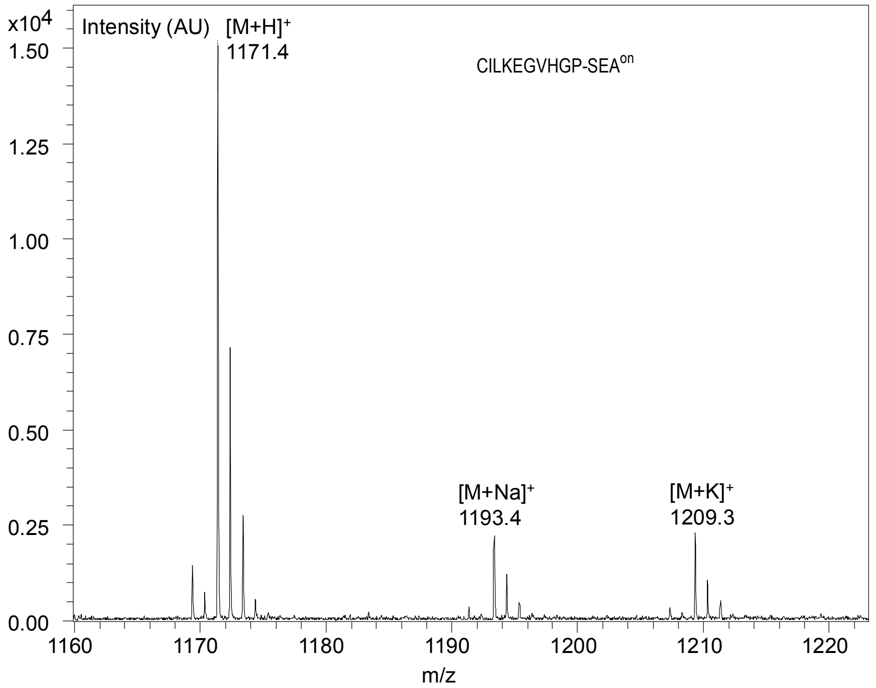

Supplementary Figure 54. MALDI-TOF analysis of peptide 4k. Matrix alpha cyano 4-hydroxycinnaminic acid, positive detection mode, $[M+H]^+$ calcd. (monoisotopic) 1171.6, found 1171.4.

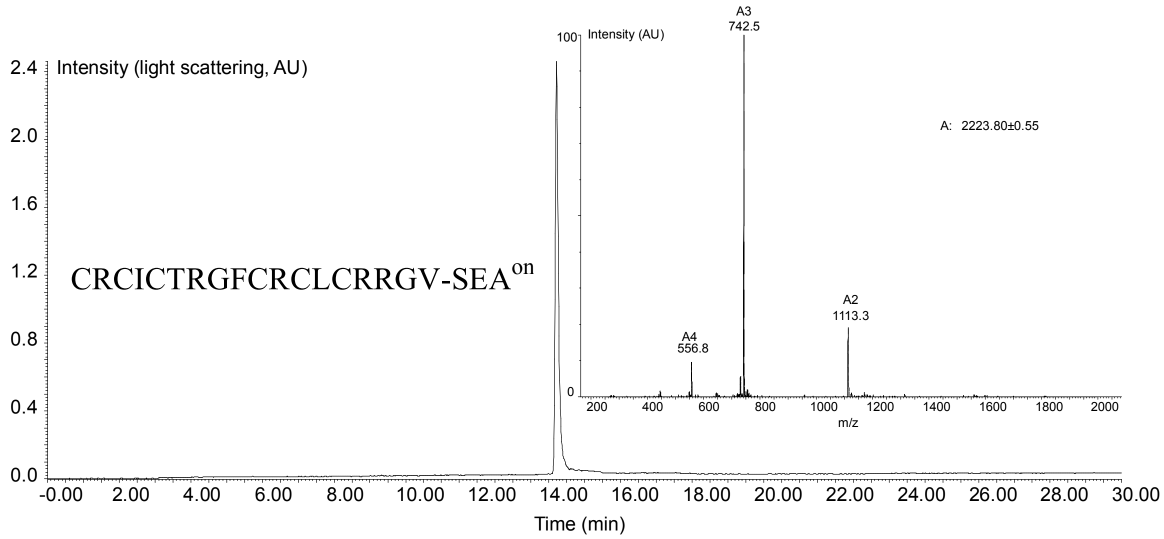

Supplementary Figure 55. LC-MS analysis of peptide 4l. LC trace, eluent A 0.10% TFA in water, eluent B 0.10% TFA in CH_3CN/water: 4/1 by vol. C18 Xbridge BEH 300 Å 5 µm (4.6 × 250 mm) column, gradient 0-100% B in 30 min (1 mL min^{-1}, detection light scattering). MS trace. $[M+2H]^{2+}$ m/z calcd. (monoisotopic) 1112.5, obs 1113.3, $[M+3H]^{3+}$ m/z calcd. (monoisotopic) 742.0, obs 742.5, $[M+4H]^{4+}$ m/z calcd. (monoisotopic) 556.8, obs 556.8.

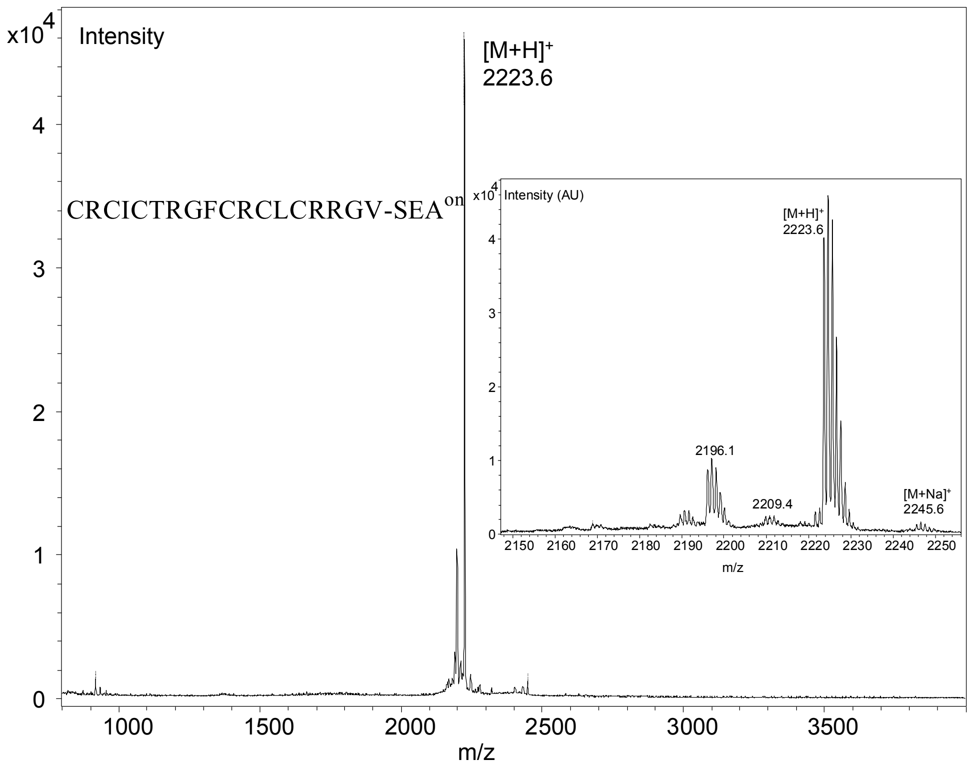

Supplementary Figure 56. MALDI-TOF analysis of peptide 4l. Matrix alpha cyano 4-hydroxycinnaminic acid, positive detection mode, reflected mode, $[M+H]^+$ calcd. (monoisotopic) 2223.99, found 2223.6.

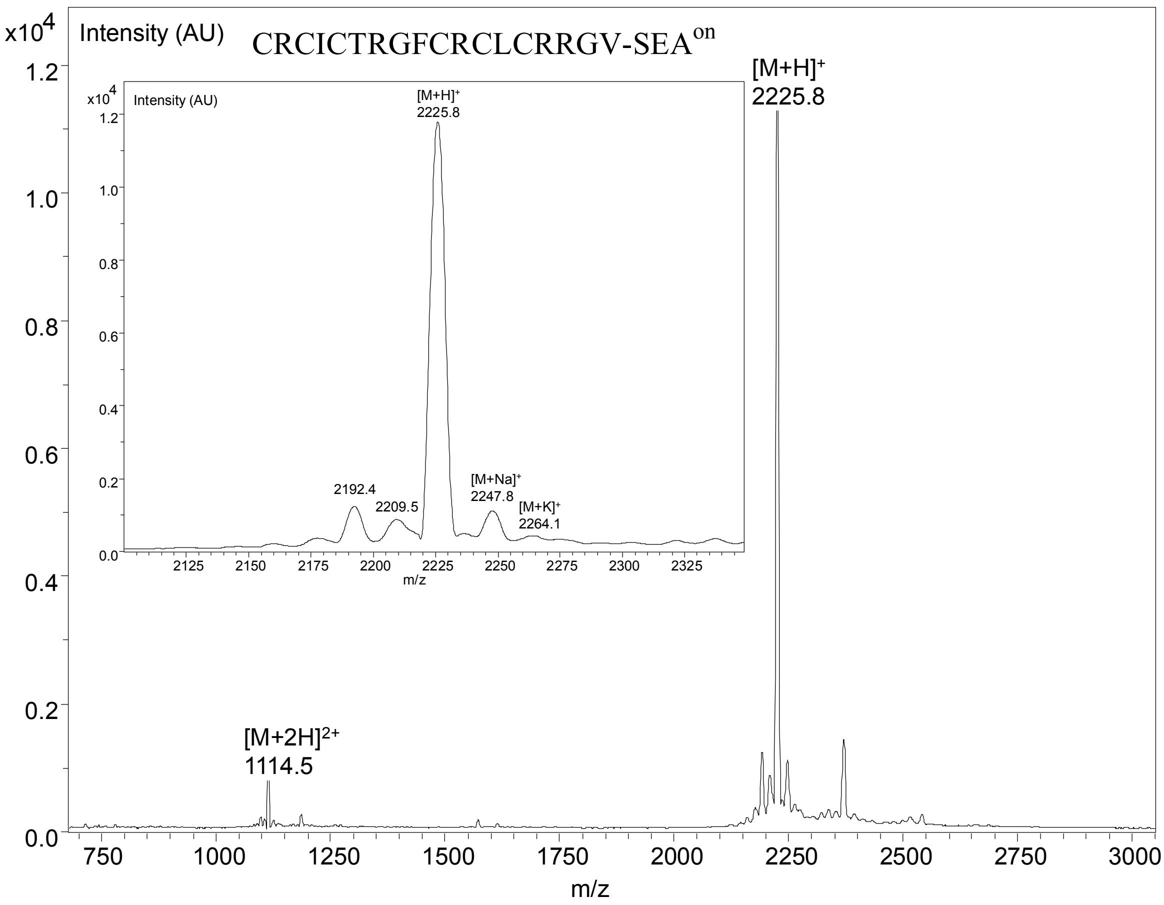

Supplementary Figure 57. MALDI-TOF analysis of peptide 4l. Matrix alpha cyano 4-hydroxycinnaminic acid, positive detection mode, linear mode, $[M+H]^+$ calcd. (mean) 2225.91, found 2225.8.

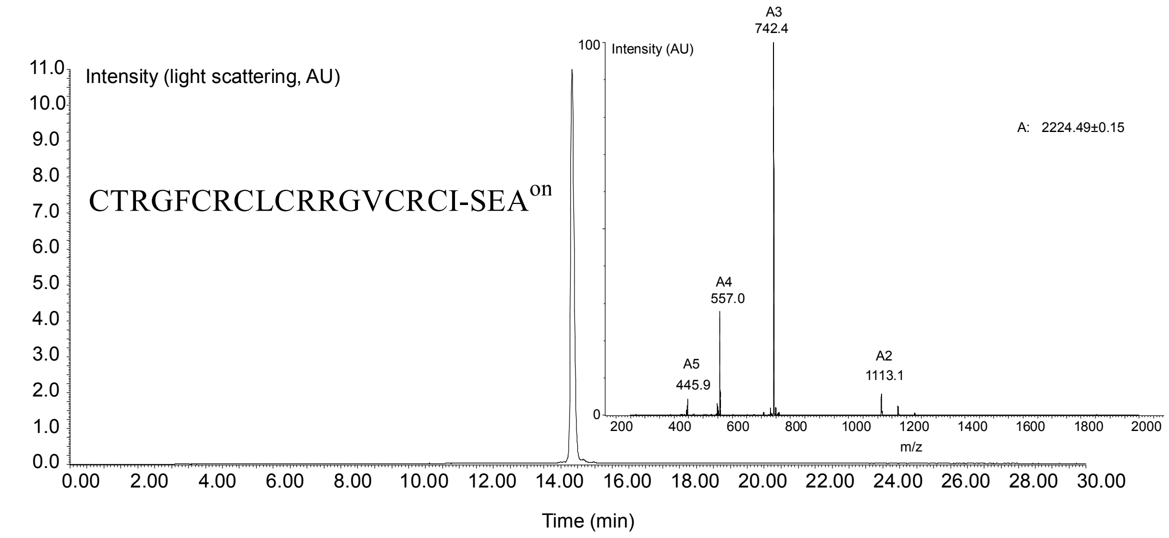

Supplementary Figure 58. LC-MS analysis of peptide 4m. LC trace, eluent A 0.10% TFA in water, eluent B 0.10% TFA in CH_3CN/water: 4/1 by vol. C18 Xbridge BEH 300 Å 5 µm (4.6 × 250 mm) column, gradient 0-100% B in 30 min (1 mL min^{-1}, detection light scattering). MS trace. $[M+2H]^{2+}$ m/z calcd. (monoisotopic) 1112.5, obs 1113.1, $[M+3H]^{3+}$ m/z calcd. (monoisotopic) 742.0, obs 742.4, $[M+4H]^{4+}$ m/z calcd. (monoisotopic) 556.8, obs 557.0, $[M+5H]^{5+}$ m/z calcd. (monoisotopic) 445.6, obs 445.9.

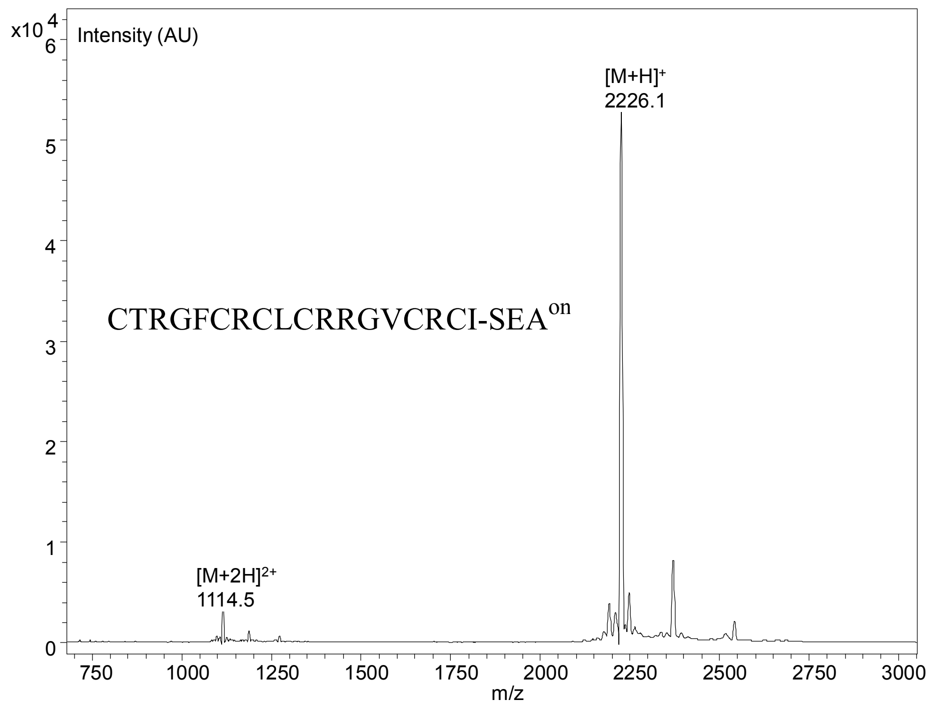

Supplementary Figure 59. MALDI-TOF analysis of peptide 4m. Matrix alpha cyano 4-hydroxycinnaminic acid, positive detection mode, linear mode, $[M+H]^+$ calcd. (mean) 2225.9, found 2226.1.

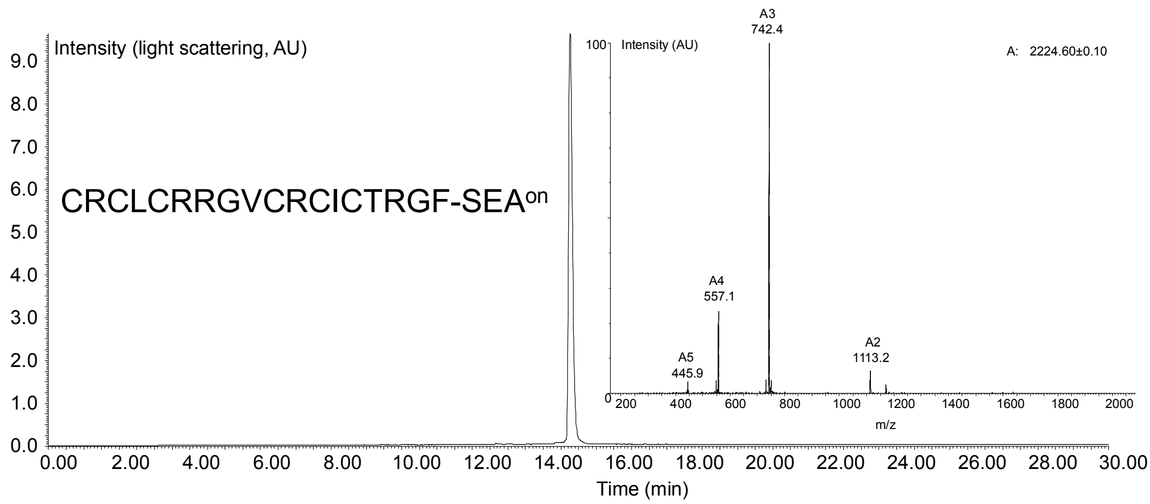

Supplementary Figure 60. LC-MS analysis of peptide 4n. LC trace, eluent A 0.10% TFA in water, eluent B 0.10% TFA in CH_3CN/water: 4/1 by vol. C18 Xbridge BEH 300 Å 5 µm (4.6 × 250 mm) column, gradient 0-100% B in 30 min (1 mL min^{-1}, detection light scattering). MS trace. $[M+2H]^{2+}$ m/z calcd. (monoisotopic) 1112.5, obs 1113.2, $[M+3H]^{3+}$ m/z calcd. (monoisotopic) 742.0, obs 742.4, $[M+4H]^{4+}$ m/z calcd. (monoisotopic) 556.8, obs 557.1, $[M+5H]^{5+}$ m/z calcd. (monoisotopic) 445.6, obs 445.9.

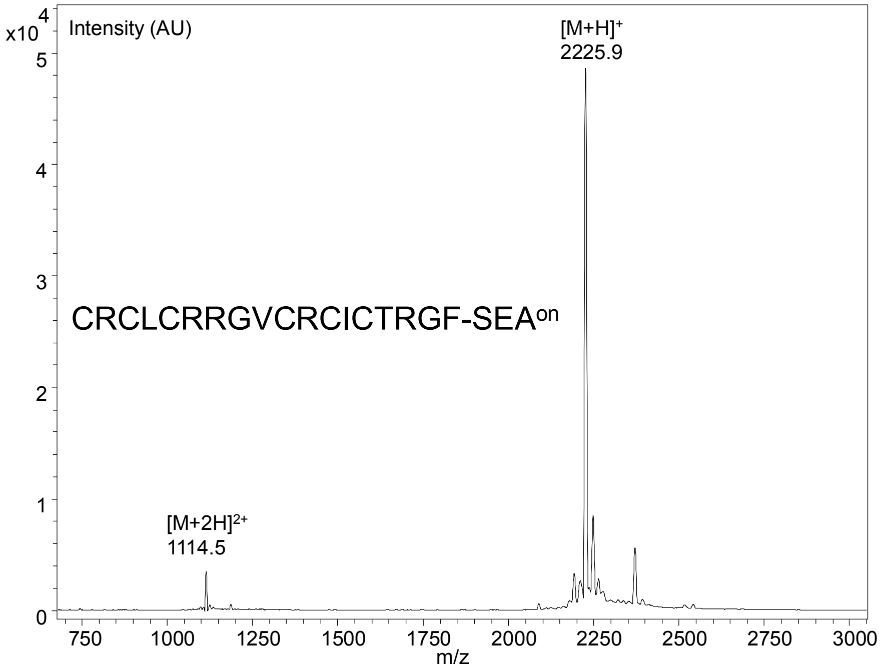

Supplementary Figure 61. MALDI-TOF analysis of peptide 4n. Matrix alpha cyano 4-hydroxycinnaminic acid, positive detection mode, linear mode, $[M+H]^+$ calcd. (mean) 2225.9, found 2225.9**.**

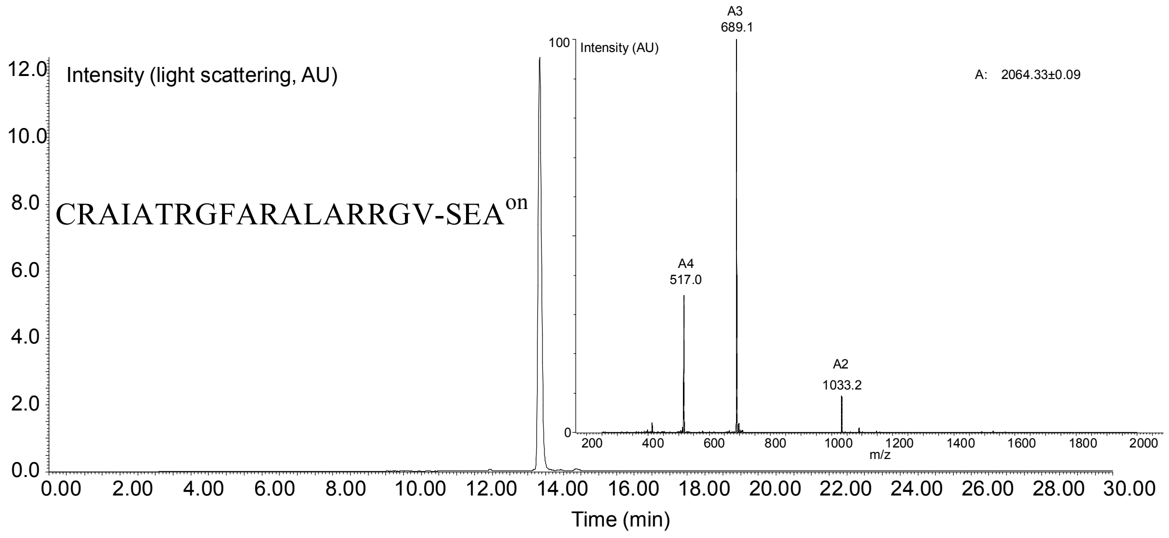

Supplementary Figure 62. LC-MS analysis of peptide 4o. LC trace, eluent A 0.10% TFA in water, eluent B 0.10% TFA in CH_3CN/water: 4/1 by vol. C18 Xbridge BEH 300 Å 5 µm (4.6 × 250 mm) column, gradient 0-100% B in 30 min (1 mL min^{-1}, detection light scattering). MS trace. $[M+2H]^{2+}$ m/z calcd. (monoisotopic) 1032.6, obs 1033.2, $[M+3H]^{3+}$ m/z calcd. (monoisotopic) 688.7, obs 689.1, $[M+4H]^{4+}$ m/z calcd. (monoisotopic) 516.8, obs 517.0.

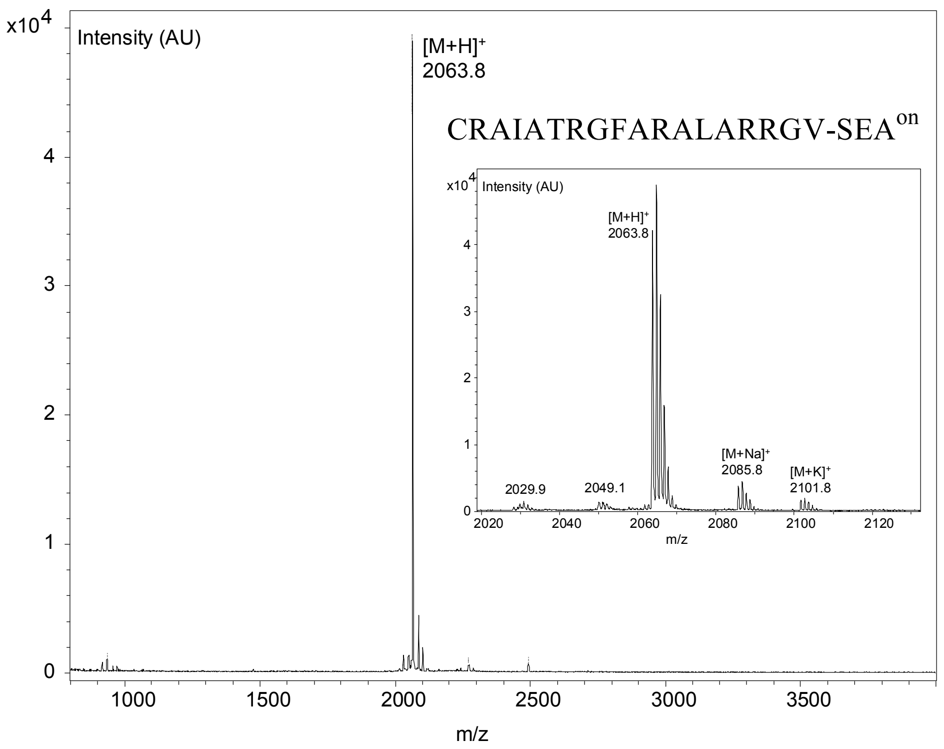

Supplementary Figure 63. MALDI-TOF analysis of peptide 4o. Matrix alpha cyano 4-hydroxycinnaminic acid, positive detection mode, reflected mode, $[M+H]^+$ calcd. (monoisotopic) 2064.1, found 2063.8.

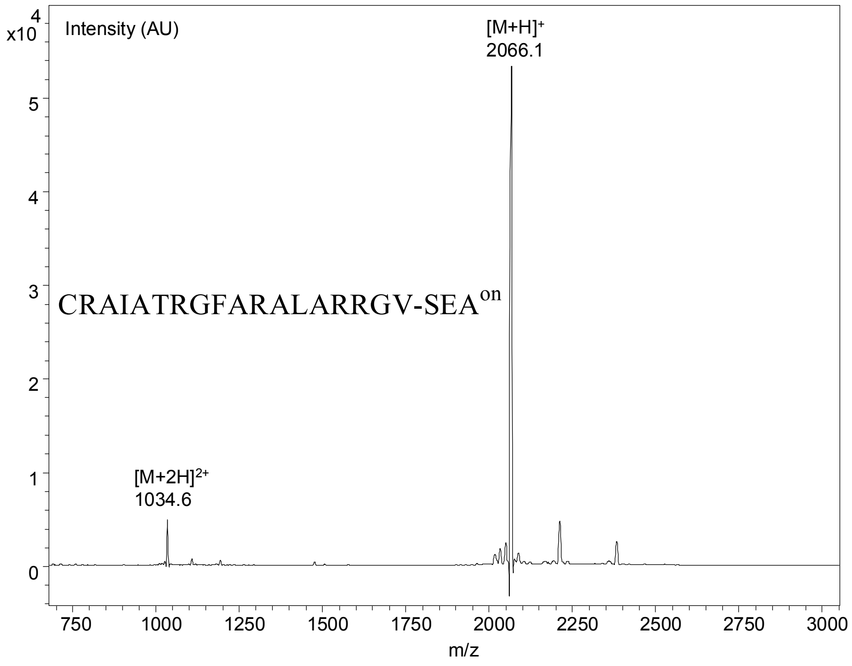

Supplementary Figure 64. MALDI-TOF analysis of peptide 4o. Matrix alpha cyano 4-hydroxycinnaminic acid, positive detection mode, linear mode, $[M+H]^+$ calcd. (mean) 2065.6, found 2066.1.

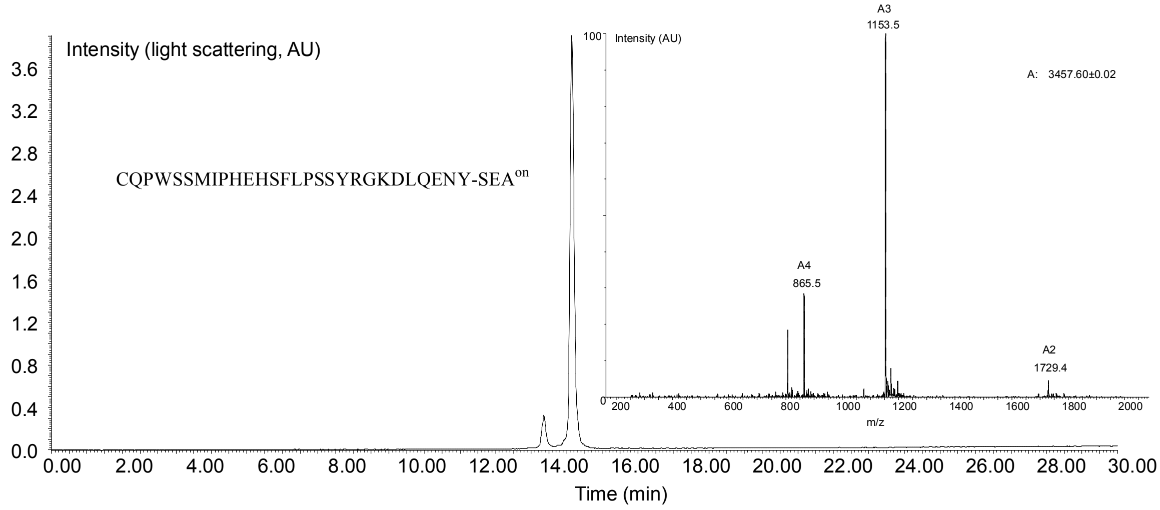

Supplementary Figure 65. LC-MS analysis of peptide 4p. LC trace, eluent A 0.10% TFA in water, eluent B 0.10% TFA in CH_3CN/water: 4/1 by vol. C18 Xbridge BEH 300 Å 5 µm (4.6 × 250 mm) column, gradient 0-100% B in 30 min (1 mL min^{-1}, detection light scattering). MS trace. SEA^{on} $[M+2H]^{2+}$ m/z calcd. (monoisotopic) 1728.3, obs 1729.4, $[M+3H]^{3+}$ m/z calcd. (monoisotopic) 1152.5, obs 1153.5, $[M+4H]^{4+}$ m/z calcd. (monoisotopic) 864.6, obs 865.5.

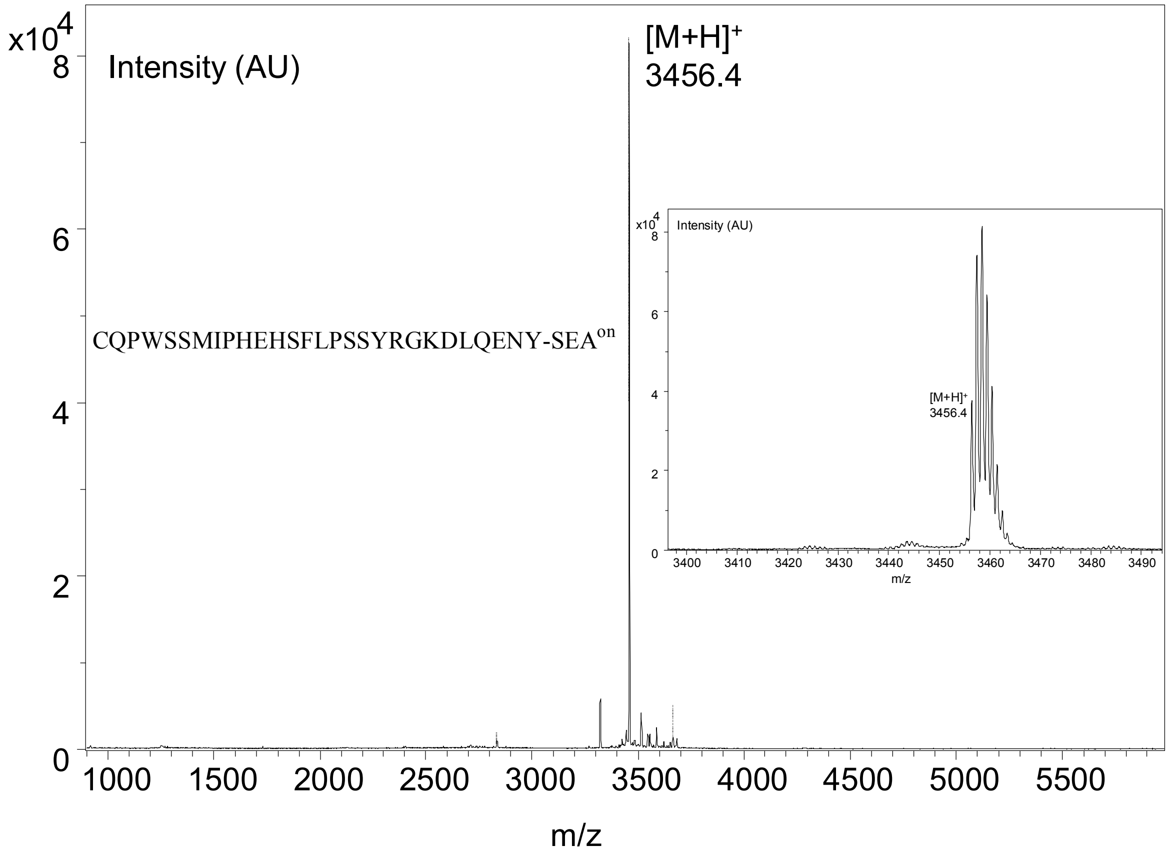

Supplementary Figure 66. MALDI-TOF analysis of peptide 4p. Matrix sinapinic acid, positive detection mode, reflected mode, SEA^{on} $[M+H]^+$ calcd. (monoisotopic) 3455.54, found 3456.4.

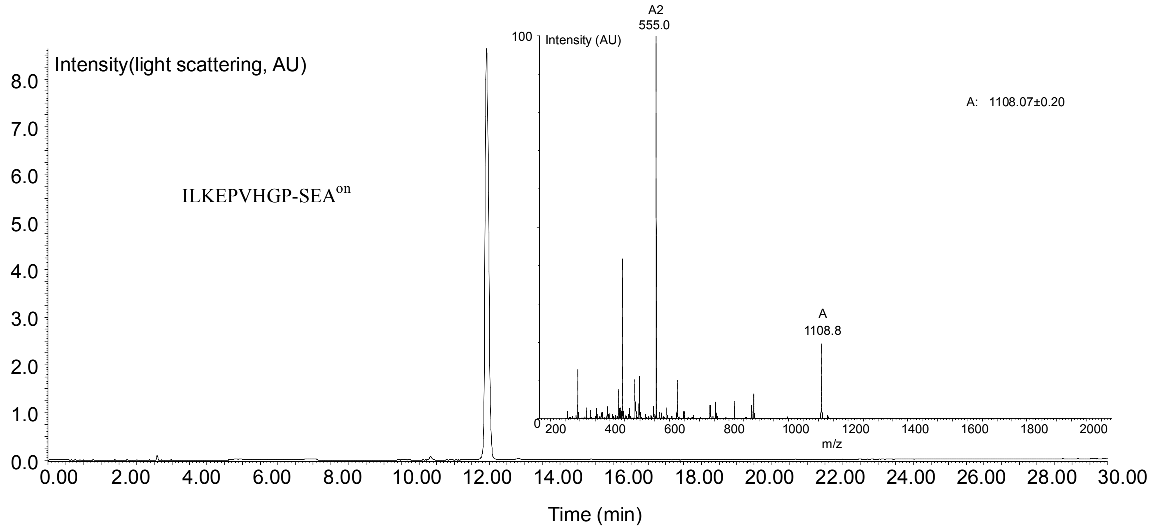

Supplementary Figure 67. LC-MS analysis of peptide 4q. LC trace, eluent A 0.10% TFA in water, eluent B 0.10% TFA in CH_3CN/water: 4/1 by vol. C18 Xbridge BEH 300 Å 5 µm (4.6 × 250 mm) column, gradient 0-100% B in 30 min (1 mL min^{-1}, light scattering detection). MS trace. $[M+H]^+$ m/z calcd. (monoisotopic) 1108.6, obs 1108.8.

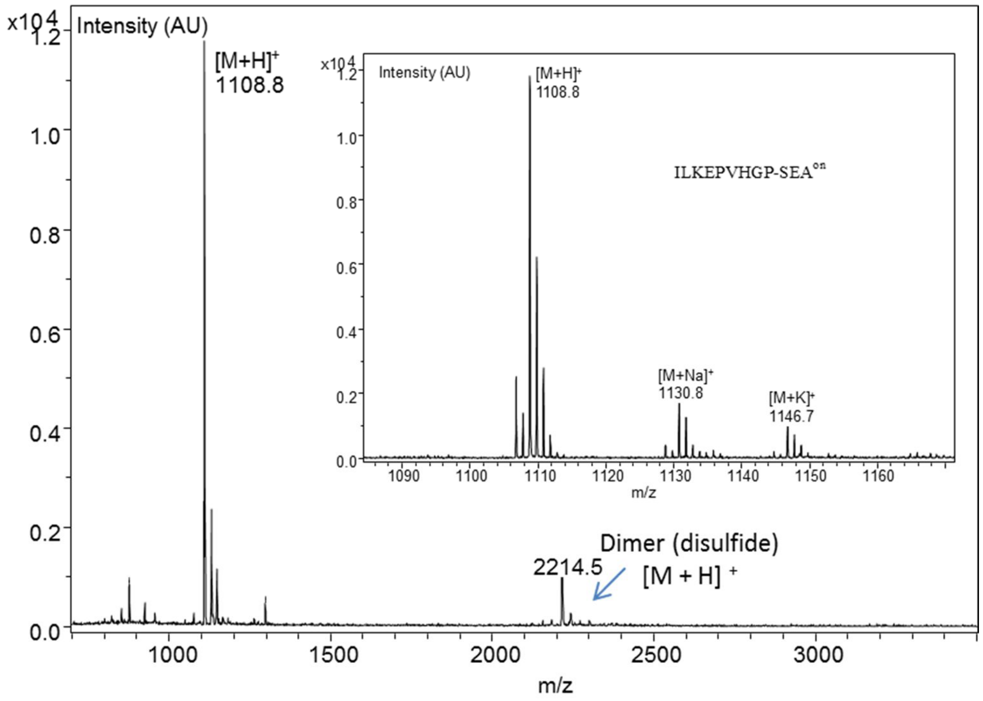

Supplementary Figure 68. MALDI-TOF analysis of peptide 4q. Matrix alpha cyano 4-hydroxycinnaminic acid, positive detection mode, $[M+H]^+$ calcd. (monoisotopic) 1108.6, found 1108.8.

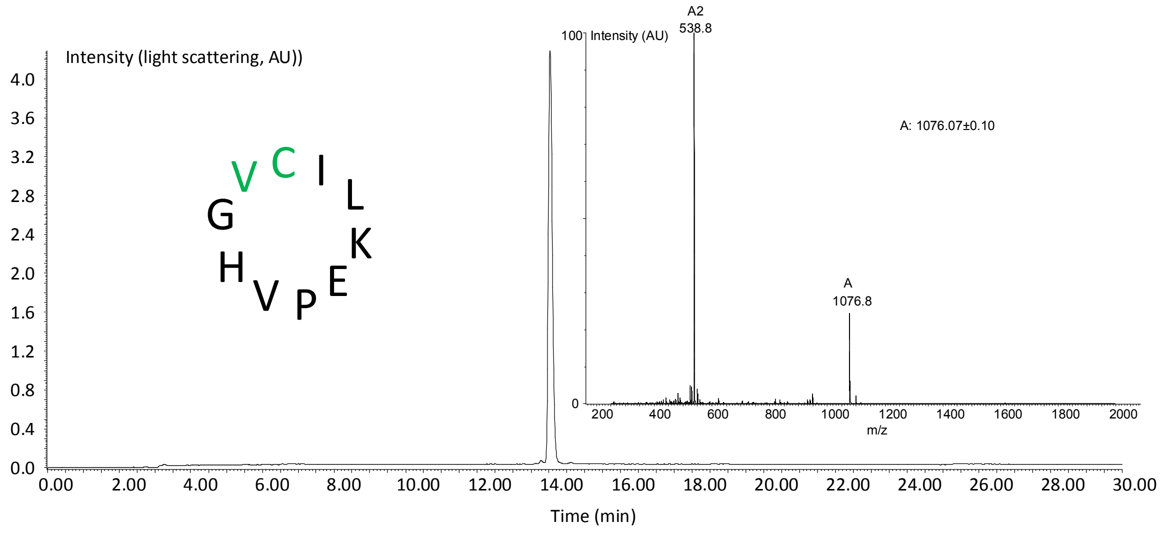

Supplementary Figure 69. LC-MS analysis of peptide 7c. LC trace, eluent A 0.10% TFA in water, eluent B 0.10% TFA in CH_3CN/water: 4/1 by vol. C18 Xbridge BEH 300 Å 5 µm (4.6 × 250 mm) column, gradient 0-100% B in 30 min (1 mL min^{-1}, detection light scattering). MS trace. $[M+H]^+$ m/z calcd. (monoisotopic) 1076.6, obs 1076.8, $[M+2H]^{2+}$ m/z calcd. (monoisotopic) 538.8, obs 538.8.

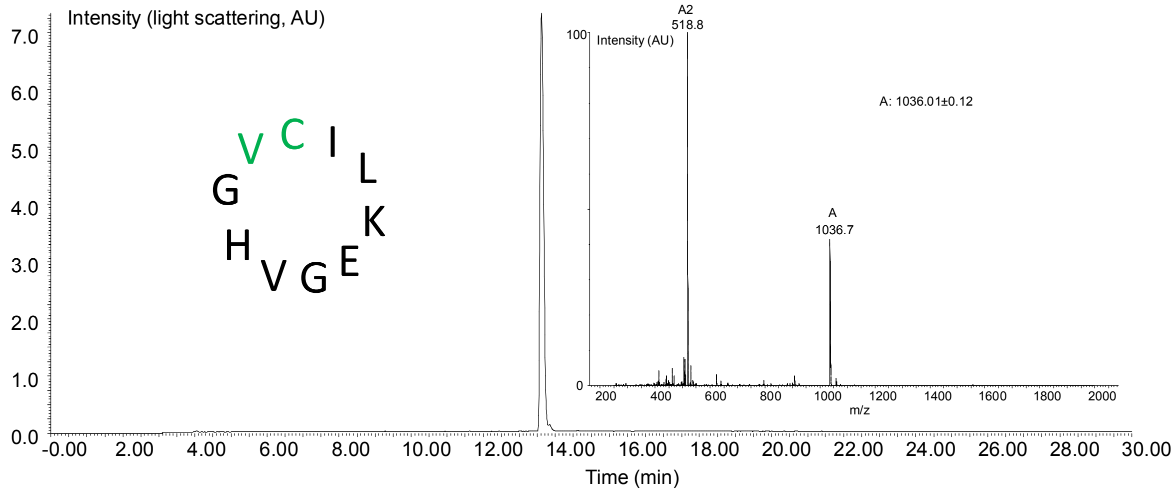

Supplementary Figure 70. LC-MS analysis of peptide 7d. LC trace, eluent A 0.10% TFA in water, eluent B 0.10% TFA in CH_3CN/water: 4/1 by vol. C18 Xbridge BEH 300 Å 5 µm (4.6 × 250 mm) column, gradient 0-100% B in 30 min (1 mL min^{-1}, detection light scattering). MS trace. $[M+H]^+$ m/z calcd. (monoisotopic) 1036.6, obs 1036.7, $[M+2H]^{2+}$ m/z calcd. (monoisotopic) 518.8, obs 518.8.

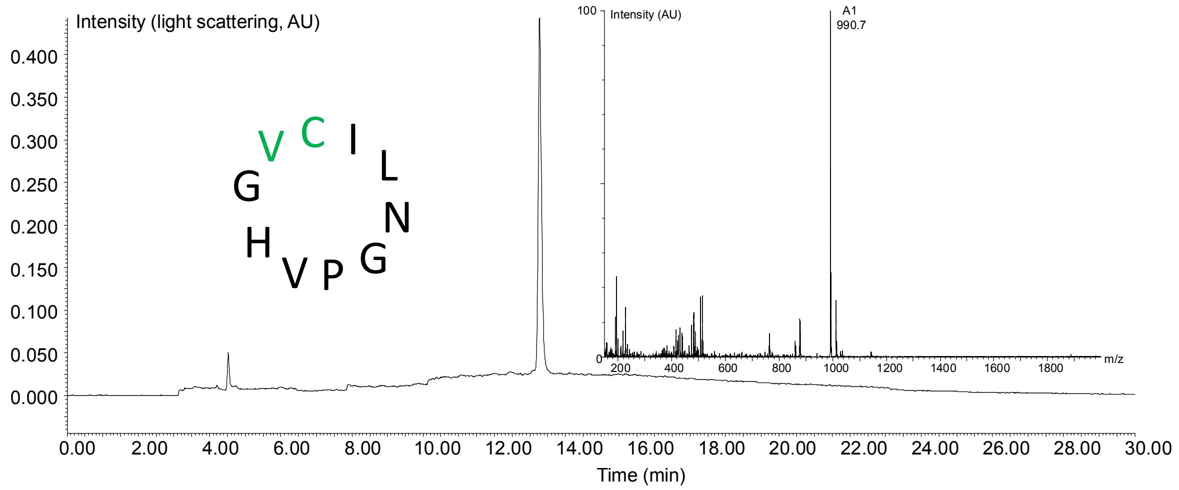

Supplementary Figure 71. LC-MS analysis of peptide 7e. LC trace, eluent A 0.10% TFA in water, eluent B 0.10% TFA in CH_3CN/water: 4/1 by vol. C18 Xbridge BEH 300 Å 5 µm (4.6 × 250 mm) column, gradient 0-100% B in 30 min (1 mL min^{-1}, detection light scattering). MS trace. $[M+H]^+$ m/z calcd. (monoisotopic) 990.5, obs 990.7.

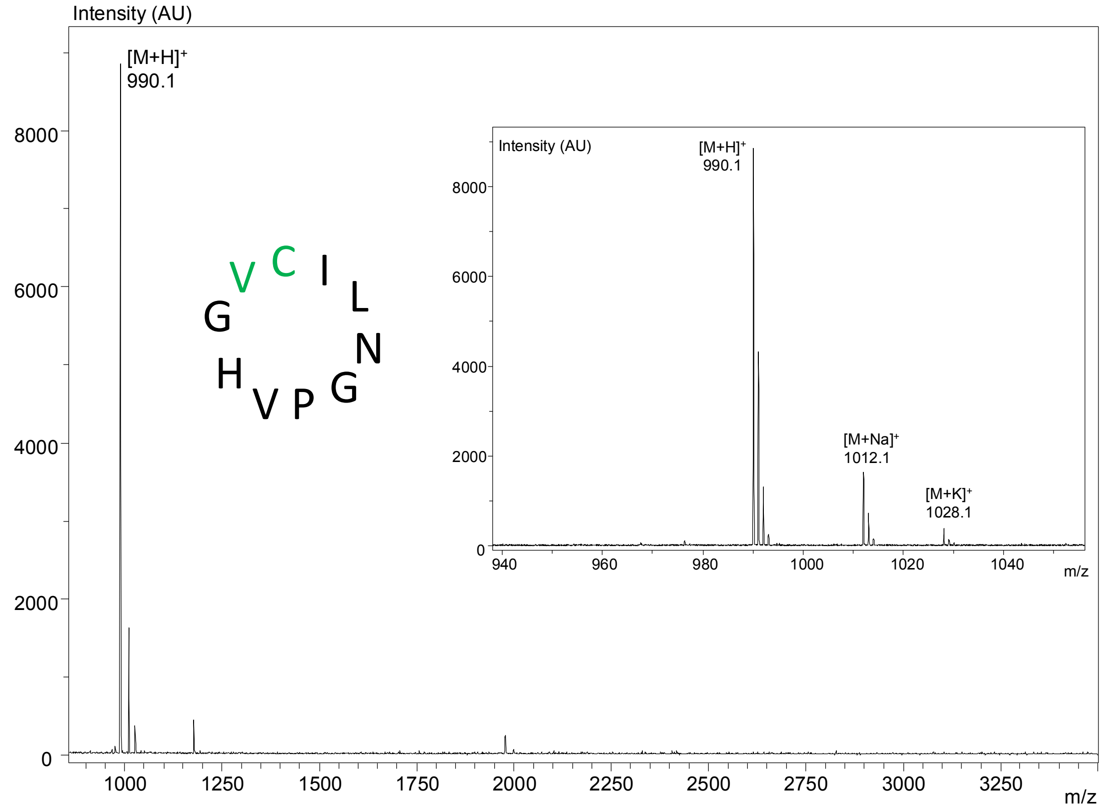

Supplementary Figure 72. MALDI-TOF analysis of peptide 7e. Matrix alpha cyano 4-hydroxycinnaminic acid, positive detection mode, $[M+H]^+$ calcd. (monoisotopic) 990.5, found 990.1

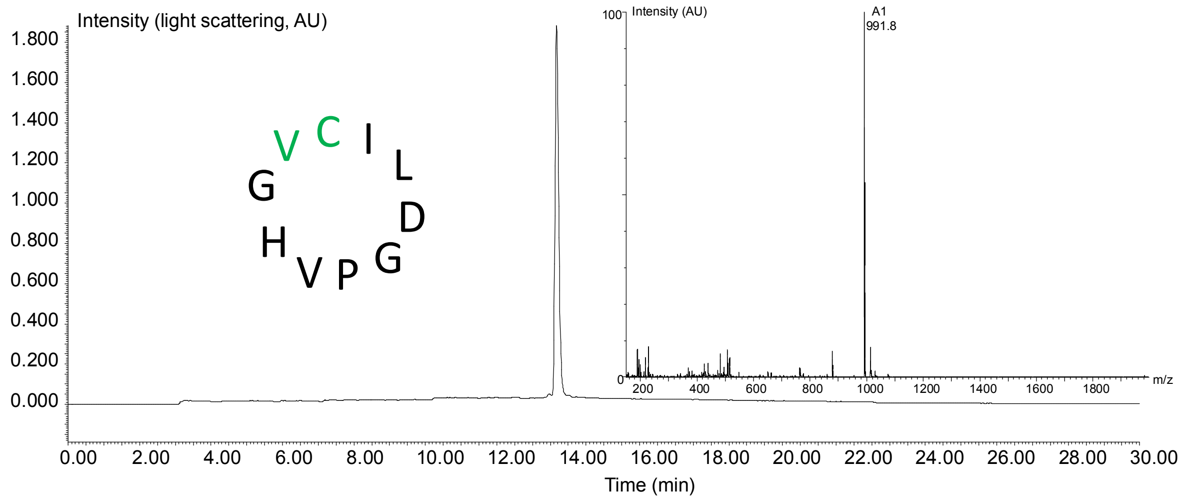

Supplementary Figure 73. LC-MS analysis of peptide 7f. LC trace, eluent A 0.10% TFA in water, eluent B 0.10% TFA in CH_3CN/water: 4/1 by vol. C18 Xbridge BEH 300 Å 5 µm (4.6 × 250 mm) column, gradient 0-100% B in 30 min (1 mL min-1, detection light scattering). MS trace. [M+H]+ m/z calcd. (monoisotopic) 991.5, obs 991.8.

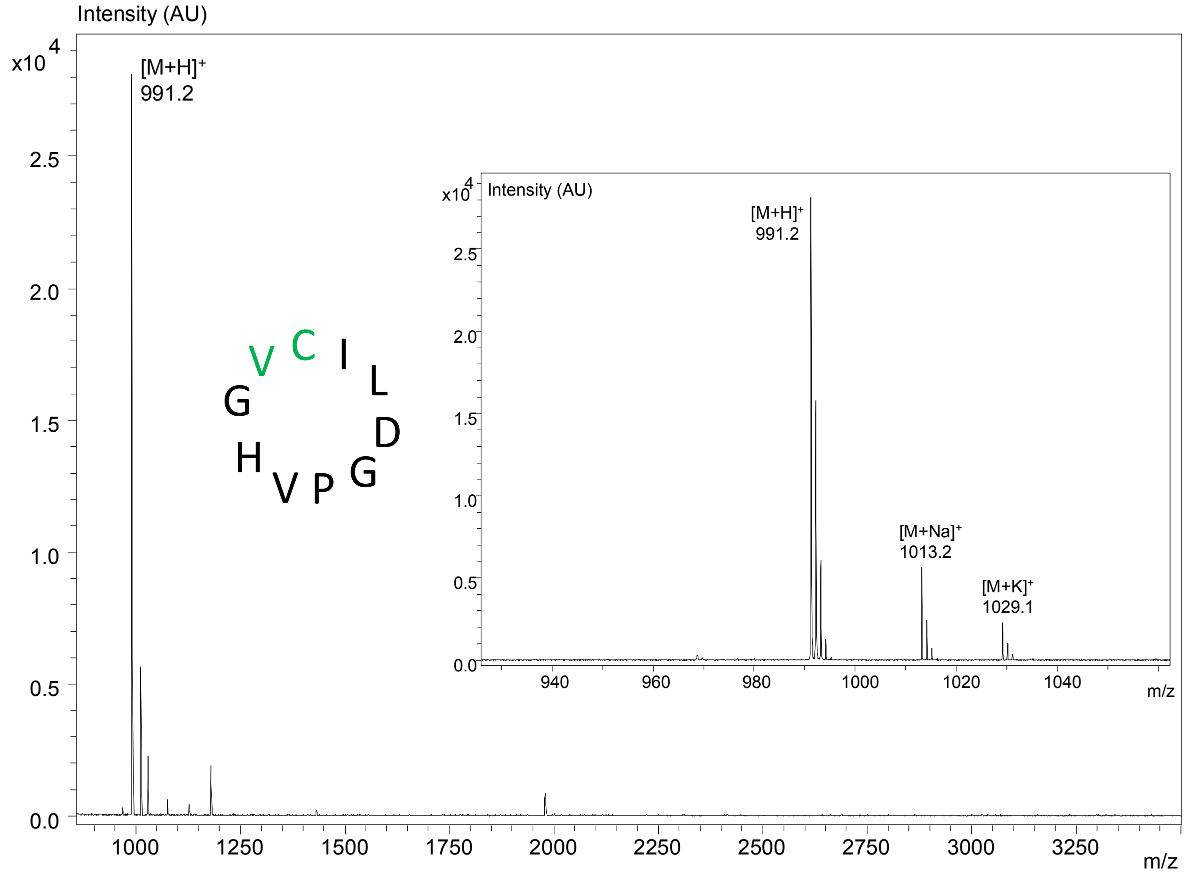

Supplementary Figure 74. MALDI-TOF analysis of peptide 7f. Matrix alpha cyano 4-hydroxycinnaminic acid, positive detection mode, $[M+H]^+$ calcd. (monoisotopic) 991.5, found 991.2.

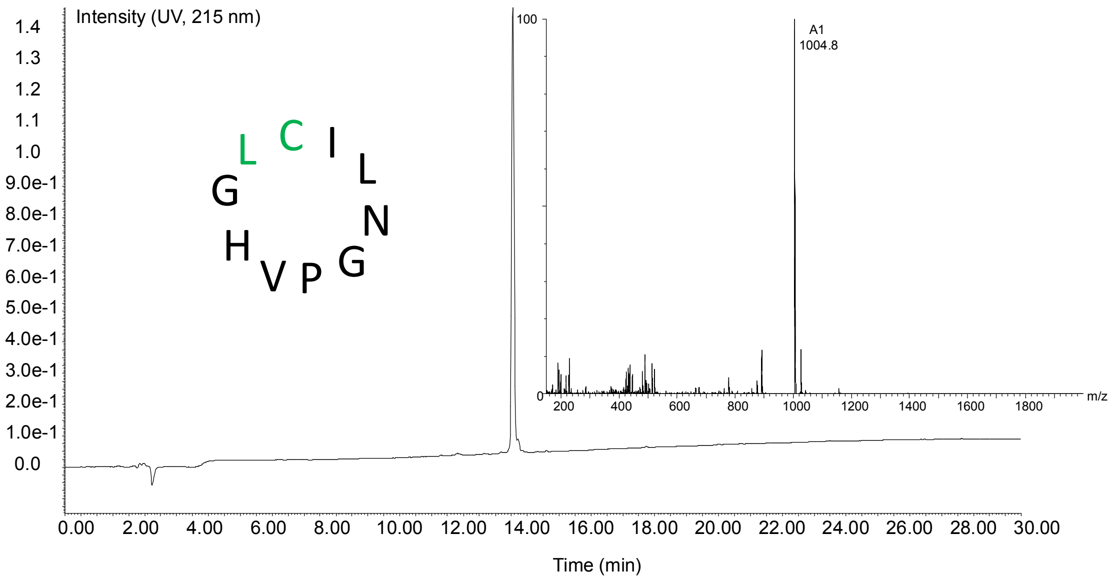

Supplementary Figure 75. LC-MS analysis of peptide 7g. LC trace, eluent A 0.10% TFA in water, eluent B 0.10% TFA in CH_3CN/water: 4/1 by vol. C18 Xbridge BEH 300 Å 5 µm (4.6 × 250 mm) column, gradient 0-100% B in 30 min (1 mL min^{-1}, detection UV 215 nm). MS trace. $[M+H]^+$ m/z calcd. (monoisotopic) 1004.5, obs 1004.8.

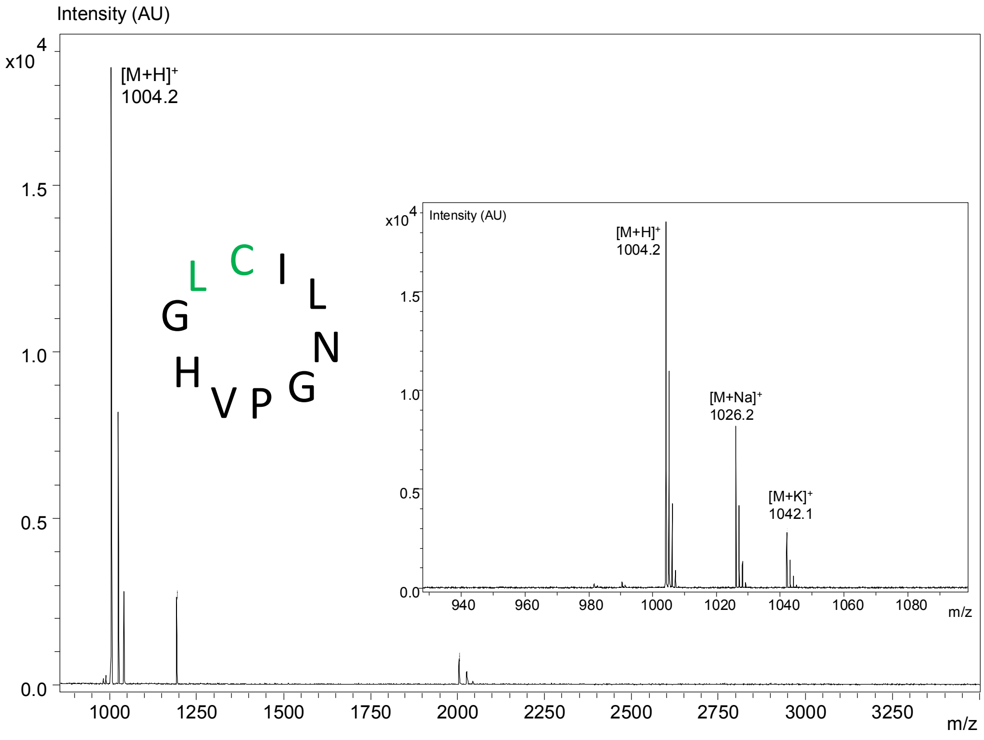

Supplementary Figure 76. MALDI-TOF analysis of peptide 7g. Matrix alpha cyano 4-hydroxycinnaminic acid, positive detection mode, $[M+H]^+$ calcd. (monoisotopic) 1004.5, found 1004.2.

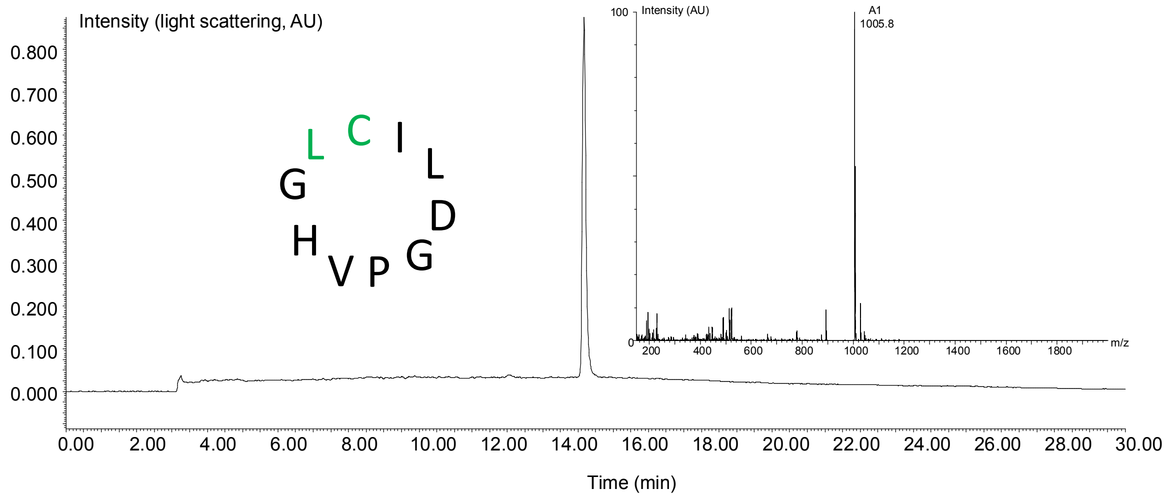

Supplementary Figure 77. LC-MS analysis of peptide 7h. LC trace, eluent A 0.10% TFA in water, eluent B 0.10% TFA in CH_3CN/water: 4/1 by vol. C18 Xbridge BEH 300 Å 5 µm (4.6 × 250 mm) column, gradient 0-100% B in 30 min (1 mL min^{-1}, detection light scattering). MS trace. $[M+H]^+$ m/z calcd. (monoisotopic) 1005.5, obs 1005.8.

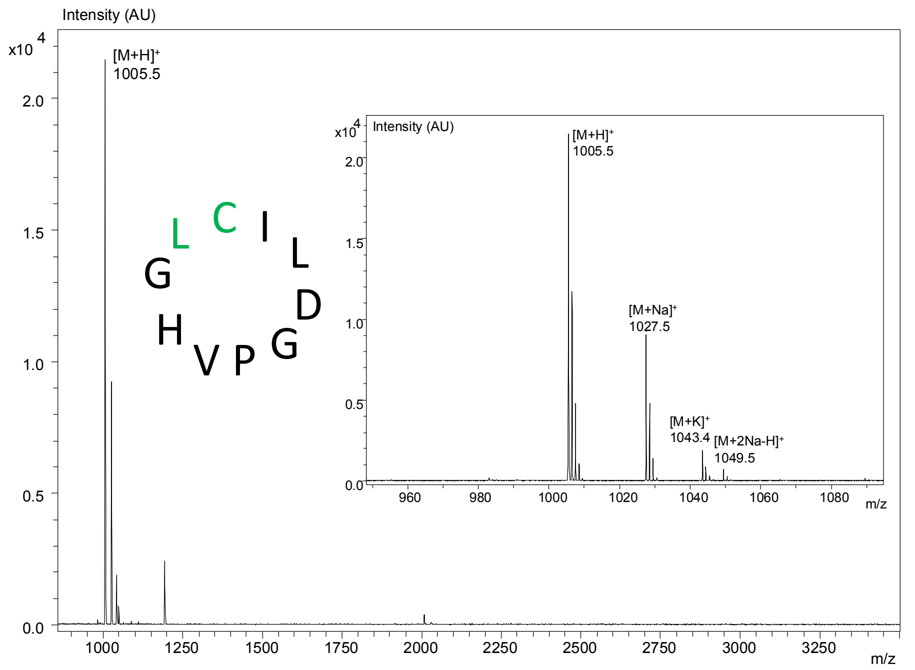

Supplementary Figure 78. MALDI-TOF analysis of peptide 7h. Matrix alpha cyano 4-hydroxycinnaminic acid, positive detection mode, $[M+H]^+$ calcd. (monoisotopic) 1005.5, found 1005.5.

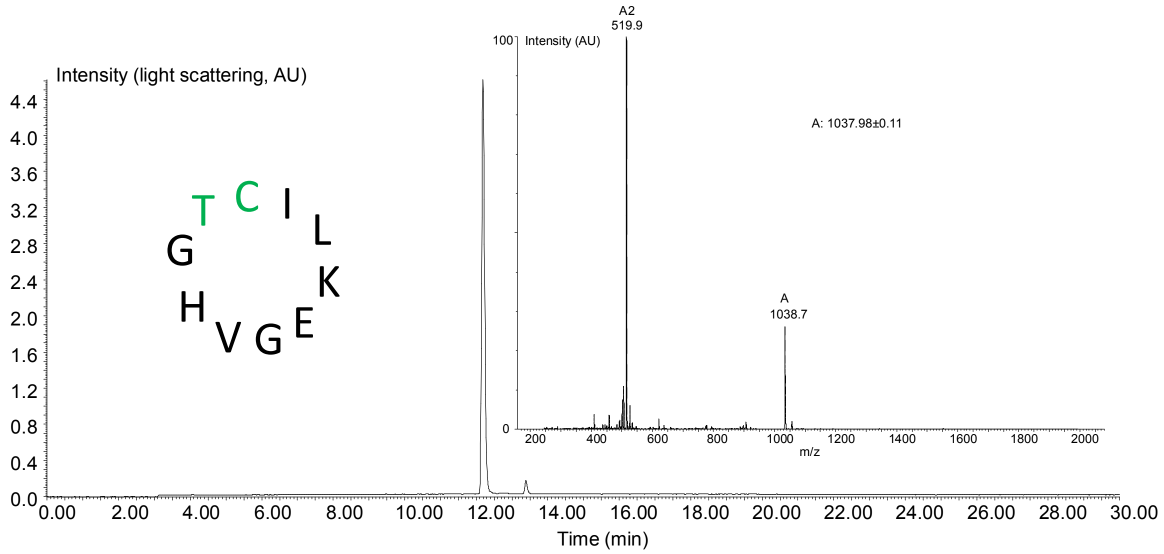

Supplementary Figure 79. LC-MS analysis of peptide 7i. LC trace, eluent A 0.10% TFA in water, eluent B 0.10% TFA in CH_3CN/water: 4/1 by vol. C18 Xbridge BEH 300 Å 5 µm (4.6 × 250 mm) column, gradient 0-100% B in 30 min (1 mL min^{-1}, detection light scattering). MS trace. $[M+H]^+$ m/z calcd. (monoisotopic) 1038.6, obs 1038.7, $[M+2H]^{2+}$ m/z calcd. (monoisotopic) 519.8, obs 519.9.

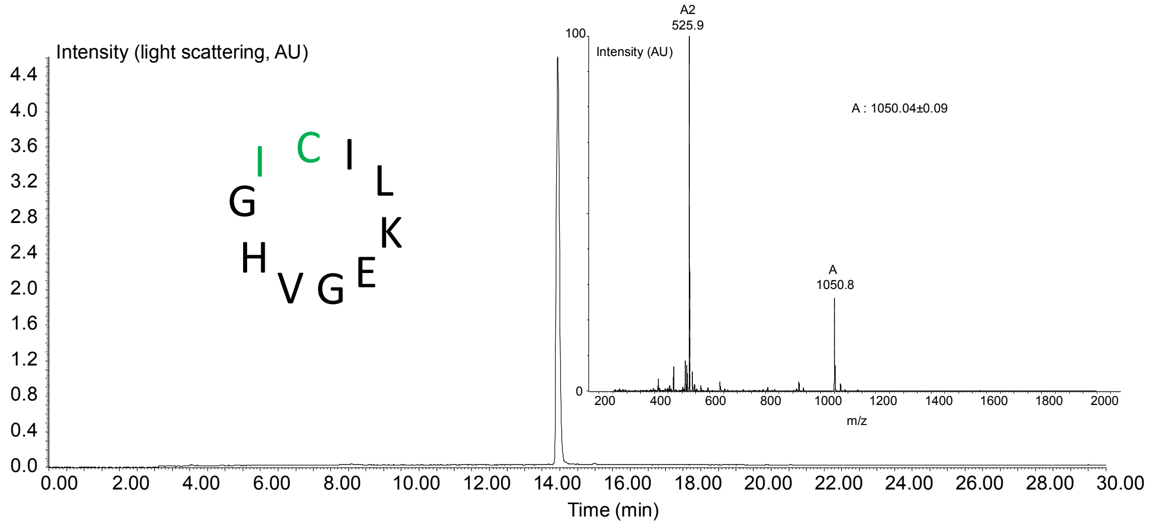

Supplementary Figure 80. LC-MS analysis of peptide 7j. LC trace, eluent A 0.10% TFA in water, eluent B 0.10% TFA in CH_3CN/water: 4/1 by vol. C18 Xbridge BEH 300 Å 5 µm (4.6 × 250 mm) column, gradient 0-100% B in 30 min (1 mL min^{-1}, detection light scattering). MS trace. $[M+H]^+$ m/z calcd. (monoisotopic) 1050.6, obs 1050.8, $[M+2H]^{2+}$ m/z calcd. (monoisotopic) 525.8, obs 525.9.

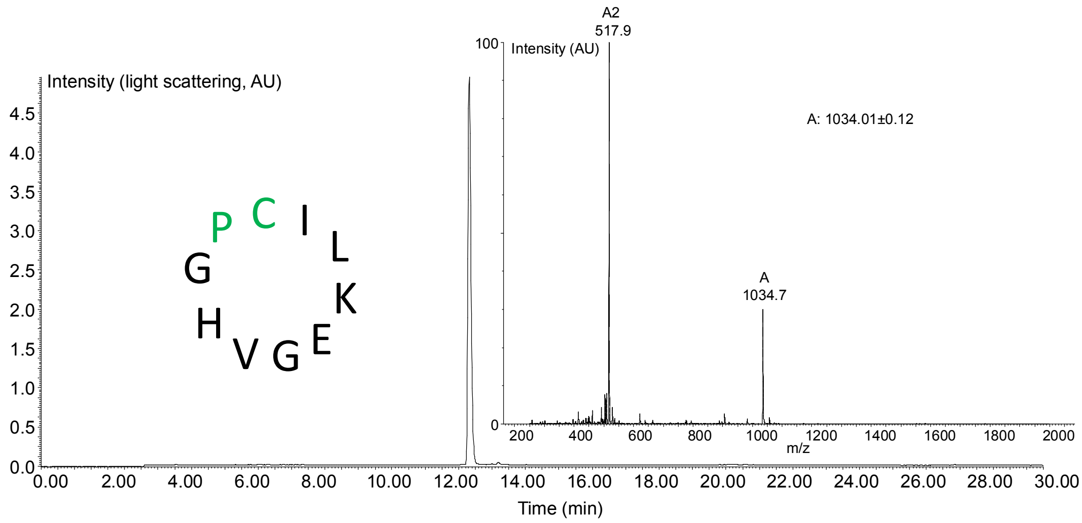

Supplementary Figure 81. LC-MS analysis of peptide 7k. LC trace, eluent A 0.10% TFA in water, eluent B 0.10% TFA in CH_3CN/water: 4/1 by vol. C18 Xbridge BEH 300 Å 5 µm (4.6 × 250 mm) column, gradient 0-100% B in 30 min (1 mL min^{-1}, detection light scattering). MS trace. $[M+H]^+$ m/z calcd. (monoisotopic) 1034.6, obs 1034.7, $[M+2H]^{2+}$ m/z calcd. (monoisotopic) 517.8, obs 517.9.

a)

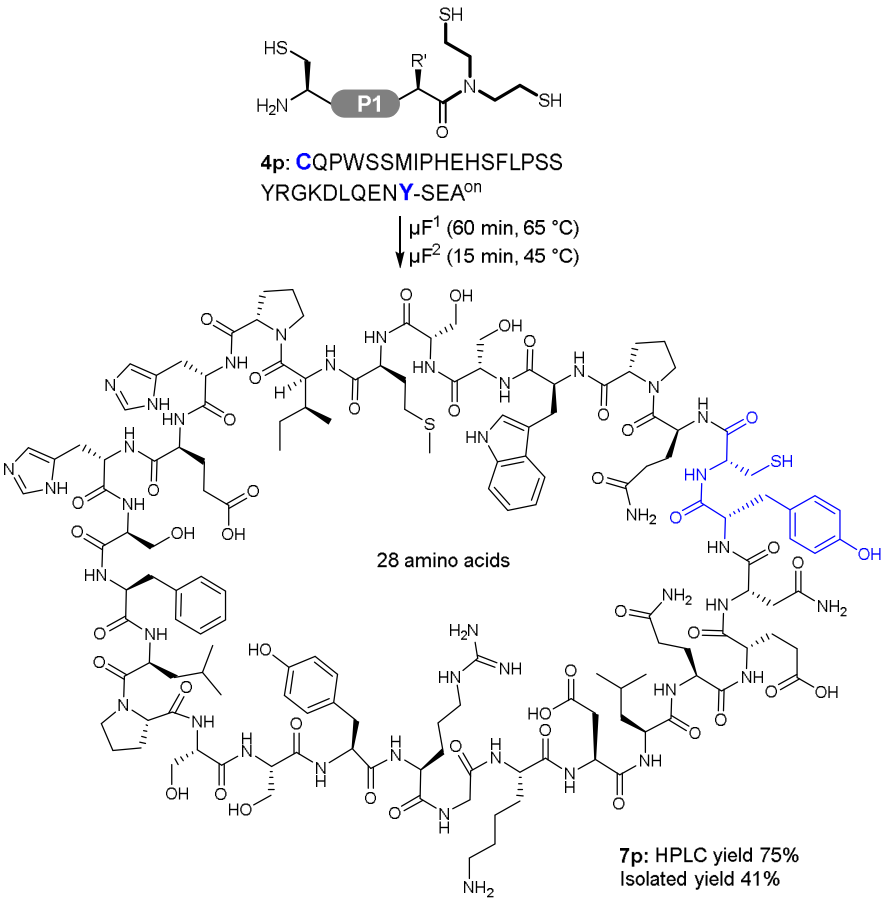

b)

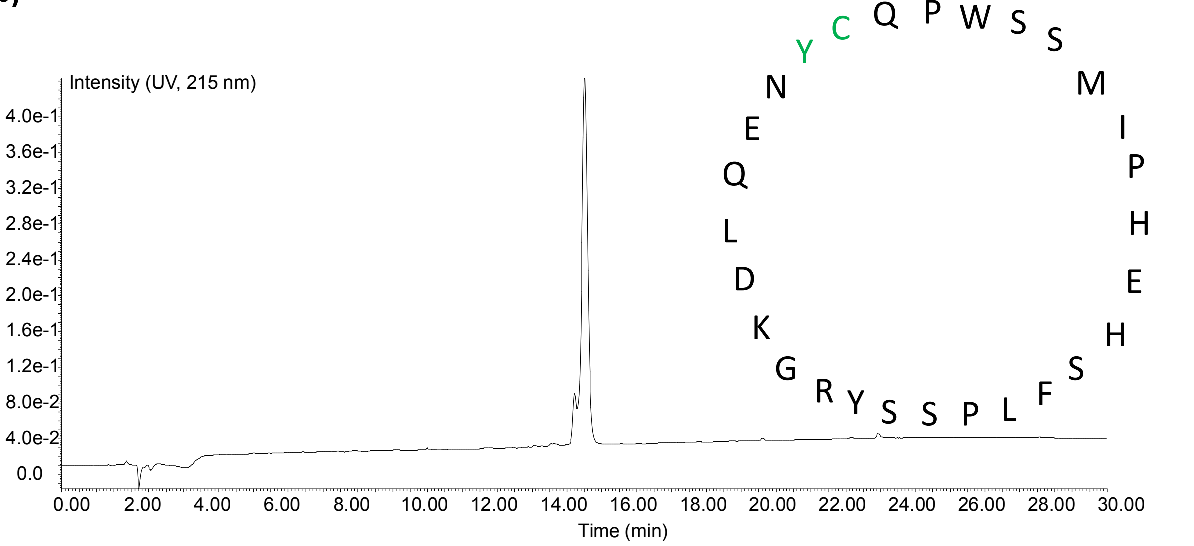

Supplementary Figure 82. a) Synthesis of cyclic peptide 7p. b) RP-HPLC analysis of peptide 7p. RP-HPLC trace, eluent A 0.10% TFA in water, eluent B 0.10% TFA in CH_3CN/water: 4/1 by vol. C18 Xbridge BEH 300 Å 5 µm (4.6 × 250 mm) column, gradient 0-100% B in 30 min (1 mL min^{-1}, detection UV 215 nm).

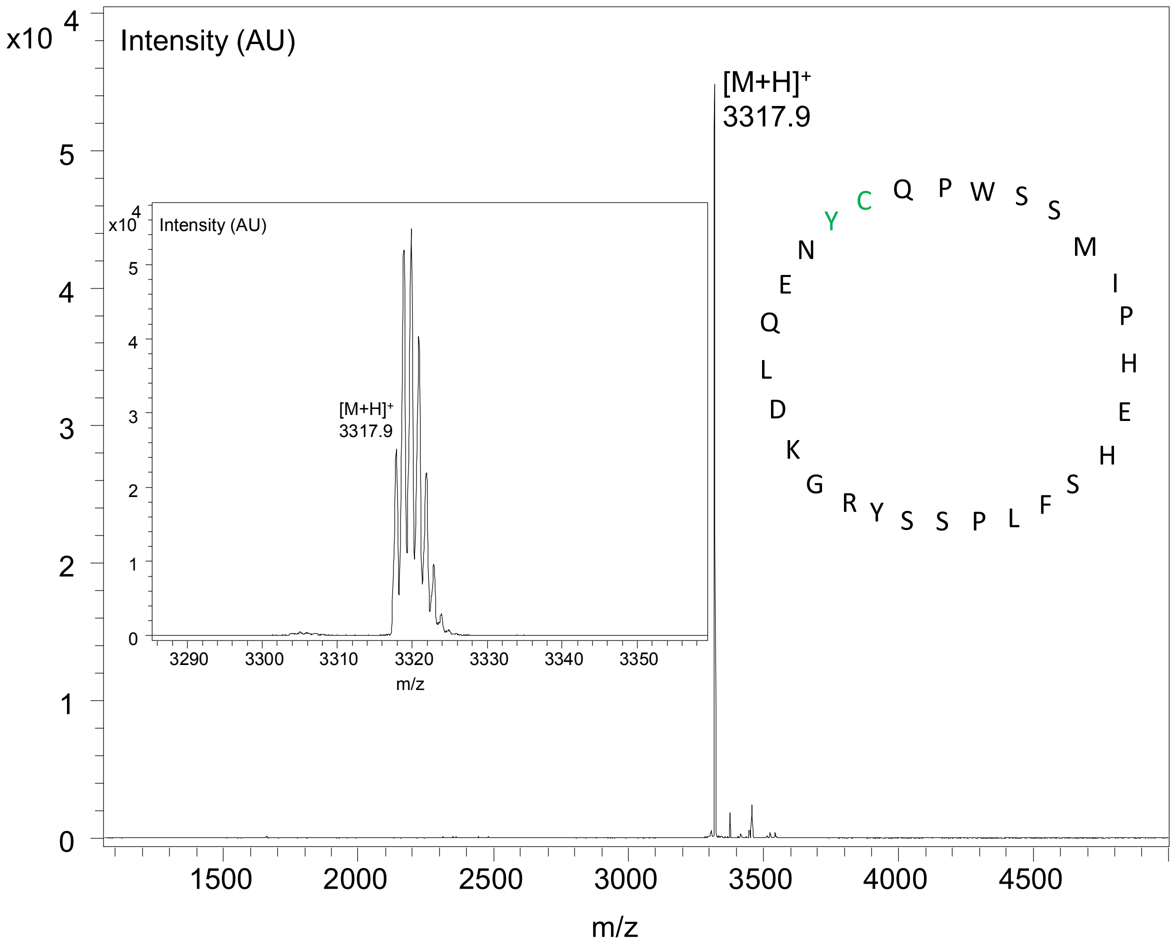

Supplementary Figure 83. MALDI-TOF analysis of peptide 7p. Matrix sinapinic acid, positive detection mode, reflector mode, $[M+H]^+$ calcd. (monoisotopic) 3318.51, found 3317.9.

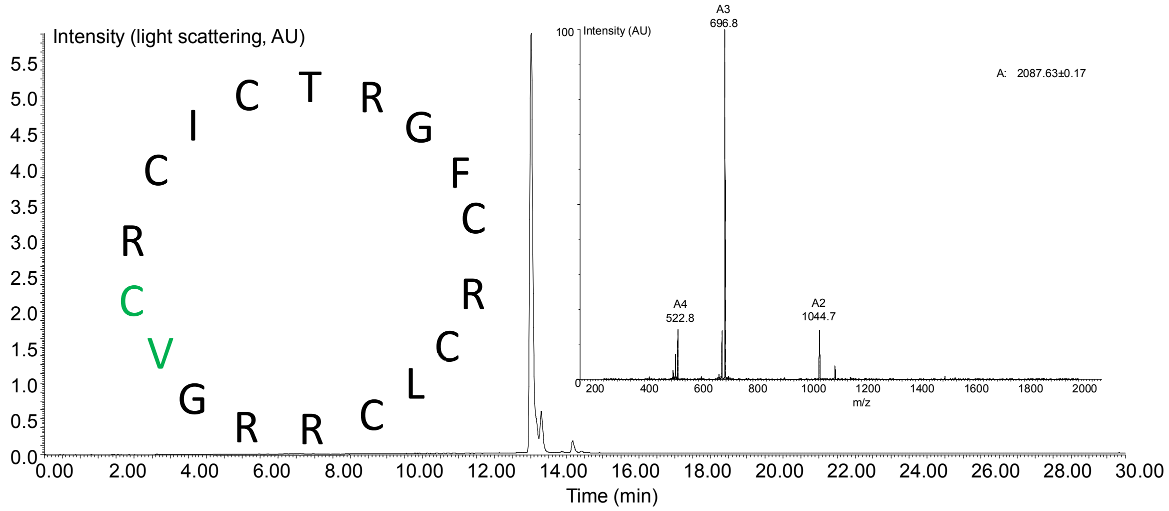

Supplementary Figure 84. LC-MS analysis of peptide RTD-1 peptide produced from 4l. LC trace, eluent A 0.10% TFA in water, eluent B 0.10% TFA in CH_3CN/water: 4/1 by vol. C18 Xbridge BEH 300 Å 5 µm (4.6 × 250 mm) column, gradient 0-100% B in 30 min (1 mL min^{-1}, detection light scattering). MS trace. $[M+2H]^{2+}$ m/z calcd. (monoisotopic) 1044.0, obs 1044.7, $[M+3H]^{3+}$ m/z calcd. (monoisotopic) 696.3, obs 696.8, $[M+4H]^{4+}$ m/z calcd. (monoisotopic) 522.5, obs 522.8.

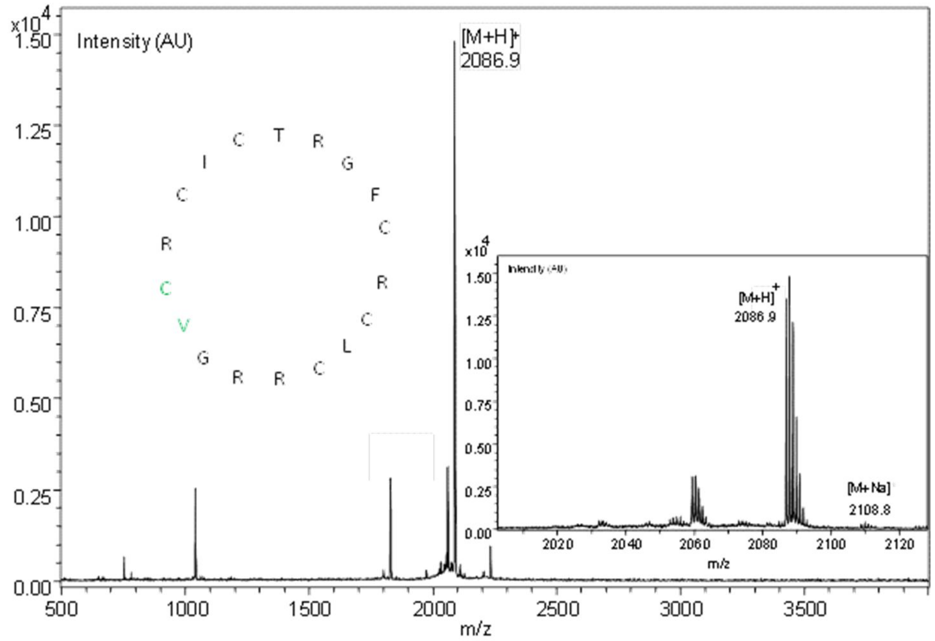

Supplementary Figure 85. MALDI-TOF analysis of RTD-1 peptide produced from 4l. Matrix alpha cyano 4-hydroxycinnaminic acid, positive detection mode, $[M+H]^+$ calcd. (monoisotopic) 2087.0, found 2086.9.

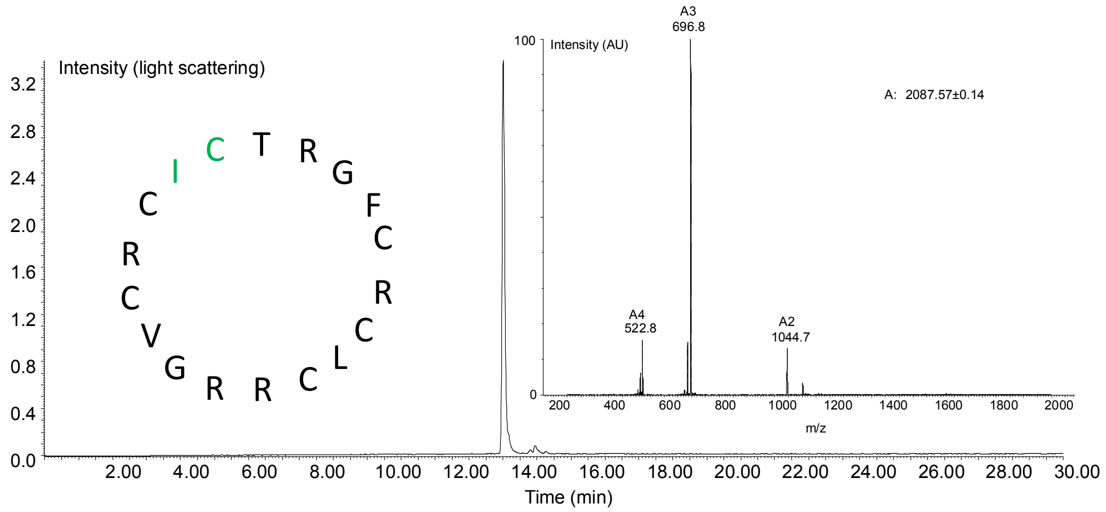

Supplementary Figure 86. LC-MS analysis of RTD-1 peptide produced from 4m. LC trace, eluent A 0.10% TFA in water, eluent B 0.10% TFA in CH_3CN/water: 4/1 by vol. C18 Xbridge BEH 300 Å 5 µm (4.6 × 250 mm) column, gradient 0-100% B in 30 min (1 mL min^{-1}, detection light scattering). MS trace. $[M+2H]^{2+}$ m/z calcd. (monoisotopic) 1044.0, obs 1044.7, $[M+3H]^{3+}$ m/z calcd. (monoisotopic) 696.3, obs 696.8, $[M+4H]^{4+}$ m/z calcd. (monoisotopic) 522.5, obs 522.8.

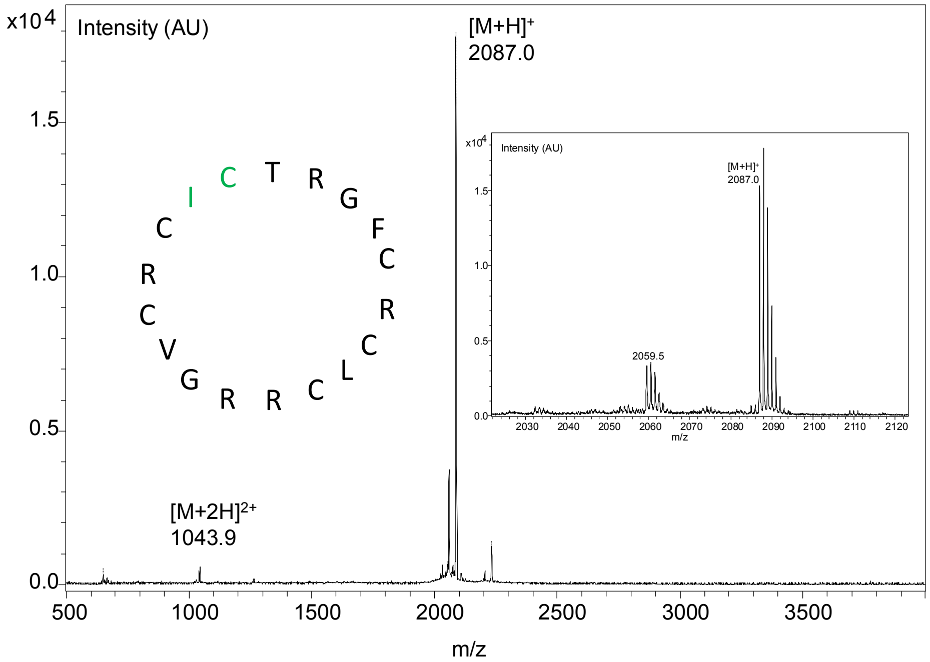

Supplementary Figure 87. MALDI-TOF analysis of RTD-1 peptide produced from 4m. Matrix alpha cyano 4-hydroxycinnaminic acid, positive detection mode, reflector mode $[M+H]^+$ calcd. (monoisotopic) 2087.0, found 2087.0, $[M+2H]^{2+}$ calcd. (monoisotopic) 1044.0, found 1043.9.

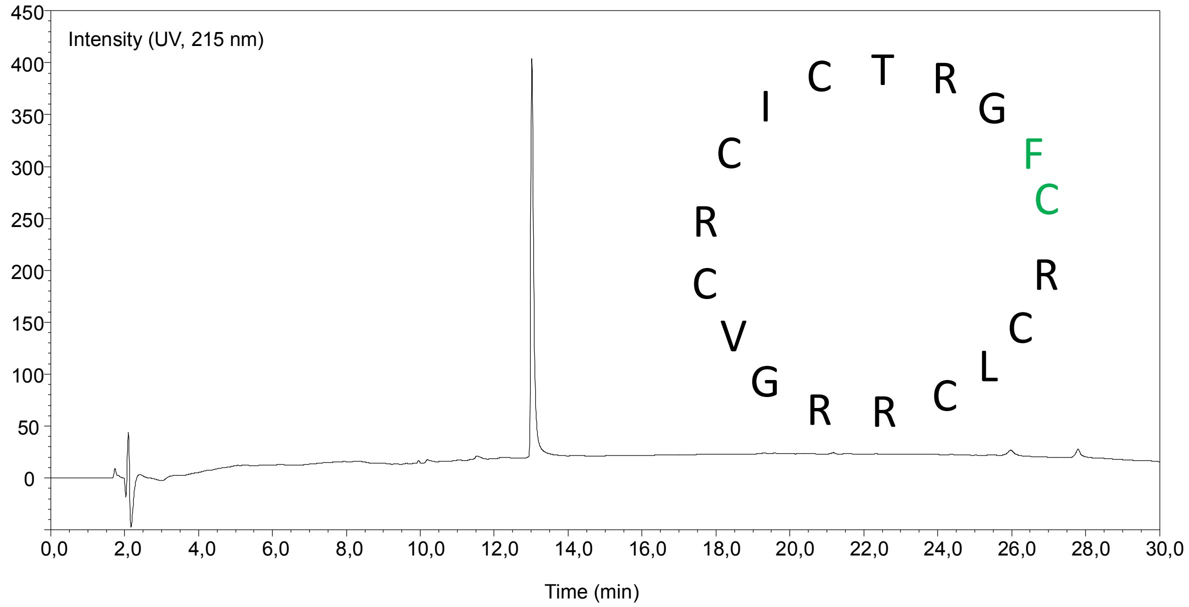

Supplementary Figure 88. RP-HPLC analysis of RTD-1 peptide produced from 4n. RP-HPLC trace, eluent A 0.10% TFA in water, eluent B 0.10% TFA in CH_3CN/water: 4/1 by vol. C18 Xbridge BEH 300 Å 5 µm (4.6 × 250 mm) column, gradient 0-100% B in 30 min (1 mL min^{-1}, detection UV 215 nm).

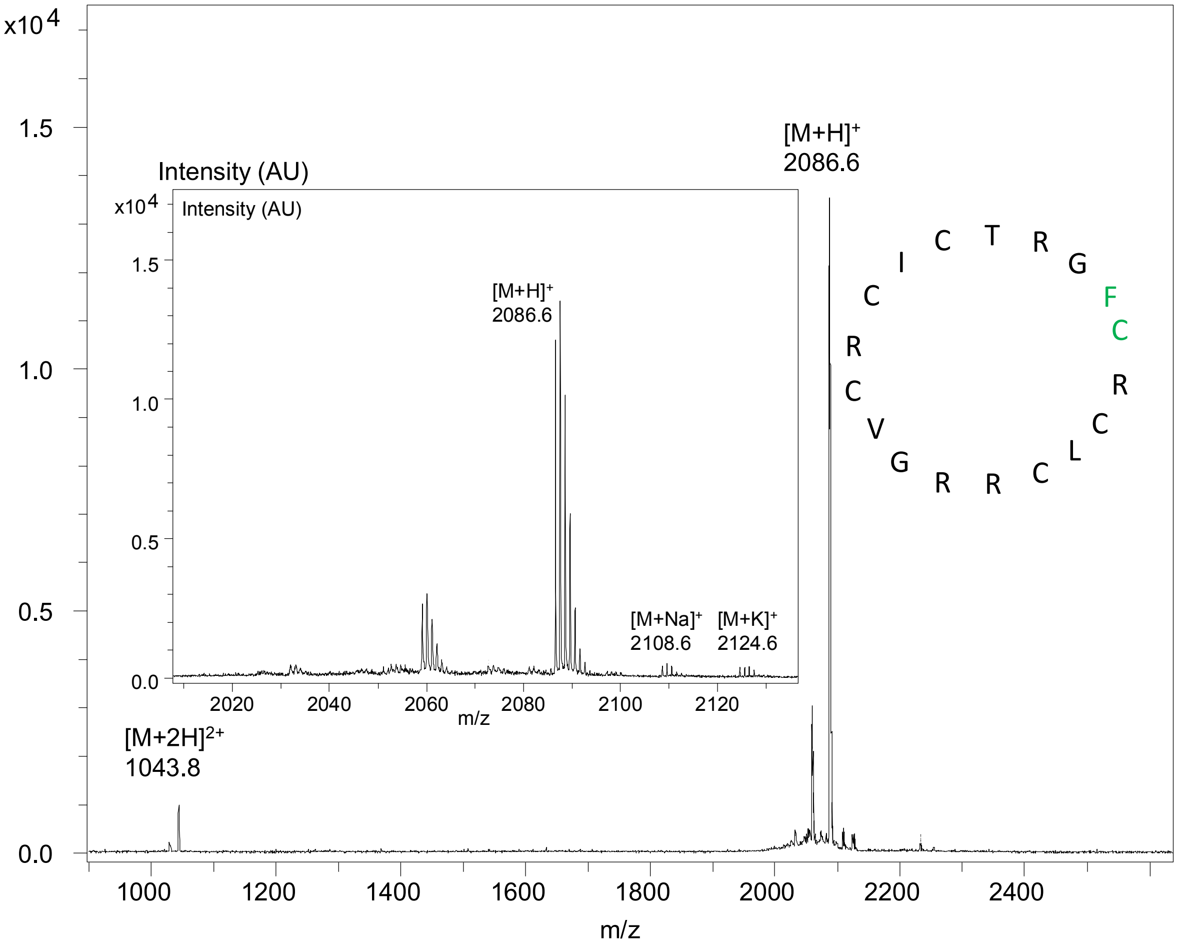

Supplementary Figure 89. MALDI-TOF analysis of RTD-1 peptide produced from 4n. Matrix alpha cyano 4-hydroxycinnaminic acid, positive detection mode, reflector mode, $[M+H]^+$ calcd. (monoisotopic) 2087.0, found 2086.6, $[M+2H]^{2+}$ calcd. (monoisotopic) 1044.0, found 1043.8. See **Supplementary Note**.

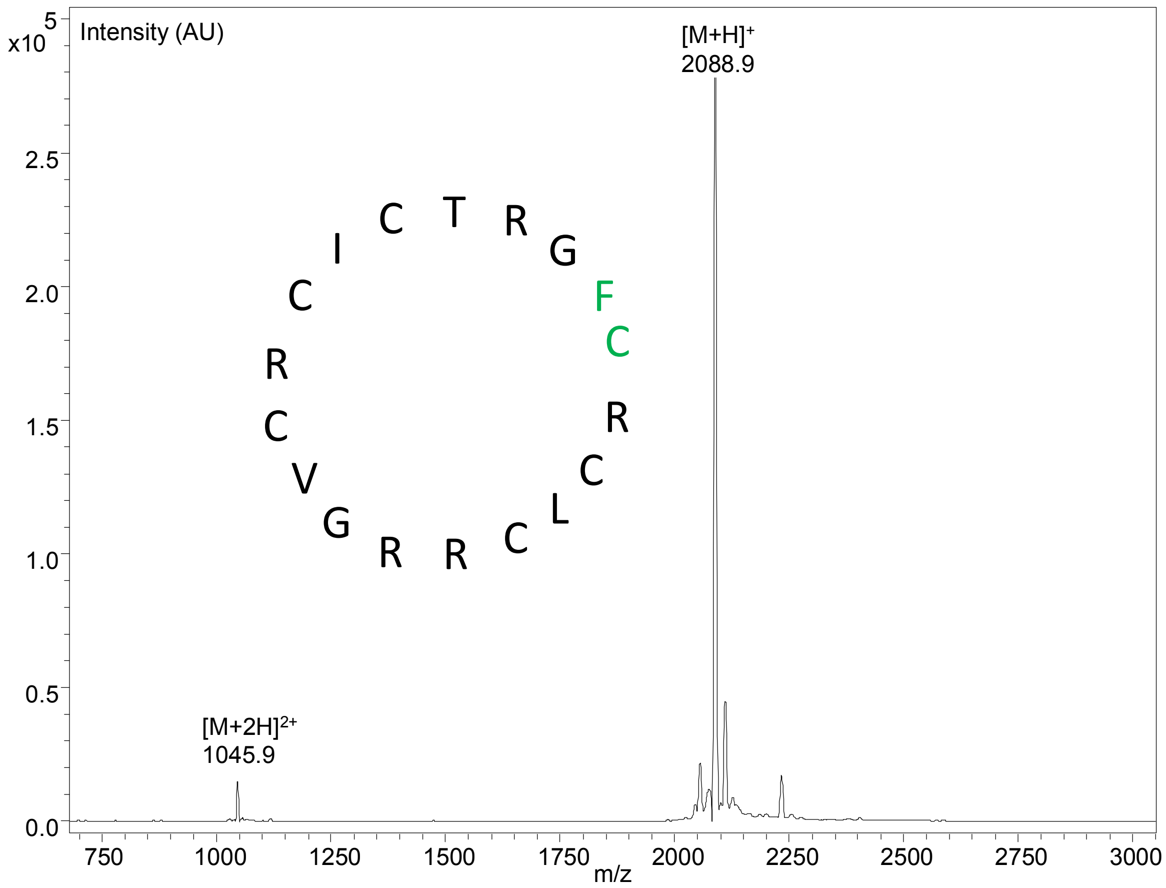

Supplementary Figure 90. MALDI-TOF analysis of RTD-1 peptide produced from 4n. Matrix alpha cyano 4-hydroxycinnaminic acid, positive detection mode, linear mode, $[M+H]^+$ calcd. (mean) 2088.6, found 2088.9. See **Supplementary Note**.

a)

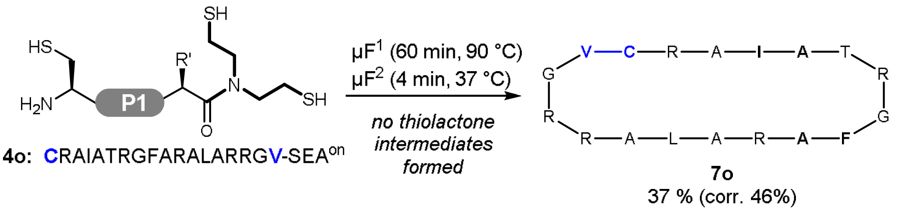

b)

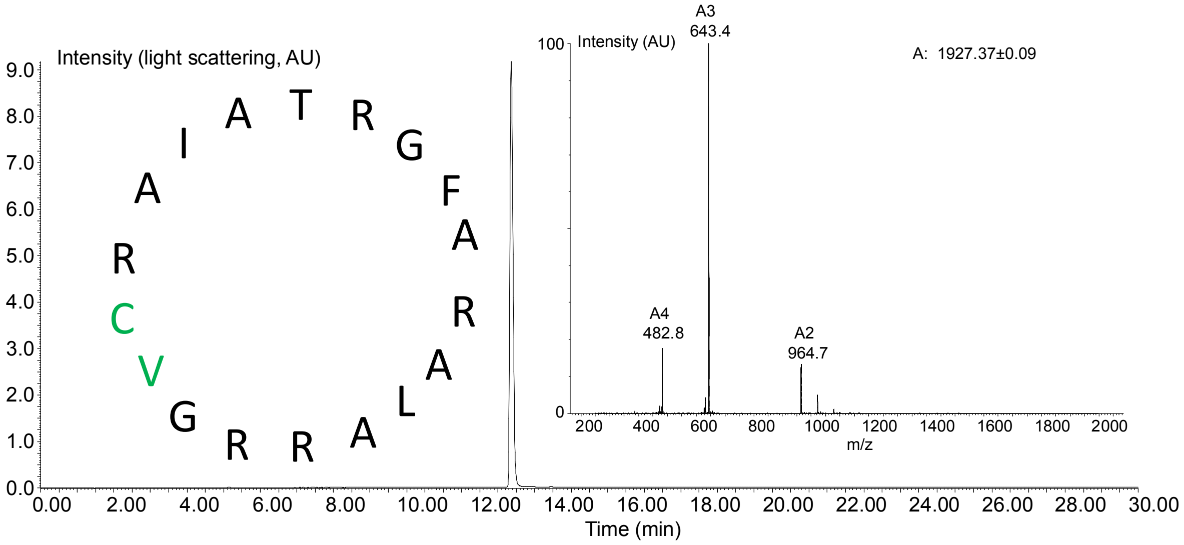

Supplementary Figure 91. a) Synthesis of peptide 7o. b) LC-MS analysis of purified peptide 7o. LC trace, eluent A 0.10% TFA in water, eluent B 0.10% TFA in CH_3CN/water: 4/1 by vol. C18 Xbridge BEH 300 Å 5 µm (4.6 × 250 mm) column, gradient 0-100% B in 30 min (1 mL min^{-1}, detection light scattering). MS trace. $[M+2H]^{2+}$ m/z calcd. (monoisotopic) 964.1, obs 964.7, $[M+3H]^{3+}$ m/z calcd. (monoisotopic) 643.0, obs 643.4, $[M+4H]^{4+}$ m/z calcd. (monoisotopic) 482.5, obs 482.8.

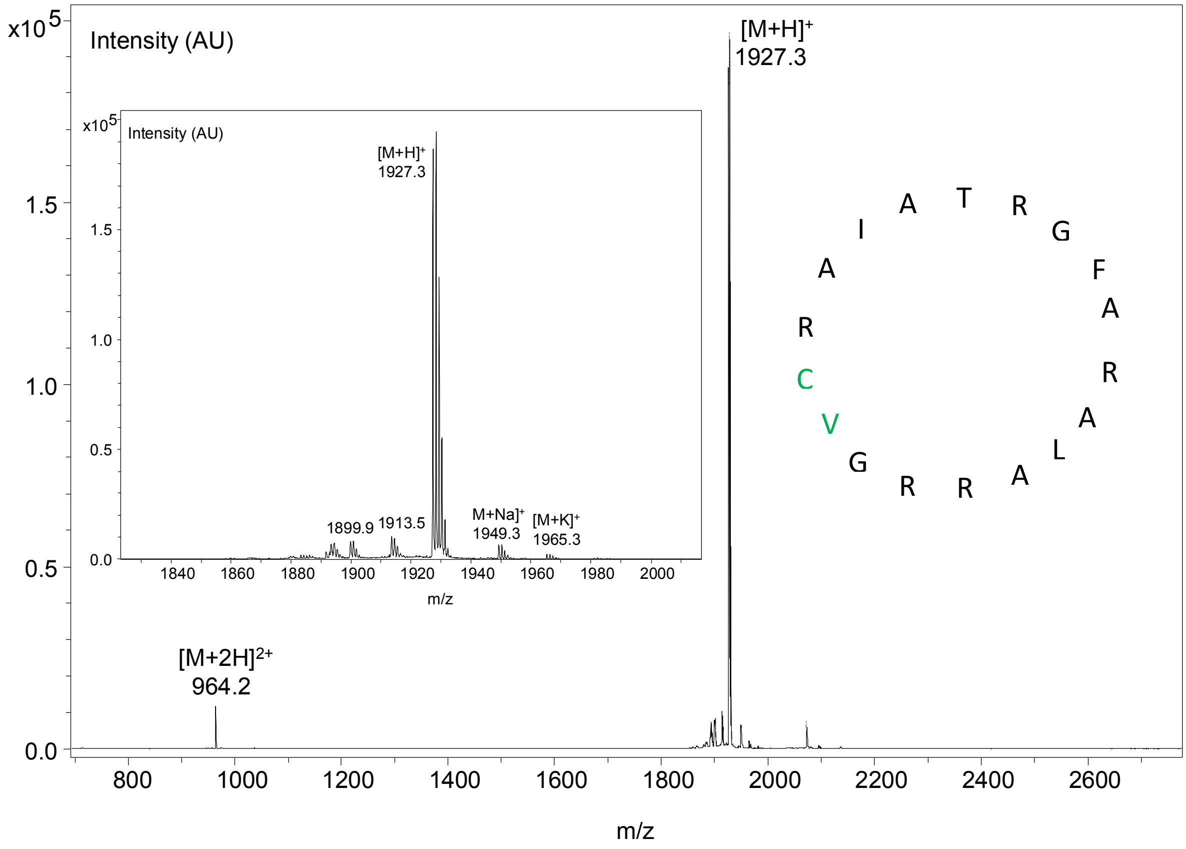

Supplementary Figure 92. MALDI-TOF analysis of peptide 7o. Matrix alpha cyano 4-hydroxycinnaminic acid, positive detection mode, reflector mode $[M+H]^{+}$ calcd. (monoisotopic) 1927.1, found 1927.3, $[M+2H]^{2+}$ calcd. (monoisotopic) 964.1, found 964.2.

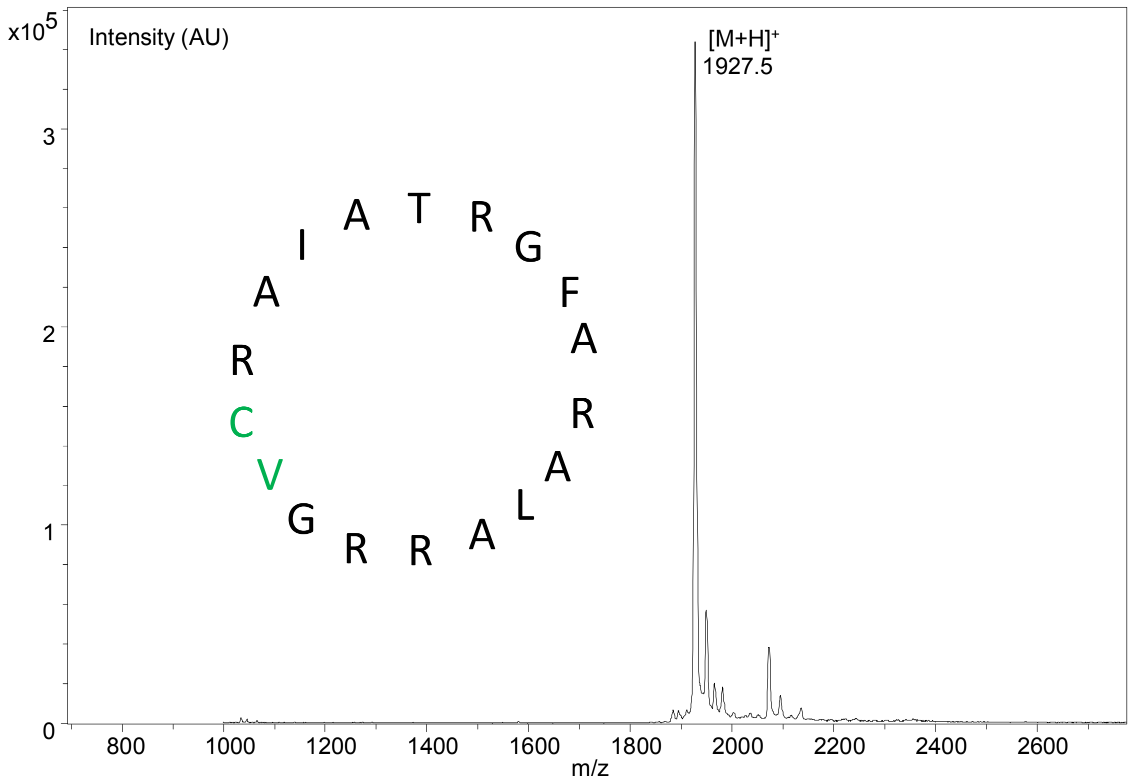

Supplementary Figure 93. MALDI-TOF analysis of peptide 7o. Matrix alpha cyano 4-hydroxycinnaminic acid, positive detection mode, linear mode, $[M+H]^+$ calcd. (mean) 1928.3, found 1927.5.

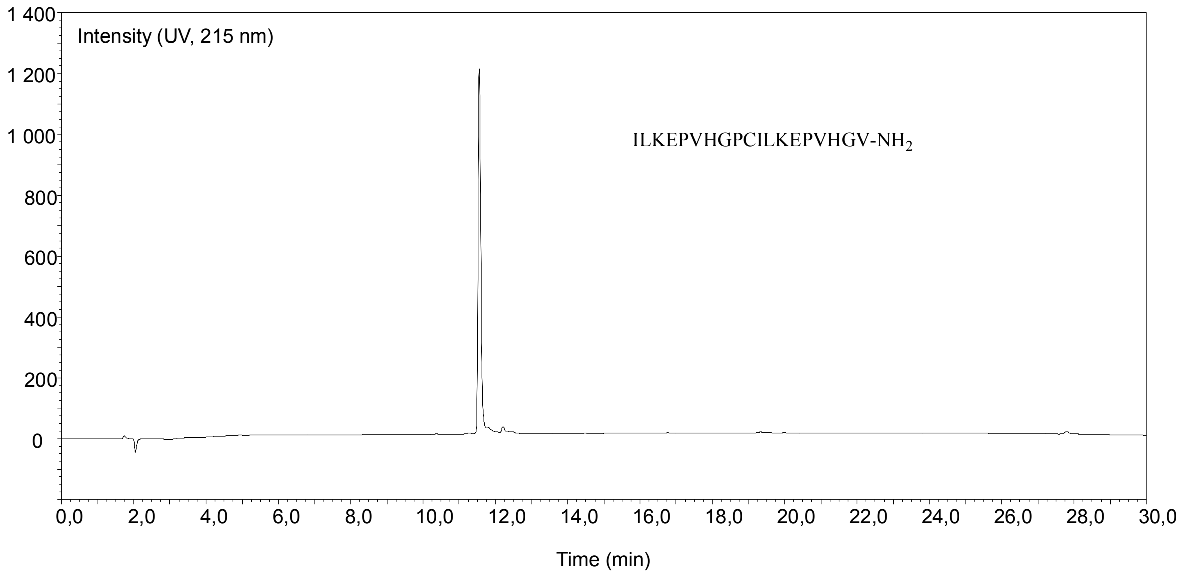

Supplementary Figure 94. RP-HPLC analysis of peptide 11. RP-HPLC trace, eluent A 0.10% TFA in water, eluent B 0.10% TFA in CH_3CN/water: 4/1 by vol. C18 Xbridge BEH 300 Å 5 µm (4.6 × 250 mm) column, gradient 0-100% B in 30 min (1 mL min^{-1}, detection UV 215 nm).

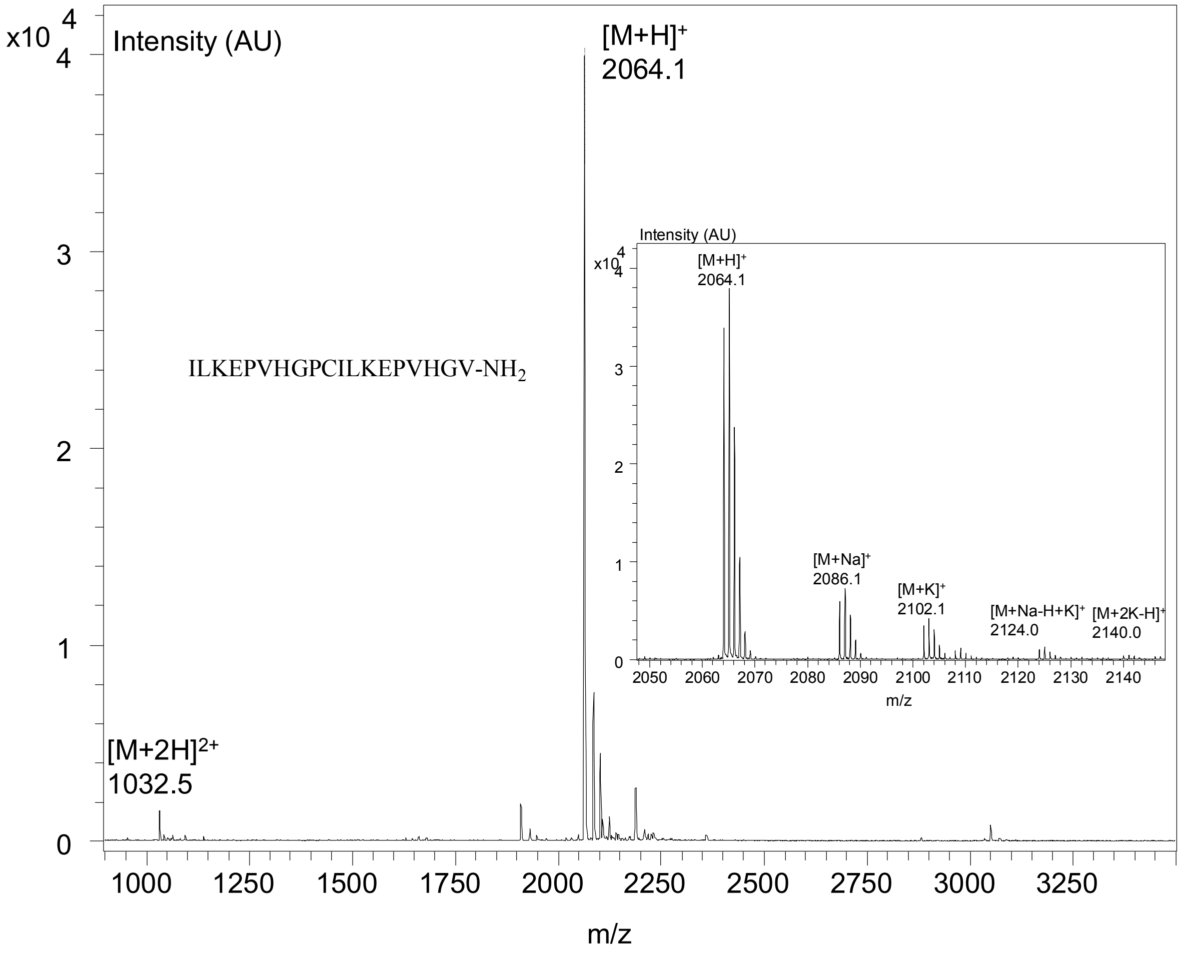

Supplementary Figure 95. MALDI-TOF analysis of peptide 11. Matrix alpha cyano 4-hydroxycinnaminic acid, positive detection mode, $[M+H]^+$ calcd. (monoisotopic) 2064.2, found 2064.1.

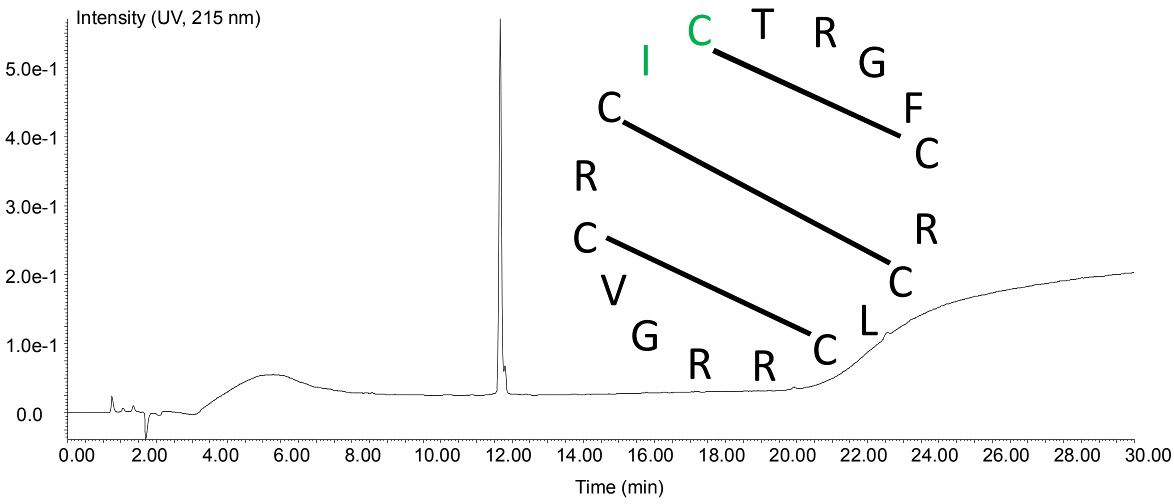

Supplementary Figure 96. RP-HPLC analysis of folded RTD-1 peptide. RP-HPLC trace, eluent A 0.10% TFA in water, eluent B 0.10% TFA in CH_3CN/water: 4/1 by vol. C18 Xbridge BEH 300 Å 5 µm (4.6 × 250 mm) column, gradient 0-100% B in 30 min (1 mL min^{-1}, detection UV 215 nm).

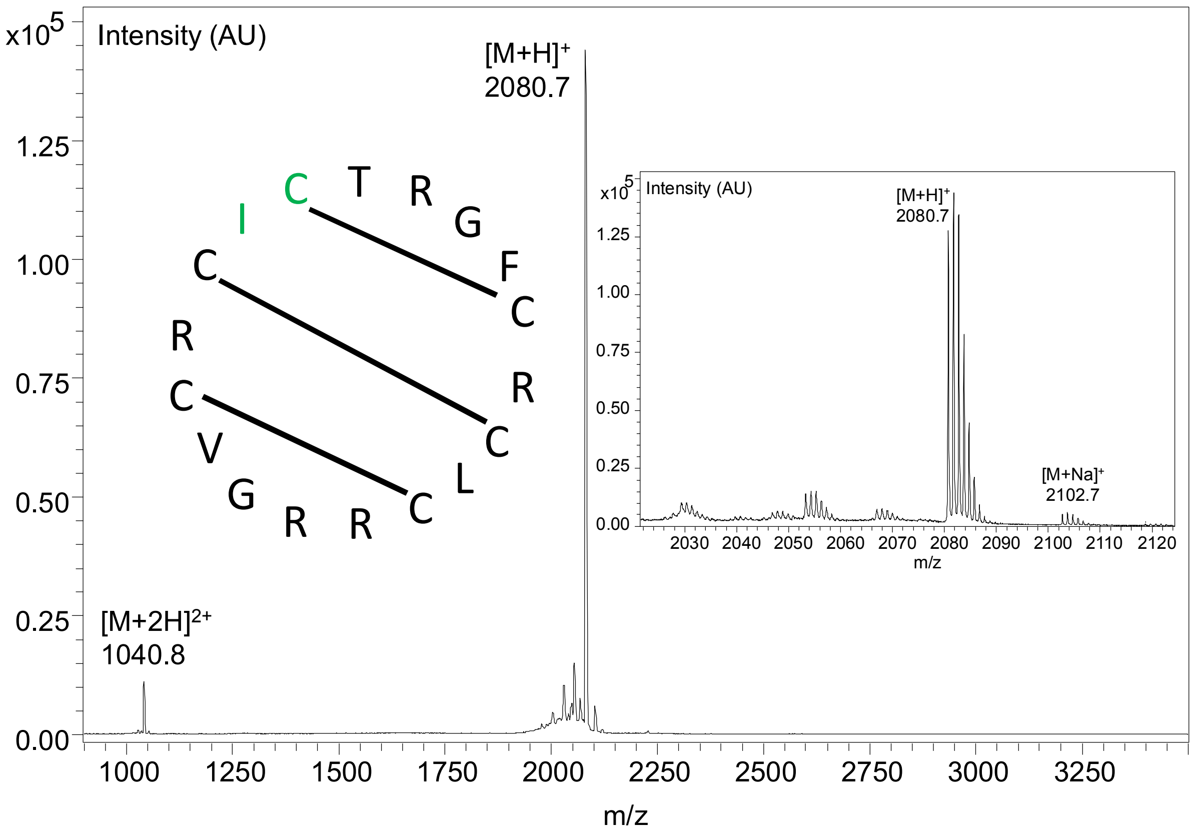

Supplementary Figure 97. MALDI-TOF analysis of folded RTD-1 peptide. Matrix alpha cyano 4-hydroxycinnaminic acid, positive detection mode, reflector mode, $[M+H]^+$ calcd. (monoisotopic) 2080.9, found 2080.7.

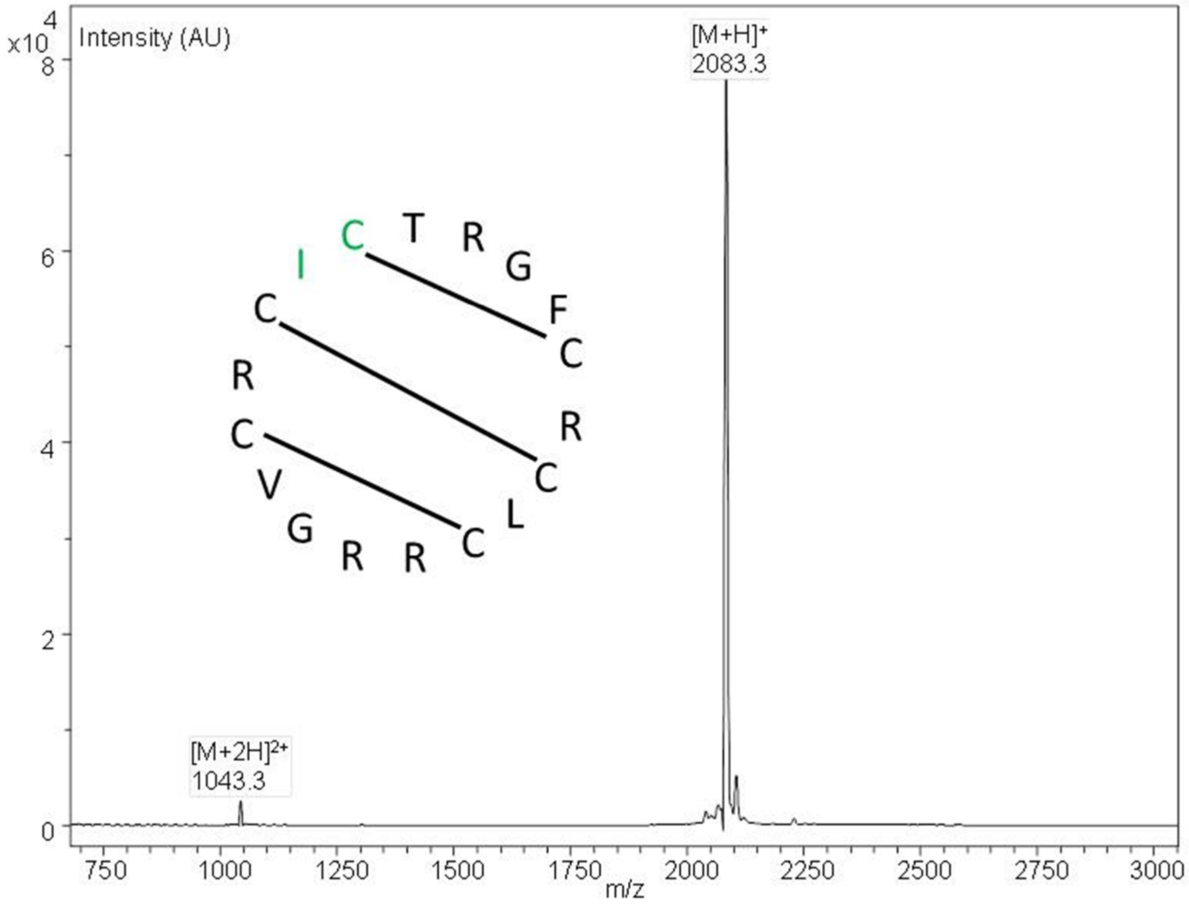

Supplementary Figure 98. MALDI-TOF analysis of folded RTD-1 peptide. Matrix alpha cyano 4-hydroxycinnaminic acid, positive detection mode, linear mode, $[M+H]^+$ calcd. (mean) 2082.6, found 2083.3.

Supplementary Tables

Supplementary Table 1. Connectors, ferules and unions

Item	Details	www.idex-hs.com	Reference
Connectors	One-Piece Fingertight, PEEK, 10-32 Coned, for 1/16” OD	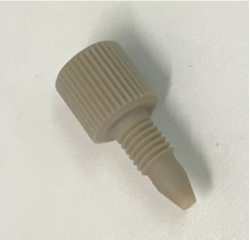	F-120X
	Super Flangeless Nuts, natural PEEK 1/4-28 thread for 1/16” OD tubing	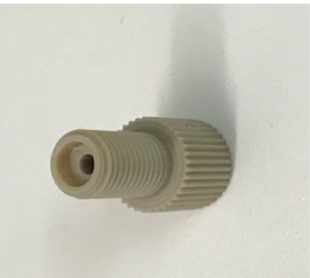	P-255X
Ferrules	Super Flangeless Ferrule Tefzel (ETFE) and SS ring 1/4-28 thread for 1/16” OD tubing	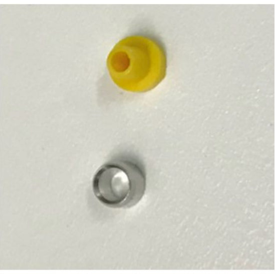	P-259X
Unions	Red ETFE female luer to female FB 1/4-28 quick connect adapter	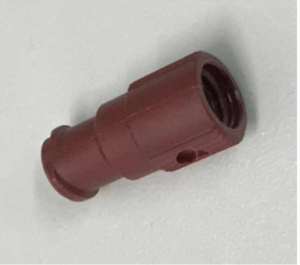	P-678
	Natural polypropylene standard low pressure union 1/4-28	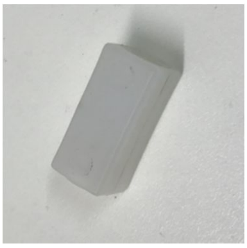	P-620

Supplementary Table 2. Static mixers

Item	Details	www.idex-hs.com	Reference
Mixers	Y-mixer, natural PEEK 1/4-28 thread for 1/16" o.d. tubing, 0.02" through hole	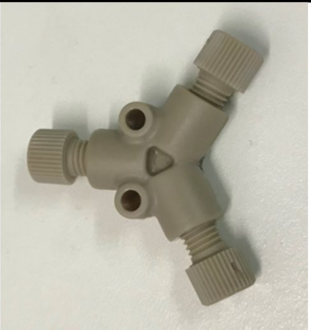	P-512
	Static mixer, natural PEEK 1/4-28 thread for 1/16" o.d. tubing, 0.02" through hole; equipped with a UHMWPE frit (10 µm, 2.2 µL internal volume)	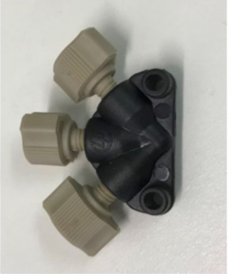	U-466

Supplementary Table 3. Synthesis of SEA peptides 3 or 4.

peptide	scale (mmol)	crude yield	HPLC purified (yield)
**3a** ILKEPVHGV-SEA^{off}	0.25	220 mg (61%)	180 mg (50%)
**3b** TASV-SEA^{off}	0.25	88.4 mg (58%)	57.1 mg (37.6%)
**4c** CILKEPVHGV-SEA^{on}	0.25	215 mg (55%)	-
**4d** CILKEGVHGV-SEA^{on}	0.25	218 mg (57%)	-
**4e** CILNGPVHGV-SEA^{on}	0.2	173 mg (64%)	-
**4f** CILDGPVHGV-SEA^{on}	0.2	143.5 mg (53%)	-
**4g** CILNGPVHGL-SEA^{on}	0.1	83 mg (61%)	-
**4h** CILDGPVHGL-SEA^{on}	0.1	82.2 mg (60%)	-
**4i** CILKEGVHGT-SEA^{on}	0.25	235 mg (62%)	-
**4j** CILKEGVHGI-SEA^{on}	0.25	221 mg (58%)	-
**4k** CILKEGVHGP-SEA^{on}	0.25	228 mg (60%)	-
**4l** CRCICTRGFCRCLCRRGV-SEA^{on}	0.1	150 mg (52%)	68.7 mg (23.6%)
**4m** CTRGFCRCLCRRGVCRCI-SEA^{on}	0.1	166 mg (57%)	86.3 mg (29.7%)
**4n** CRCLCRRGVCRCICTRGF-SEA^{on}	0.1	194 mg (66%)	104 mg (36%)
**4o** CRAIATRGFARALARRGV-SEA^{on}	0.1	-	77.5 mg (28.2%)
**4p** CQPWSSMIPHEHSFLPSSYRGKDLQENY-SEA^{on}	produced by reduction of the SEA^{off} peptide **3p**	36.7 mg	21.3 mg (36%)
**4q** ILKEPVHGP-SEA$^{\mathbf{on}}$	0.1	99 mg (68%)	-

Supplementary Table 4. Conversion of peptide 3b into SEAE peptide 5b in the microfluidic system. Effect of TCEP concentration.

TCEP.HCl (mM)	SEAE peptide **5b** (%)	SEA^{on} peptide **4b** (%)	ratio **5b**/**4b**
50	80.4	15.8	5.1
100	87.2	8.1	10.7

Supplementary Table 5. Results of the kinetic study with peptide 4c.

tubing length (m)	residence time (min)	cyclic peptide (%)[a]	dimeric species (%)[a]
6.25	30	63	21
4.16	20	64	20
2.15	10.32	65	27
1.66	8	67	18
1.25	6	66	14
0.83	4	63	16
0.42	2	68	15
0.21	1	64[b]	23

[a] Conversion determined by RP-HPLC

[b] Peak of the cyclic product is a mixture of the desired cyclic peptide and the MPAA thioester intermediate

Supplementary Table 6. Results for the activation of 4c in microfluidic system and comparison with batch experiment.

Entry	peptide precursor (14 mM feed solution)	conditions	SEA thioester HPLC yield (%)	SEA^{on} HPLC yield (%)	ratio
1	**4c** CILKEPVHGV-SEA^{on}	batch	74.2	5.9	12.5
**2**	**4c** CILKEPVHGV-SEA^{on}	microfluidic	74.5	5	14.9

Supplementary Table 7. Isolated yields for SEAE peptide thioesters 5a-c.

Starting SEA peptide	SEAE peptide	scale	isolated yield
ILKEPVHGV-SEA^{off} **3a**	ILKEPVHGV-SEAE **5a**	8.4 µmol	7.9 mg (60%)
TASV-SEA^{on} **4b**	TASV-SEAE **5b**	0.2 mmol	49.3 mg (34%)
C(*St*Bu)ILKEPVHGV-SEA^{off} **3c**	CILKEPVHGV-SEAE **5c**	5.6 µmol	3.7 mg (42%)

Supplementary Table 8. Mass of SEA^{on} peptides 4 engaged for Feed solution 1, temperature T^1 in µF^1 and residence time in µF^2. T^2 was set at 37 °C.

Entry	SEA^{on} peptides	mass (mg)	T^1 (°C)	t^2 (min)
**1**	**4c** CILKEPVHGV-SEA^{on}	10.87	90	4
**2**	**4d** CILKEGVHGV-SEA^{on}	10.63	90	4
**3**	**4e** CILNGPVHGV-SEA^{on}	9.53	90	4
**4**	**4f** CILDGPVHGV-SEA^{on}	9.48	65	4
**5**	**4g** CILNGPVHGL-SEA^{on}	9.58	65	4
**6**	**4h** CILDGPVHGL-SEA^{on}	9.61	65	4
**3**	**4i** CILKEGVHGT-SEA^{on}	10.60	90	4
**4**	**4j** CILKEGVHGI-SEA^{on}	10.71	90	4
**5**	**4k** CILKEGVHGP-SEA^{on}	10.62	90	4
**6**	**4p** CQPWSSMIPHEHSFLPSSYRGKDLQENY-SEA^{on}	21.30	65[a]	15

Supplementary Table 9. Mass of SEA^{on} peptides 4 engaged for Feed solution 1 and details on the microfluidic parameters.

entry	SEA^{on} peptides	Mass (mg)	T^1 (°C)	T^2 (°C)	t^2 (min)
**1**	**4l** CRCICTRGFCRCLCRRGV-SEA^{on}	20.40	90	45	15
**2**	**4m** CTRGFCRCLCRRGVCRCI-SEA^{on}	20.46	90	45	15
**3**	**4n** CRCLCRRGVCRCICTRGF-SEA^{on}	20.40	65	45	15
**4**	**4o** CRAIATRGFARALARRGV-SEA^{on}	19.28	90	37	4

Supplementary Table 10. Analysis of the effluents coming from µF[1] only (production of 5, activation step) or µF[1] and µF[2] (activation and cyclization into 7).

SEA^{on} peptide precursor **4**	HPLC purity of the crude peptide **4** (%, UV 215 nm)	HPLC purity for peptide **5** (µF^1) (%, UV 215 nm)	yield for **5** (µF^1)	cyclic peptide	HPLC purity for **7** (µF^1 & µF^2, light scattering detection)
**4c CILKEPVHGV-SEA^{on}**	80	75	94	**7c** *c*-(CILKEPVHGV)	56
**4d CILKEGVHGV-SEA^{on}**	79	74	94	**7d** *c*-(CILKEGVHGV)	73
**4e CILNGPVHGV-SEA^{on}**	85	58	68	**7e** *c*-(CILNGPVHGV)	63
**4f CILDGPVHGV-SEA^{on}**	83	36	43	**7f** *c*-(CILDGPVHGV)	43
**4g CILNGPVHGL-SEA^{on}**	80	72	90	**7g** *c*-(CILNGPVHGL)	85
**4h CILDGPVHGL-SEA^{on}**	78	72	92	**7h** *c*-(CILDGPVHGL)	80
**4i CILKEGVHGT-SEA^{on}**	64	60	94	**7i** *c*-(CILKEGVHGT)	73
**4j CILKEGVHGI-SEA^{on}**	72	58	81	**7j** *c*-(CILKEGVHGI)	96
**4k CILKEGVHGP-SEA^{on}**	73	48	66	**7k** *c*-(CILKEGVHGP)	70

Supplementary Table 11. Yields for cyclic peptides 7c-k,p synthesized under microfluidic conditions and workup details.

SEA[on] peptide precursor	work up	cyclic peptide	scale (µmol)	isolated yield (%)	corrected yield (%)
**4c** CILKEPVHGV-SEA[on]	**1**	**7c** *c*-(CILKEPVHGV)	3.7	2.2 mg (46%)	59%
**4d** CILKEGVHGV-SEA[on]	**2 & 1**	**7d** *c*-(CILKEGVHGV)	3.82	2.2 mg (46%)	53%
**4e** CILNGPVHGV-SEA[on]	**2 & 1**	**7e** *c*-(CILNGPVHGV)	2.46	2.3 mg (66%)	-
**4f** CILDGPVHGV-SEA[on]	**2 & 1**	**7f** *c*-(CILDGPVHGV)	4.38	1.8 mg (37%)	58%
**4g** CILNGPVHGL-SEA[on]	**2 & 1**	**7g** *c*-(CILNGPVHGL)	4.15	3.7 mg (80%)	100%
**4h** CILDGPVHGL-SEA[on]	**2 & 1**	**7h** *c*-(CILDGPVHGL)	2.78	2 mg (64%)	100%
**4i** CILKEGVHGT-SEA[on]	**2 & 1**	**7i** *c*-(CILKEGVHGT)	3.84	2.4 mg (49%)	-
**4j** CILKEGVHGI-SEA[on]	**2 & 1**	**7j** *c*-(CILKEGVHGI)	4.16	2.7 mg (51%)	54%
**4k** CILKEGVHGP-SEA[on]	**2 & 1**	**7k** *c*-(CILKEGVHGP)	4.17	2 mg (38%)	47%
**4p** CQPWSSMIPHEHSFLPSSYRGKDLQENY-SEA[on]	**1**	**7p** *c*-(CQPWSSMIPHEHSFLPSS YRGKD)	2.40	3.7 mg (41%)	-

Supplementary Table 12. Yields for cyclic peptides 7l-o. All the effluents were treated according to workup 1.

SEA^on peptide precursor	cyclic peptide	scale (µmol)	isolated yield (%)	corrected yield (%)
**4l** CRCICTRGFCRCLCRRGV-SEA^on	RTD-1 (reduced) *c*-(CRCICTRGFCRCLCRRGV)	3.82	3 mg (29%)	33%
**4m** CTRGFCRCLCRRGVCRCI-SEA^on	RTD-1 (reduced) *c*-(CTRGFCRCLCRRGVCRCI)	2.78	2.5 mg (34%)	43%
**4n** CRCLCRRGVCRCICTRGF-SEA^on	RTD-1 (reduced) *c*-(CRCLCRRGVCRCICTRGF)	3.47	2.1 mg (23%)	-
**4o** CRAIATRGFARALARRGV-SEA^on	**7o** *c*-(CRAIATRGFARALARRGV)	3.82	3.5 mg (37%)	46%

Supplementary Table 13. Results of the control experiment (ligation of 4q and 10 under microfluidic conditions).

starting peptides	peptide produced	scale (µmol)	isolated mass (yield)	corrected yield	Comments
ILKEPVHGP-SEA^on **4q** CILKEPVHGV-NH_2 **10**	ILKEPVHGP-CILKEPVHGV-NH_2 **11**	2.77	1.5 mg (21%)	28%	with decane and pressure regulator

Supplementary Table 14. Antibacterial activity of RTD-1 peptide.[a]

	E.coli BW25113		*S.aureus* SH1000	
[RTD-1] (μg mL^{-1})	Log(cfu mL^{-1})	SD	Log(cfu mL^{-1})	**SD**
0.0	6.69	0.07	5.60	0.11
0.25	6.73	0.04	5.55	0.05
0.5	6.71	0.01	5.60	0.04
1.0	6.65	0.12	5.57	0.10
2.0	4.52	0.05	3.39	0.11
4.0	2.81	0.32	2.78	0.25
8.0	<2		<2	
16.0	<2		<2	

[a] The limit of detection is 100 cfu mL^{-1}. The experiment was done in triplicate. The data correspond to the mean and standard deviation of the log10 transformed colony-forming units (cfu) per mL.

Supplementary Methods

Reagents and solvents

2-(1*H*-Benzotriazol-1-yl)-1,1,3,3-tetramethyluronium fluorophosphate (HBTU) and *N*-Fmoc protected amino acids were obtained from Iris Biotech GmbH. Side-chain protecting groups used for the amino acids were Fmoc-Arg(Pbf)-OH, Fmoc-Asn(Trt)-OH, Fmoc-Asp(O*t*Bu)-OH, Fmoc-Gln(Trt)-OH, Fmoc-Glu(O*t*Bu)-OH, Fmoc-His(Trt)-OH, Fmoc-Lys(Boc)-OH, Fmoc-Ser(*t*Bu)-OH, Fmoc-Thr(*t*Bu)-OH, Fmoc-Trp(Boc)-OH, Fmoc-Tyr(*t*Bu)-OH, Fmoc-Cys(S*t*Bu)-OH or Fmoc-Cys(Trt)-OH. Synthesis of *bis*(2-sulfanylethyl)aminotrityl polystyrene (SEA PS) resin was carried out as described elsewhere.[1] 4-Mercaptophenylacetic acid (97%, MPAA), 3-mercaptopropionic acid (MPA), *tris*(2-carboxyethyl)phosphine hydrochloride (≥98%), TCEP), triisopropylsilane (TIS), dimethyl sulfide (DMS), guanidine hydrochloride (≥99%), sodium phosphate dibasic dihydrate (≥99%), hydrochloric acid (reagent grade, 37%) and sodium hydroxide (pellets, 97%) were purchased from Sigma-Aldrich. All other reagents were purchased from Acros Organics or Merck and were of the purest grade available.

Peptide synthesis grade *N,N*-dimethylformamide (DMF), dichloromethane (CH_2Cl_2), diethylether (Et_2O), acetonitrile (CH_3CN), heptane, LC–MS-grade acetonitrile (CH_3CN, 0.1% TFA), LC–MS-grade water (H_2O, 0.1% TFA), *N,N*-diisopropylethylamine (DIEA), acetic anhydride (Ac_2O) were purchased from Biosolve and Fisher-Chemical. Trifluoroacetic acid (TFA) was obtained from Biosolve. Decane (synthesis grade) was purchased from Merck. Water was purified with a Milli-Q Ultra Pure Water Purification System.

Standardization of NaOH and HCl solutions

Standardized NaOH solution was prepared as follows. Approximately 1 L of deionized water was boiled for at least 15 min. Upon heating, conditioning of a 500 mL polyvinyl chloride (PVC) bottle was done twice before filling the PVC with the decarbonated water. The remaining air was removed and the bottle was sealed tightly. Afterwards, the bottle was cooled down in an ice bath. To prepare 500 mL of a NaOH solution ~4.5 M, approximately 90 g of sodium hydroxide were weighted and rapidly transferred in a 500 mL PVC bottle placed in an ice bath. Decarbonated water was slowly poured inside the PVC bottle containing the sodium hydroxide pellets while manually stirring the solution. When the bottle was filled and the pellets dissolved, the remaining air was removed and the bottle was sealed tightly. Afterwards, the bottle was cooled down in an ice bath. The exact titration of the NaOH solution was carried out using oxalic acid dihydrate. Each week a new NaOH solution was prepared and titrated in triplicate. Titration was carried out with an automated Metrohm Titrino 799 GPT.

Standardized HCl solution was prepared as follows. A stock solution of HCl (~6 M) was prepared, and then titrated with the standard aqueous solution of NaOH using phenolphthalein as an indicator. The HCl stock solution was titrated every week in triplicate. Titration was carried out with an automated Metrohm Titrino 799 GPT.

Analyses and purifications

Products were characterized by analytical LC–MS (Waters 2695 LC/ZQ 2000 quadripole) on a reverse phase column XBridge BEH300 C18 (3.5 μm, 300 Å, 4.6 × 150 mm) at 30 °C using a linear gradient: 0-100% of eluent B in eluent A over 30 min at a flow rate of 1 mL min^{-1} (eluent A = 0.1% TFA in H_2O; eluent B = 0.1% TFA in CH_3CN/H_2O: 4/1 by vol). The column eluate was monitored by UV at 215 nm and by evaporative light scattering (ELS, waters 2424). The peptide masses were measured by on-line LC–MS: Ionization mode ES+, m/z range 350–2040, capillary voltage 3 kV, cone voltage 30 V, extractor voltage 3 V, RF lens 0.2 V, source temperature 120 °C, desolvation temperature 350 °C. Calculated masses were based on average isotope composition.

MALDI-TOF mass spectra were recorded with a Bruker Autoflex Speed using alpha cyano 4-hydroxycinnaminic acid, sinapinic acid or 2,5-dihydroxybenzoic acid (DHB) as matrix. The observed m/z corresponded to the monoisotopic ions, unless otherwise stated.

Preparative reversed-phase HPLC of crude peptides were performed with an preparative HPLC Waters system using a reverse phase column XBridge BEH300 Prep C18 (5 μm, 300 Å, 10 × 250 mm) and an appropriate linear gradient of increasing concentration of eluent B in eluent A (flow rate of 6 mL min^{-1}). Selected fractions were then combined and lyophilized.

Microfluidic reactor elements

Chemyx Nexus 6000 syringe pumps were used to handle the feed solutions of peptides, *tris*(2-carboxyethyl) phosphine hydrochloride (TCEP.HCl), 4-mercaptophenylacetic acid (MPAA), guanidine hydrochloride (Gn.HCl) and decane. The feed solutions were loaded into Hamilton Gastight Luer Lock Glass syringes (1 and 5 mL).

The microfluidic assemblies were constructed from commercially available metal-free, biocompatible parts.

Microfluidic reactor coils were constructed from high purity perfluoroalkoxy alkane (PFA) capillary (1.58 mm outer diameter, 500 µm internal diameter, purchased from IDEX/Upchurch) of various internal volumes. Connections between the microfluidic reactor loops were designed to ensure minimal dead volumes. PFA microreactor loops were maintained in thermoregulated oil baths (Heidolph™ MR Hei-Tec® equipped with Pt-1000 temperature sensors).

Connectors, ferrules and unions were purchased from IDEX/Upchurch and are detailed in **Supplementary Table 1**.

PEEK Y-mixer and PEEK arrow head-mixer were purchased from IDEX/Upchurch and are detailed in **Supplementary Table 2**.

Metal-free biocompatible in-line check valves were purchased from IDEX/Upchurch (PEEK/perfluoroelastomer 1/4-28 thread for 1/16" o.d. tubing, 34 µL swept volume; reference: CV-3330) and were inserted on the outlet of each syringe.

A dome-type back-pressure regulator (BPR) was used (Zaiput Flow Technologies). All wetted parts are made of perfluorinated polymer. The BPR was connected to a compressed gas cylinder (argon) to set the working pressure (2.5 bar).

In-line reaction monitoring was carried out with a FlowIR™ (SN# 2964) from Mettler-Toledo equipped with a DTGS detector using HappGenzel apodization, a Silicon probe connected via a FlowIR™ sensor and a high pressure heated 10 µL cell. Sampling was performed from 4000 to 650 cm^{-1} at 8 wavenumber resolution with 128 scans.

Segmented regime and back-pressure

During the preliminary trials for the implementation of *N,S*-acyl shift toward SEAE peptide thioesters under microfluidic conditions, two important observations were made: (a) for a model reaction, higher conversions were obtained in batch (91%) than under microfluidic conditions (80%) and (b) gas segments were observed in the microfluidic setup. The first observation suggested that the *N,S*-acyl shift was sensitive to dispersion effects (residence time distribution). The unexpected appearance of gas in the microfluidic setup also affected the control over the residence time.

Dispersion effects were suppressed by the upstream concomitant injection of an immiscible carrier (decane) to force the system back to an ideal plug flow regime (**Supplementary Figure 12**). With the immiscible carrier, the conversion for the *N,S*-acyl shift reached 90%. The use of immiscible carriers is a standard procedure for the suppression of dispersion effects.[2,3]

During preliminary experiments we also observed the formation of a gaseous HCl (see **Supplementary Figure 21**). Its deleterious effect on the control of the residence time was suppressed by inserting downstream a back pressure regulator set at 2.5 bar (see **Supplementary Figure 16** and **Supplementary Figure 17**).

Starting, collecting and shutdown procedures for the microfluidic systems

To start the microfluidic system, the temperature was set to the desired process temperature (T^1 for μF^1, T^2 for μF^2) and the system was equilibrated for 1.5 residence times by injecting Feeds at the appropriate flow rates. The reactor effluent collected during the stabilization phase was discarded.

At steady state, the reactor effluent was treated according to workup procedure 1 or workup procedure 2 and 1 (see *Optimized procedure under microfluidic conditions for peptide cyclization* in this section).

To stop the microfluidic system, the reactor was thoroughly flushed with deionized water (all Feeds 0.1 mL min^{-1}) for 15 min.

Synthesis of the different peptides

SEA peptides were produced by Fmoc-SPPS as described elsewhere.[4] The sequences and yields of synthesis are indicated in **Supplementary Table 3.** The characterization of peptides **3a** and **4a** can be found elsewhere.[1,5]

Peptide CILKEPVHGV-MPA **1c** was synthesized as follows. TCEP.HCl (114.9 mg, 0.4008 mmol) was dissolved in water (4.5 mL). 3-mercaptopropionic acid (500 µL, 10% by volume) was added and the pH was adjusted to 3.0 by adding 6 M NaOH (115 µL).

Peptide **4c** (51.7 mg, 33.2 µmol) was dissolved in the above solution (4.75 mL) and placed at 55°C overnight. Then, glacial acetic acid was added to the crude mixture (475 µL) which was extracted three times with diethyl ether. The crude product was purified by HPLC (C18XBridge column, 0 to 15% eluent B in 5 min, then 15 to 45% eluent B in 90 min, eluent A = water containing 0.1% TFA, eluent B = acetonitrile in water 4/1 containing 0.1% TFA, 50 °C, detection at 215 nm, 6 mL min^{-}1) to give 16.5 mg of peptide **1c** (32%).

MPAA peptide thioester **2c** was prepared from SEA^{on} peptide **4c** as described in **Supplementary Figure 27**. This peptide was contaminated by ~15% of starting peptide **4c** that could not be separated during the HPLC purification.

Solution 1: TCEP.HCl (86.4 mg, 0.301 mmol, 0.1 M) was dissolved in water (3 mL). 6 M HCl (38 µL) was added to adjust the pH to 1.03.

Solution 2: The peptide (**4c**, 25.5 mg, 16 µmol) was dissolved in solution 1 (2.342 mL) and placed at 90 °C for 1 hour.

Solution 3: Gn.HCl (14.33 g, 150.0 mmol, 6 M) was dissolved in water (15 mL, 25 mL final volume). $Na_2HPO_4.2H_2O$ (890.9 mg, 5.005 mmol, 0.2 M) and MPAA (1.68 g, 9.98 mmol, 0.4 M) was dissolved in aqueous Gn.HCl solution. 6 M NaOH (1.570 mL) was added to adjust the pH to 5.96.

Solution 2 (2.342 mL) was added to solution 3 (24.82 mL), and the resulting solution was thoroughly mixed. After 4 min, glacial acetic acid (2.716 mL) was added to stop the reaction. After 10 extractions with diethyl ether, the solution was purified by RP-HPLC to give 3.3 mg (13%) of peptide CILKEPVHGV-MPAA. Conditions : C18XBridge column, 0 to 30% eluent B in 5 min, then 30 to 50% eluent B in 60 min, eluent A = water containing 0.1% TFA, eluent B = acetonitrile in water 4/1 containing 0.1% TFA, 50 °C, detection at 215 nm, 6 mL min^{-1}.

Peptide **2c** was found to partially decompose upon storage at -20 °C in the lyophilized form over a period of 6 months.

Each peptide was analyzed to demonstrate its purity and identity (see from **Supplementary Figure 30** to **Supplementary Figure 68**).

Evidence for the exceptional reactivity of SEAE peptide thioesters in batch

The reaction of SEAE peptide **5a** or MPA peptide thioester **1a** with MPAA in batch was performed as follows.

MPA peptide thioester ILKEPVHGV-$SCH_2CH_2CO_2H$ **1a** was produced as described elsewhere.[5] SEAE peptide thioester **5a** was produced as described hereinafter in *Optimized procedure under microfluidic conditions for SEAE peptide **5** synthesis* (see also **Supplementary Table 7**).

Step 1: TCEP.HCl (6.01 mg, 21.0 µmol, 0.1 M concentration) was dissolved in water (209.7 µL). 6 M HCl (2 µL) was added to adjust the pH to 1.07. The peptide thioester **5a** (0.986 mg, 0.629 µmol) or **1a (**1.04 mg, 0.732 µmol) was then dissolved in this solution (50 µL, 14 mM concentration).

Step 2: $Na_2HPO_4.2H_2O$ (106.4 mg, 0.5978 mmol, 0.4 M final concentration) was dissolved in water (1.167 mL). 6 M NaOH (333.33 µL, 2.000 mmol) was added. The solution was mixed and added to solid MPAA (201.9 mg, 1.200 mmol, 0.8 M concentration).

Step 3: The solution prepared at Step 2 (50 µL) was used to dilute the peptide solution containing **5a** or **1a** (50 µL) prepared at Step 1. 6 M NaOH (4 µL) was added quickly to adjust the pH to 7.0. Final concentrations are: peptide 7 mM, TCEP 50 mM, MPAA 0.4 M, Na_2HPO_4 0.2 M

Aliquots (2 µL) were analyzed after quenching the reaction with (10% aqueous acetic acid, 100 µL water). MPAA was removed by extracting the aqueous phase with diethylether (5 times). Samples were analyzed by LC-MS using a C18XBridge column.

The results of this experiment are presented in Fig. 2c in the manuscript.

Evidence for the exceptional reactivity of SEAE peptide thioesters under microfluidic conditions

In this experiment, the optimal microfluidic setup described in **Supplementary Figure 17** was adapted to determine the rate of the conversion of the SEAE peptide thioester **5a** into MPAA peptide thioester **2a**. The adapted microfluidic system is described in **Supplementary Figure 13**. To avoid the cyclization process during this experiment, the study was performed with SEA peptide **4a** ILKEPVHGV-SEA^{on}. SEA peptide **4a** was rearranged in batch at 90 °C for 1 h (7 mM, pH 1) After cooling, this solution was used to feed the microfluidic system, thereby overcoming the need for the activation module μF^1 (Feed solution 1 in **Supplementary Figure 13**).

The residence time in the second microfluidic module was varied by adapting the length of the capillary (1/16” o.d., 500 µm i.d.) accordingly. Residence times below 15 s could not be studied due to the configuration of the microfluidic system. Feed solution 1 (7 mM SEAE peptide **5a** at pH 1, ϕ_1 = 3.3 µL min^{-1}) was mixed through a static Y mixer with decane (Feed solution 2, ϕ_2 = 3.3 µL min^{-1}) to induce segmented regime at room temperature to mimic the full operational system, which was next

mixed with Feed solution 3 (MPAA at pH 7.8, ϕ_3 = 35 µL min^{-1}). The reaction mixture was next reacted in ligation module µF_2 at 37 °C at various residence times. The reactor effluent was collected, processed and analyzed.

The experiment described here shows that SEAE peptide thioester **5a** was converted into MPAA thioester **2a** in less than 15. The yield for MPAA peptide thioester **2a** is almost constant during the first 2 min, and then slightly decreases over time most likely as a consequence of the displacement of MPAA thioester **2a** by SEA amine **6** within the microfluidic reactor (see Figure 2c in the article for a similar effect under batch conditions).

The results for this experiment are presented in **Supplementary Figure 19** and in Fig. 2d in the manuscript.

*Reaction of MPA peptide thioester **1a** with MPAA under microfluidic conditions*

In this experiment, we used the system and conditions described in **Supplementary Figure 13** and in the previous section to determine the kinetic of the reaction of MPA peptide **1a** with MPAA under microfluidic conditions.

MPA peptide **1a** was dissolved at 7 mM at pH 1 and used for feeding the microfluidic system.

The results for this experiment are presented in **Supplementary Figure 20** and in Fig. 2d in the manuscript. Only 2.3% of the MPAA peptide thioester **2a** was produced after 15 s of residence time.

*NCL with SEAE peptide thioester **5a**. Comparison with MPA thioester **1a** (batch)*

Step 1: TCEP.HCl (3.17 mg, 11.1 µmol, 0.1 M) was dissolved in water (110.4 µL). 6 M HCl (1.3 µL) was added to adjust the pH to 1.06. The peptide thioester **5a (**1.199 mg, 0.7654 µmol) or **1a (**0.997 mg, 0.701 µmol, 14 mM) was then dissolved in this solution (50 µL).

Step 2: $Na_2HPO_4.2H_2O$ (106.2 mg, 0.5967 mmol, 0.4 M) was dissolved in water (1.167 mL). 6 M NaOH (333.33 µL, 2.0000 mmol) was added. This solution was mixed and added on solid MPAA (201.86 mg, 1.2000 mmol, 0.8 M).

Step 3: The cysteinyl peptide CILKEPVHGV-NH_2 **10** (1.504 mg, 1.048 µmol, 1.5 eq, 21 mM) was dissolved in the solution prepared at Step 2 (50 µL) and mixed with the peptide solution containing the peptide thioester **5a** or **1a** (50 µL) and prepared at Step 1. 6 M NaOH (4 µL) was added quickly to adjust the pH to 7.0. Final concentrations are: peptide thioester **1a** or **5a** 7 mM, Cys peptide 10.5 mM, TCEP 50 mM, MPAA 0.4 M, Na_2HPO_4 0.2 M.

Aliquots (2 µL) were analyzed after quenching the reaction with 10% aqueous acetic acid (100 µL). MPAA was removed by extracting the aqueous phase with diethylether (5 times). Samples were analyzed by LC-MS using a C18XBridge column.

The data are presented in **Supplementary Figure 1**.

*Effect of the temperature on the rate of SEAE peptide thioester **5** formation*

The effect of temperature on the formation rate of SEAE peptide thioesters **5** was performed in batch using SEA^{off} peptide **3b**. The data are presented in **Supplementary Figure 2**.

Solution 1: TCEP.HCl (5.91 mg, 20.6 µmol, 0.2 M) was dissolved in water (103 µL).

Solution 2: Peptide **3b** (423 µg, 0.696 µmol, 21 mM) was dissolved in water (33.2 µL).

Solution 1 (66 µL) and solution 2 (33 µL) were mixed together and 5 M NaOH (5 µL) was added to adjust the pH to 3.9 in order to trigger the reduction of the SEA^{off} group into SEA^{on}. The reduction was monitored by MALDI-TOF. Once the reduction was complete, the pH was adjusted to 1.05 by adding 5 M HCl (5.5 µL). The reaction mixture was heated under nitrogen atmosphere and the conversion of SEA^{on} peptide **4b** into SEAE peptide thioester **5b** was determined by RP-HPLC.

Identification of the gaseous product formed in the first microfluidic reactor µF^1

A 100 mM TCEP.HCl aqueous solution at pH 1 was injected at a flow rate of 3.3 µL min^{-1} in a microfluidic setup constructed from 1 m of PFA capillary (1.58 mm O.D., 500 µm I.D.; 0.2 mL internal volume, described in **Supplementary Figure 14**). The setup included an in-line IR spectrometer that enabled real-time reaction monitoring. A loaded dome-type back-pressure regulator was inserted downstream, and dynamically controlled (0 – 2.5 bar).

At an operating temperature of 25 °C with no counter-pressure, a homogeneous stream was obtained. At 90 °C with no counter-pressure, irregular gas segments were observed in the microfluidic setup, while with a 2.5 bar back-pressure, a homogeneous stream was recovered. At 90 °C with no counter-pressure, the IR spectrometer indicated the appearance of the typical vibration bands of HCl and HCl hydrate (**Supplementary Figure 21**),[6] while at 25 °C no such signals were detected. Increasing the counter-pressure to 2.5 bar suppressed the formation of gaseous HCl. No other gaseous products such as CO_2 or ethylene that could be expected from thermal degradation of TCEP were detected.

*Stability of SEA^{off} peptides of type **3** at pH 1 in the presence of TCEP at room temperature*

In this experiment, we were wondering if the SEA^{off} peptides of type **3** would be reduced by TCEP at pH 1 at room temperature (conditions used for feeding the microfluidic system), since TCEP can reduce disulfides at an appreciable rate only at pH > 3.[7]

Therefore, peptide **3b** (7 mM) was dissolved at pH 1 in the presence of TCEP.HCl (0.2 M). The solution was kept at room temperature or at 90 °C. The formation of the reduced SEA^{on} peptide **4b** was monitored qualitatively by MALDI-TOF mass spectrometry (Supplementary Figure 3). The data show that SEA^{off} peptide **3b** is resistant to reduction by TCEP at pH 1, since SEA^{on} peptide **4b** is a minor component in the mixture after 3 h at room temperature (**Supplementary Figure 3B**). In contrast, reduction proceeds quickly at 90 °C (**Supplementary Figure 3C**).

*Feeding the microfluidic system with SEA^{off} peptides of type **3**. Optimization of TCEP concentration*

The concentration of TCEP.HCl (50 mM or 100 mM) was optimized under microfluidic conditions using SEA^{off} peptide TASV-SEA^{off} **3b** and the microfluidic system described in **Supplementary Figure 16** and **Supplementary Figure 22** (1 m tubing, 500 µm diameter, 3.3 µL min^{-1}, residence time 1 h, 90 °C).

Peptide **3b** was dissolved at 14 mM final concentration in the TCEP.HCl solution (50 mM or 100 mM) at pH 1.1. This solution was used to feed the microfluidic system. The solution coming from the microfluidic system was collected and the conversion of **3b** into **5b** was determined by HPLC. The best result was obtained using 100 mM of TCEP (see **Supplementary Table 4**). Therefore, this concentration was selected for all subsequent studies.

Optimization of the pH in the second microreactor µF^2

The microfluidic system used for this experiment is described in **Supplementary Figure 15**.

Feed solution 1 was prepared using SEA^{on} peptide **4c**. 21.78 mg (14 µmol) of the starting SEA^{on} peptide **4c** and 28.66 mg of TCEP.HCl (0.9998 mmol) were dissolved in deionized water (1 mL). The pH of the resulting solution was adjusted to pH = 1 with standardized aqueous HCl (16.35 µL, 5.93 M).

Feed solution 2 consisted of pure *n*-decane.

Feed solution 3 was prepared using MPAA (672.6 mg, 3.998 mmol) and $Na_2HPO_4{\cdot}2H_2O$ (357.1 mg, 2.006 mmol). These reagents were dissolved in water (8.3 mL) and the pH of the resulting solution was adjusted to 7.80 with standardized aqueous NaOH (1.7 mL, 4.43 M).

Feed solution 4 consisted of standardized aqueous NaOH. Depending on the pH range, 0.42 M or 0.53 M $NaOH_{aq}$ solutions were utilized for pH 7.45-7.77 and for pH 7.85-8.23, respectively.

In a typical procedure, the system was operated at steady state using Feeds 1-4 as described above. The flow rates for Feeds 1,2 were set at $\phi_{1,2}$ = 3.3 µL min^{-1}. Optimization of the pH in the second microreactor was performed by subsequently varying the flow rate of either Feed solution 3 (ϕ_3, MPAA) or Feed solution 4 (ϕ_4, NaOH). First trials involved Feed 3 only, and the corresponding flow rate (ϕ_3) was varied from 26.4 to 40 µL min^{-1}, while Feed 4 was off-line (ϕ_4 = 0 µL min^{-1}) (pH 7.19 to 7.37, green dots in **Supplementary Figure 23**. Then, ϕ_3 was set to 35 µL min^{-1} and Feed 4 was set on-line. For the 7.45 to 7.77 pH range (red dots in **Supplementary Figure 23**), Feed 4 consisting in 0.43 M $NaOH_{aq}$ was injected at a flow rate ϕ_4 ranging from 2 to 3.5 µL min^{-1}. Next, Feed 4 was changed to 0.53 M $NaOH_{aq}$, and flow rate ϕ_4 was varied from 3.2 to 4 µL min^{-1} (pH 7.85 to 8.23, blue dots in **Supplementary Figure 23**).

Optimization of the residence time in the second microreactor μF²

Please refer to **Supplementary Figure 17** for the microfluidic assembly used for this experiment.

Optimization of the residence time for the cyclization process within the second microreactor was conducted with peptide **4c** by varying the length of the tubing accordingly (tubing 1/16” e.d., 500 μm i.d.). The concentration of the peptide in the feed was 14 mM.

The data are presented in **Supplementary Figure 24** and **Supplementary Table 5**.

Suppression of dimeric species formation

Dimeric linear or cyclic species were identified in the crude of peptide **7c** when the microfluidic system was fed with a 14 mM solution of peptide **4c** (see **Supplementary Figure 24**). The structures shown in **Supplementary Figure 25** are in agreement with the molecular ions detected by LC-MS for these side-products. A two-fold dilution of the feed solution 1 (7 mM) suppressed the formation of these side-products.

*Optimized procedure under microfluidic conditions for SEAE peptide **5** synthesis*

Please refer to **Supplementary Figure 16** for the microfluidic assembly used in this experiment. The procedure is illustrated with the synthesis of **5a**.

0.2 M TCEP.HCl (57.4 mg, 0.200 mmol) was dissolved in water (1 mL). 6 M HCl (14 µL) was added to adjust the pH to 1.04. Peptide **3a** (20.31 mg, 14.00 µmol, 14 mM) was dissolved in the above solution (1 mL) and injected into the microfluidic system described in **Supplementary Figure 16** (1 m tubing, 500 µm diameter, 3.3 µL min^{-1}, residence time 1 h, 90 °C). After 1 h, the solution was collected (600 µL), diluted with 0.6% TFA pH=1 (3 mL) and purified by HPLC to yield 7.9 mg of peptide **5a** (60%). Conditions: C18XBridge column, 0 to 10% eluent B in 5 min, then 10 to 30% eluent B in 60 min, eluent A = water containing 0.1% TFA, eluent B=acetonitrile in water 4/1 v/v containing 0.1% TFA, 30 °C, detection at 215 nm, 6 mL min^{-1}.

The results for the synthesis of SEAE peptides **5a-c** are presented in **Supplementary Table 7**. A comparison of the activation of peptide **4c** into **5c** in batch or under microfluidic conditions is shown in **Supplementary Figure 39** (see also **Supplementary Table 6**).

Optimized procedure under microfluidic conditions for peptide cyclization

Please refer to **Supplementary Figure 17** for the microfluidic assembly used in this experiment.

Feed solution 1 (SEA^{on} peptide **4**). 7 µmol of the starting SEA^{on} peptide **4** (see **Supplementary Table 8** for model peptides**, Supplementary Table 9** for **RTD-1** peptides) and 28.66 mg of TCEP.HCl (0.9998

mmol) were dissolved in deionized water (1 mL). The pH of the resulting solution was adjusted to pH = 1 with aqueous HCl (16.35 µL, 5.93 M).

Feed solution 2 (decane). Feed solution 2 consisted of pure n-decane.

Feed solution 3 (MPAA). For the manipulations on peptides **4c-k**, 672.6 mg of MPAA (3.998 mmol) and 357.1 mg of $Na_2HPO_4 \cdot 2H_2O$ (2.006 mmol) were dissolved in water (8.3 mL). The pH of the resulting solution was adjusted to 7.80 with aqueous NaOH (1.7 mL, 4.43 M).

For cyclization of peptide **4p**, 5.730 g Gn.HCl (59.98 mmol), 672.6 mg of MPAA (3.998 mmol) and 357.1 mg of $Na_2HPO_4.2H_2O$ (2.006 mmol) were dissolved in water (4.3 mL). The pH of the resulting solution was adjusted to 7.60 (apparent value measured with a regular glass pH electrode) with aqueous NaOH (1.7 mL, 4.43 M).

For the synthesis of **RTD-1** and for the cyclization of peptides **4o,p**, 5.730 g Gn.HCl (59.98 mmol), 672.6 mg of MPAA (3.998 mmol) and 357.1 mg of $Na_2HPO_4.2H_2O$ (2.006 mmol) were dissolved in water (4.3 mL). The pH of the resulting solution was adjusted to 7.60 (apparent value measured with a regular glass pH electrode) with aqueous NaOH (1.7 mL, 4.43 M).

The first microfluidic coil (μF^1) was constructed from 2 m of PFA capillary (1.58 mm O.D., 500 µm I.D.; 0.4 mL internal volume). The temperature of μF^1 was set at 90 °C or 65 °C depending of the C-terminal amino acid (see **Supplementary Table 8** for model peptides and **Supplementary Table 9** for RTD-1 peptides). The second microfluidic coil (μF^2) was constructed from 0.84 m of PFA capillary (1.58 mm O.D., 500 µm I.D.; 0.17 mL internal volume). The temperature of μF^2 was set at 37 °C or 45 °C (see **Supplementary Table 8** for model peptides and **Supplementary Table 9** for RTD-1 peptides). The back-pressure regulator was set at 2.5 bar. The solution of the desired starting SEA^{on} peptide was loaded in a 1 mL glass syringe (Feed 1). A second glass syringe was loaded with *n*-decane (Feed 2). The MPAA stock solution was prepared as described above and, loaded in a 10 mL syringe. The syringe pumps injecting Feeds 1 and 2 were set at 3.3 µL min^{-1}, and the syringe pump injecting Feed 3 was set at a flow rate of 35 µL min^{-1}. The reactor effluent was collected at steady state after an equilibration phase of 1.5 residence time.

The effluents coming from μF^1 only (production of **5**, activation step) or μF^1 and μF^2 (activation and cyclization into 7) were analysed by HPLC to determine the yields. The data are collected in **Supplementary Table 10**.

Depending on the cyclic peptide synthesized, the work up procedure 1 or 2 and 1 was applied before HPLC purification (indicated in **Supplementary Table 11** and **Supplementary Table 12**).

Work up 1 (MPAA extraction and HPLC purification) was used when the starting SEA^{on} peptide and the target cyclic peptide were well separated by HPLC. For workup 1, the solution was quenched with an excess of 10% aqueous acetic acid. MPAA was extracted 5 times with diethylether (1.5 mL). The aqueous solution was analyzed by RP HPLC or LC-MS and subsequently purified by HPLC.

Work up 2 was applied before work up 1 when the starting SEA^{on} peptide and the target cyclic peptide were not separated by HPLC. The line coming from the microfluidic system was inserted in an epperdorf tube containing the solution of AcA-MPA **8** (1.2 eq., produced as described elsewhere [8]) and TCEP.HCl (100 mM) in sodium phosphate buffer (0.2 M, pH 7.4, pH=7.4, 1 mL). After collecting the effluent from the microfluidic system, the Eppendorf tube was placed under nitrogen atmosphere overnight.

The cyclic peptides were purified by HPLC. Conditions: C18XBridge column, 0 to 20% eluent B in 5 min, then 20 to 50% eluent B in 90 min, eluent A = water containing 0.1% TFA, eluent B = acetonitrile in water 4/1 containing 0.1% TFA, 30 °C, detection at 215 nm, 6 mL min^{-1}.

*Microfluidic experiment with MPA peptide thioester CILKEPVHGV-MPA **1c***

The aim of this experiment was to compare MPA peptide thioester CILKEPVHGV-MPA **1c** and SEA peptide **4c** for the microfluidic production of cyclic peptide **7c**.

Although the internal proline reduces the efficiency of peptide cyclization as discussed in the manuscript, this peptide sequence was utilized since linear precursor **4c** and cyclic peptide **7c** are separated by HPLC, thus enabling the direct determination of a conversion by HPLC (workup 2 was not necessary as for some other precursors).

For MPA peptide thioester CILKEPVHGV-MPA **1c**, the optimal microfluidic system was adapted since microfluidic module μF^1 was not necessary. MPA peptide thioester CILKEPVHGV-MPA **1c** was dissolved at 7 mM at pH 1 and the corresponding solution was used to feed the microfluidic system depicted in **Supplementary Figure 18**. Feed 2 consisted of decane, and Feed 3 consisted of a solution of MPAA at pH 7.8. The relative flow rates and other relevant reaction parameters are indicated in **Supplementary Figure 18**.

The result of this experiment is depicted in **Supplementary Figure 26**. The conversion of MPA peptide thioester **1c** into cyclic product **7c** was only 11% after 4 min of residence time. Moreover, the product **7c** was contaminated with dimeric species and MPAA peptide thioester **2c**.

*Microfluidic experiments with MPAA peptide thioester **2c** (CILKEPVHGV-MPAA)*

The microfluidic experiment was performed by adapting the optimal microfluidic setup as already described for MPA peptide thioester **1c** (see *Microfluidic experiment with MPA peptide thioester CILKEPVHGV-MPA **1c***, **Supplementary Figure 18)**.

The analysis of the effluent after 4 min of residence time in μF^2 is shown in **Supplementary Figure 28**. This experiment shows that cyclization is not complete since MPAA peptide thioester **2c** is still present in the crude after 4 min of residence time. The formation of some dimeric species was also observed.

Importance of MPAA for the cyclization

In this experiment, peptide **5c** was dissolved at pH 1 at a final concentration of 7 mM. These batch conditions mimic the peptide solution coming from μF^1 in the optimal conditions used for the microfluidic syntheses (see **Supplementary Figure 17**). Then, the solution was diluted with pH 7.8 phosphate buffer without MPAA. The final pH after mixing the two solutions was 7.17. The resulting mixture was analyzed by LC-MS.

Preparation of solution 1 (peptide solution). TCEP.HCl (2.89 mg, 10.1 µmol) was dissolved in deionized water (100 µL). The pH of the resulting solution was adjusted to pH = 1.04 with aqueous HCl (1.3 µL, 6 M). The starting SEAE peptide **5c** (1.035 mg, 0.6199 µmol) was dissolved in the above solution (86 µL, 7 mM).

Preparation of solution 2 (phosphate buffer without MPAA). $Na_2HPO_4.2H_2O$ (35.8 mg, 0.2011 mmol) was dissolved in water (990 µL). The pH of the resulting solution was adjusted to 7.80 with aqueous HCl (5 µL, 6 M) and NaOH (5 µL, 4.43 M).

The peptide solution 1 (86 µL) was quickly added to solution 2 (907 µL) under agitation at 37 °C. The final concentrations were: peptide **5c** 0.66 mM, TCEP 9.4 mM, Na_2HPO_4 19.9 mM.

Aliquots (10 µL) were analyzed after 4 min, 15 min and 45 min of reaction. For this purpose, the aliquots were quenched with aqueous acetic acid (10% v/v, 100 µL) and injected in an LC-MS system equipped with a C18XBridge column.

The result of this experiment is shown in **Supplementary Figure 4**. This experiment showed that after 4 min the starting SEAE peptide **5c** is converted into SEA peptide **4c**. No cyclized peptide **7c** could be detected in the crude. In conclusion, MPAA is mandatory for the cyclative ligation.

Stability of the optimized microfluidic system

The stability of the optimized microfluidic system was studied using SEA^{on} peptide **4c** (see **Supplementary Figure 17)**. The effluent was collected at different time points from 0 to 3.5 h and analyzed by LC-MS (workup 1).

The result of this experiment is shown in **Supplementary Figure 29**. The data show the high stability of the microfluidic system over time.

*Comparative study of batch vs microfluidic cyclization protocols with SEA peptide **4c***

Experiments under batch conditions were performed with peptide **4c** at different scales as depicted in **Supplementary Figure 5**. The same batch of peptide **4c** was used for all the experiments under batch or microfluidic conditions.

The LC-MS analyses for these batch experiments are compared to a typical LC-MS of a microfluidic experiment using peptide **4c** for the feed solution. The data presented in **Supplementary Figure 6** and **Supplementary Figure 7** show a large effect of the scale of synthesis for batch experiments, while the microfluidic system sustained a homogeneous and consistent purity profile (see **Supplementary Figure 29**). Moreover, batch experiments showed that residual MPAA peptide thioester **2c** contaminated the target cyclic peptide **7c**. This is clearly visible for batch experiments performed at 10 and 25 mg scale in the chromatograms shown in **Supplementary Figure 6**. The ion current for MPAA peptide thioester **2c** (double charge species) for batch and microfluidic experiments was integrated, and the corresponding values are compiled in **Supplementary Figure 7** for the sake of comparison. The signal for the microfluidic experiment is close to background.

Compatibility of Asn-Gly or Asp-Gly dipeptide units with the activation step

Peptide **4e** was rearranged into SEAE peptide **5e** in batch (90 °C for 1 h) and directly analyzed by LC-MS (**Supplementary Figure 8**). This peptide was not isolated.

Peptide **4f** was rearranged into SEAE peptide **5f** in batch (90 °C or 65 °C for 1 h) and directly analyzed by LC-MS. This peptide was not isolated. Peptide **5f** is partially degraded at 90 °C (**Supplementary Figure 9A**), while degradation is almost suppressed at 65 °C (**Supplementary Figure 9B**). In the latter case however, the conversion of peptide **4f** into SEAE peptide **5f** is lower (~50%).

Peptide **4g** was rearranged into SEAE peptide **5g** in batch (65 °C for 1 h) and directly analyzed by LC-MS (**Supplementary Figure 10**). This peptide was not isolated.

Peptide **4h** was rearranged into SEAE peptide **5h** in batch (65 °C for 1 h) and directly analyzed by LC-MS (**Supplementary Figure 11**). This peptide was not isolated

Intermolecular ligation of a peptidyl prolyl SEAE peptide in the optimized microfluidic system

For this experiment, we used peptide ILKEPVHGP-SEA^{on} **4q** and CILKEPVHGV-NH_2 **10** to synthesize peptide ILKEPVHGPCILKEPVHGV-NH_2 **11** using the optimized microfluidic setup with the following parameters:

µF^1: T = 90 °C, t^1 = 60 min ; µF^2: T = 37 °C, t^2 = 15 min

The results are presented in **Supplementary Table 13**. The characterization purified peptide **11** can be found in **Supplementary Figure 94** and **Supplementary Figure 95**.

Folding of RTD-1 peptide

RTD-1 produced from **4l** (2.10 mg, 0.790 µmol, 1.02 mM) was dissolved in 1% AcOH in water (714 µL). The pH was adjusted to 7.52 by adding 5 M NH_4OH (60 µL). The air oxidation proceeded overnight at ambient temperature. After 23 h, the reaction mixture was diluted with 10% AcOH (3 mL) and purified by HPLC to produce 0.6 mg of folded RTD-1 peptide (29%). Conditions: C18XBridge column, 0 to 15% eluent B in 5 min, then 15 to 30% eluent B in 45 min, eluent A = water containing 0.1% TFA, eluent B=acetonitrile in water 4/1 containing 0.1% TFA, 50 °C, detection at 215 nm, 3 mL min^{-1}.

For the characterization of folded RTD-1 peptide see **Supplementary Figure 96**, **Supplementary Figure 97** and **Supplementary Figure 98**.

Antibacterial assay

Antibacterial assays were performed as described previously.[9] Briefly, *E. coli* BW25113 (kindly provided by Coli Genetic Stock Centre, CGSC), and *S. aureus* SH1000 (kindly provided by Prof. S.J. Foster, University of Sheffield) were grown overnight in Cation-Adjusted Mueller Hinton II Broth (37°C, 150 rpm). Cultures were then diluted 1 in 20 and grown for 2 h (37°C, 150 rpm) to get a log phase bacterial culture. Log phase cultures were then pelleted (3000 × g, 5 min) and washed twice in 10 mM PIPES (pH 7.4) with 5 mM glucose. The bacterial suspension was diluted in 10 mM PIPES (pH 7.4) with 5 mM glucose to an OD600 of 0.00125. 150 µL of the bacterial suspensions were added to the wells of a 96 well plate. 3 µL of RTD-1 serially diluted in water (at 50 × final concentration, 800-25 µg mL^{-1}) was then added to the bacteria to give a final concentration range of 16-0.25 µg.mL^{-1}. Bacteria viability was then determined by counting colony-forming units following plating on LB agar.

The experiment was done in triplicate. The results are presented in **Supplementary Table 14**.

Supplementary Note

We have observed that RTD-1 peptide (reduced) has a tendency to partially decompose during MALDI-TOF analysis in reflector mode. The decomposition results in the appearance of a peak at -27.5 mass units. The decomposition is much less pronounced in linear mode. Therefore, for some of

the compounds we present both types of analyses (as for RTD-1 produced from **4n**, **Supplementary Figure 89** and **Supplementary Figure 90**). In linear mode, the peak appears at -33 mass units, suggesting that the decomposition is due to a loss of HS. In contrast, the decomposition is not observed in the RTD-1 Ala variant **7o** (**Supplementary Figure 92** and Supplementary **Figure 93**), and also in the folded RTD-1 peptide (**Supplementary Figure 97** and **Supplementary Figure 98**), suggesting that the cluster of cysteine residues present in RTD-1 is responsible for this behavior.

Supplementary References

1 Ollivier, N., Dheur, J., Mhidia, R., Blanpain, A. & Melnyk, O. *Bis*(2-sulfanylethyl)amino native peptide ligation. *Org. Lett.* **12**, 5238-5241 (2010).

2 Nagy, K. D., Shen, B., Jamison, T. F. & Jensen, K. F. Mixing and dispersion in small-scale flow systems. *Org. Proc. Res. Dev.* **16**, 976-981 (2012).

3 Sniady, A., Bedore, M. W. & Jamison, T. F. One-flow, multistep synthesis of nucleosides by Bronsted acid-catalyzed glycosylation. *Angew. Chem. Int. Ed.* **50**, 2155-2158 (2011).

4 Boll, E. *et al.* Access to large cyclic peptides by a one-pot two-peptide segment ligation/cyclization process. *Org. Lett.* **17**, 130-133 (2015).

5 Dheur, J., Ollivier, N., Vallin, A. & Melnyk, O. Synthesis of peptide alkylthioesters using the intramolecular *N,S*-acyl shift properties of *bis*(2-sulfanylethyl)amido peptides. *J. Org. Chem.* **76**, 3194-3202 (2011).

6 Robertson, E. G. *et al.* IR spectroscopy of physical and chemical transformations in cold hydrogen chloride and ammonia aerosols. *Phys. Chem. Chem. Phys.* **11**, 7853-7860 (2009).

7 Gray, W. R. Disulfide structures of highly bridged peptides: a new strategy for analysis. *Protein Sci.* **2**, 1732-1748 (1993).

8 Ollivier, N. *et al.* A simple and traceless solid phase method simplifies the assembly of large peptides and the access to challenging proteins. *Chem. Sci.* **8**, 5362-5370 (2017).

9 Tang, Y. Q. *et al.* A cyclic antimicrobial peptide produced in primate leukocytes by the ligation of two truncated alpha-defensins. *Science* **286**, 498-502 (1999).
